# Expanding the Biocatalytic Toolbox: A Singular P450 Enzyme Efficiently Produces Diverse Pharmaceutical Metabolites

**DOI:** 10.1021/acsomega.6c01534

**Published:** 2026-08-12

**Authors:** Laura N. Jeffreys, Sian Thistlethwaite, Matthew J. Cliff, Harshwardhan Poddar, Sahara Bhanot, Richard B. Tunnicliffe, Marina Golovanova, Katherine Hollywood, Colin W. Levy, Michael W. Voice, David Leys, Kirsty J. McLean, Jonathan P. Waltho, Andrew W. Munro, Hazel M. Girvan

**Affiliations:** † Manchester Institute of Biotechnology, 5292 University of Manchester, Manchester M1 7DN, U.K.; ‡ Cypex Ltd., 6 Tom McDonald Avenue, Dundee DD2 1NH, U.K.; § Department of Physical and Life Sciences, School of Applied Sciences, University of Huddersfield, Queensgate, Huddersfield HD1 3DH, U.K.; ∥ School of Biosciences, University of Sheffield, Firth Court, Western Bank, Sheffield S10 2TN, U.K.

## Abstract

P450 BM3 is a natural fusion protein with a P450 domain fused to its necessary redox partner and capable of binding a range of fatty acids at high catalytic rates. The flexibility of the active site and catalytic efficiency have led to extensive research aimed at modifying substrate and product profiles for its utilization as a biocatalyst. The introduction of a double mutation (DM) at the base of the active site (A82F/F87V) allows the binding of a broad range of structurally diverse small molecules, including pharmaceutical drugs. Herein, we describe screening with an FDA-approved drug compound library, and the results exhibit the promiscuous nature of the DM BM3 variant. Of the 978 compounds screened; 59% of the library elicited UV–vis spectral shifts indicative of binding with a range of structurally diverse drugs. The interaction of the DM BM3 variant with a range of structurally diverse drugs, with a particular focus on the antidiabetic glitazone (thiazolidinedione) class, was investigated to observe ligand binding modes and metabolite production. BM3-derived metabolites were successfully produced and structurally elucidated for several structurally diverse drugs in the steroid, glitazone, meglitinide, terpene lactone, retinoid, and fibrate classes. The range of modifications performed includes some that are human P450-derived metabolites. Structural elucidation of the troglitazone-bound DM variant P450 domain shows an unusually close crystallographic interaction between the substrate and heme cofactor. We report here the use of the DM BM3 variant as a model for the main human metabolizing P450 enzymes CYP1A1, CYP2B6, CYP2C8, CYP2C9, and CYP3A4, as well as a biotechnological tool for the production of metabolites with diverse functionality.

## Graphical abstract

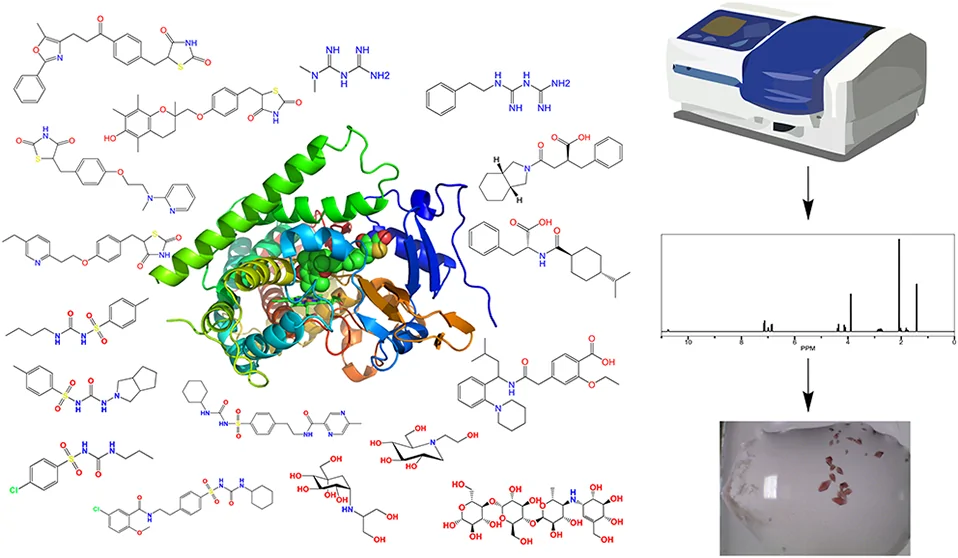

## Introduction

In recent years, there have been significant efforts made to develop alternative biosynthetic routes for the production of existing and new pharmaceuticals in a sustainable and cost-efficient manner.^1,2^ Health governing bodies such as the US Food and Drug Administration (FDA) now require by law a full ADME profile of new drugs to progress to phase I clinical trials. This profile includes structurally elucidating drug metabolites produced at 10% or more of the level of the parent compound and determining the *in vivo* effects. As ∼70% of pharmaceutical drugs are metabolized by the CYP1, CYP2, and CYP3 families,^3^ substantial research is undertaken to understand the interaction of a drug with CYP enzymes (also referred to as P450s) before the drug can reach the market. Due to the relatively slow catalytic rates of human CYPs, this can be a laborious process to yield enough metabolite for ADME studies. Utilization of catalytically efficient bacterial CYPs for the production of human-derived P450 metabolites could lead to more cost-efficient production.

P450 BM3 (BM3 or CYP102A1) is a soluble cytochrome P450 monooxygenase enzyme isolated from *Priestia megaterium* (formerly *Bacillus megaterium*).^4–6^ BM3 is a natural fusion enzyme containing a heme-binding catalytic domain (∼55 kDa) linked to a cytochrome P450 reductase domain partner (∼65 kDa).^7,8^ This structural arrangement facilitates rapid electron transfer, leading to high catalytic rates (e.g., >17,000 min^–1^ with arachidonic acid).^9^ BM3’s natural substrates are fatty acids with chain lengths ranging from C12–C18.^5,10^ Due to its structural similarity to drug-metabolizing human P450 isoforms, BM3 has been engineered to bind and metabolize a wide range of structurally diverse non-native substrates while exhibiting a diverse range of biotransformations.^11^ Notable examples include the production of the antimalarial drug artemether^12^ and the synthesis of the high-value grapefruit fragrance molecule nootkatone.^13^ Numerous steroid biotransformations have been demonstrated, including production of neuroprotective steroidal C7β-alcohols^14,15^ or 15β selectivity.^16,17^ Canonical reactivities have been pushed toward carbene and nitrene transfer reactions by modifications, including to the proximal thiolate.^200,18^ Computational approaches combined with site-saturation mutagenesis have been utilized to achieve N-oxidation.^18^ Production of BM3 metabolites has been utilized in commercial settings, for example, the production of the retinoid precursor 4-hydroxyisopherone on a kilogram scale.^19^ The ability of BM3 variants to metabolize structurally diverse compounds to yield products with diverse functionality makes them a key tool in the future of biosynthetic drug production. However, BM3 variants produced previously in enzyme libraries often display a limited catalytic repertoire, which is advantageous for selective metabolite production but limiting for the characterization of BM3-produced human P450 metabolites for preclinical trials and for pharmaceutical library diversification for drug discovery.^2^ In several examples, mutagenesis to achieve high specificity toward a desired metabolite has required several mutations, which can lead to protein instability.^17,20^

In earlier studies, we have utilized a double mutant “gatekeeper” variant of BM3 (BM3 DM) to target structurally diverse FDA-approved drug compounds for metabolite production. BM3 DM includes two strategic point mutations (A82F/F87V) in the active site, which ‘lock’ the heme domain in an open, flexible conformation by removing steric bulk from the active site (F87V =) and facilitating conformational changes that allow BM3 to adopt the catalytically primed state, even when substrate-free (A82F).^21–23^ Drug metabolites were successfully produced using BM3 DM for the proton pump inhibitor omeprazole, including two major human CYP2C19-derived metabolites.^22,23^

In this paper, we screened drugs from a structurally diverse 978-compound FDA-approved pharmaceutical library for BM3 DM substrate binding. We observed that 59% of the compounds screened induced spectral changes, suggesting ligand binding. We then selected promising candidates from structurally diverse drug classes for further investigation into their binding affinity constants, binding modes, and product formation with a particular focus on the tight-binding antidiabetic glitazone and meglitinide classes. Troglitazone showed tight binding in UV–vis and electron paramagnetic resonance (EPR) spectroscopy studies, suggesting an unusual binding mode for P450 BM3. Crystallographic studies elucidated the structure of troglitazone bound to the DM BM3 variant. Orientation of the compound in the active site was consistent with metabolite production and similar to the interactions observed with other pharmaceutical compounds such as omeprazole.^23^ These data provide evidence that BM3 DM can act as a model enzyme for human P450-derived metabolite production as well as a promiscuous biotechnological tool to be utilized in functional diversification of pharmaceutical libraries.

## Materials and Methods

### BM3 Protein Expression and Purification

The BM3 (UniProt M9TKI6) WT and DM heme domain and intact proteins (both his-tagged and non-his-tagged) were expressed as described in our previous studies.^17,21,22,24^ Briefly, pET14b (his-tagged heme domain), pET15b (his-tagged full-length), and pET20b (nonhis-tagged heme domain) plasmid constructs were transformed into BL21 (DE3) and expressed in Terrific Broth medium (Formedium, Hunstanton, UK) for the heme domain and in Auto Induction Terrific Broth (Formedium) for the full-length protein.

For his-tagged BM3 full-length and heme domains, purification was achieved in two steps using a nickel-IDA column, followed by an anion exchange (hydroxyapatite) column. Purified protein was then dialyzed into 100 mM KPi and flash frozen in liquid nitrogen for storage at −80 °C.

For the truncated BM3 DM heme domain, a construct lacking the his-tag (pET20b) was used to improve crystallogenesis and structural resolution. A four-column purification (three anion exchange and one size-exclusion chromatography column) was utilized to achieve the high purity needed for X-ray crystallography. Full details of this method are given in previous work.^21,24^

In a final step prior to utilization in experimental procedures, lipids were removed from BM3 full-length and heme domain active sites using the hydrophobic resin Lipidex-1000 and the relevant experimental buffer. Heme protein concentration was quantified using the absorbance of the low-spin P450 Soret peak (∼418 nm) with the extinction coefficient 95 mM^–1^ cm^–1^ for the heme domain and 105 mM^–1^ cm^–1^ for the full-length P450 BM3 protein.

### FDA-Approved Drug Screening Library

A screen was acquired, containing 978 FDA-approved compounds in 10 mM stocks (100% DMSO) from SelleckChem (L1300). The screen contained a wide range of structurally diverse compounds for different drug targets with molecular weights ranging from 100 to 800 g/mol. In a quartz cuvette, 150 μM of ligand (1.5% (v/v) DMSO) was added to 3 μM protein in 100 mM KPi, pH 7 (total volume 500 μL), and Soret peak shifts were observed on an Agilent Cary 50 UV–vis spectrophotometer (Agilent, Stockport, UK). The high stoichiometry was to ensure saturation and the determination of any potential binding. In some cases, the ligand was colored or had poor solubility, leading to increased absorbance across the spectrum. A range of type I substrate shift, inhibitors, no-shift, and compounds causing protein depletion (loss of Soret peak indicative of the heme prosthetic group leaving the protein, likely due to protein unfolding) were observed. For type II shifts (418 nm to 420–427 nm) elicited by inhibitors, the wavelength of the Soret peak shift was recorded. For type I shifts (418 to 390 nm) elicited by substrate binding, the percentage spin shift was calculated using a modified high-spin calculation eq (eq 1).^25^ The extinction coefficients used were derived from BM3 DM using N-palmitolyglycine (NPG) as a model for 100% substrate binding. For the results described here, the percentage spin shift for DMSO (3.5%) was subtracted. A Python script was produced to extract the necessary absorbance maxima from CSV files and input them into the Soret peak shift calculation (eq 1). The script was produced to find the closest wavelength to 393/394 nm and 417/418 nm in all cases. In addition, the script produced graphical images for manual determination of turbidity, protein aggregation, or other causes of error.

Percentage type I Soret peak shift equation for DM BM3:
1

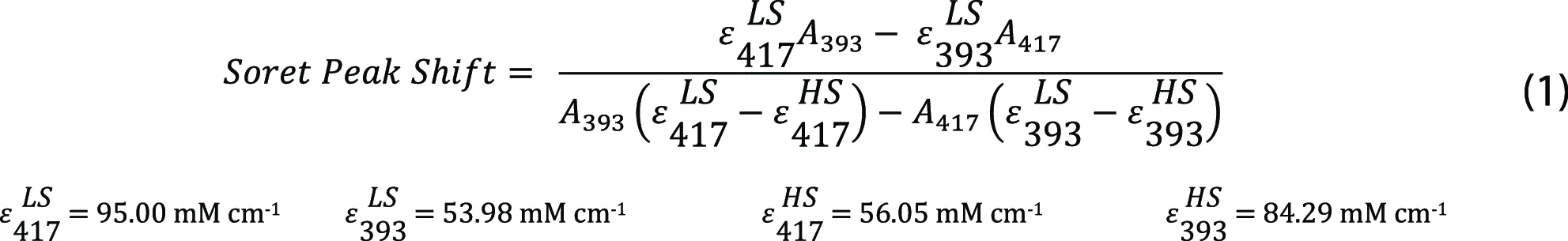




### Drug Binding Affinity Determination

The binding affinities (*K*_d_ values) of selected compounds (see Table 1) were determined using the BM3 WT and DM heme domains. All compounds were sourced from Sigma-Aldrich (Haverhill, Cambridgeshire, UK). An Agilent Cary 60 UV–vis spectrophotometer (Agilent, Stockport, UK) with a Peltier at 30 °C was used for data collection. Darglitazone absorbance titrations were conducted on an Agilent Cary 300 dual beam spectrophotometer (Agilent, Stockport, UK) with a reference cell containing 100 mM KPi (pH 7) and the same addition of ligand as made to the sample cell due to compound absorbance in the UV–vis range. Titrations were undertaken with 3 μM protein in a quartz cuvette (paired in the case of darglitazone with a cuvette with ligand but no protein) by microliter additions of concentrated drug ligands (in DMSO) until saturation was achieved. *K*_d_ values were calculated using a rectangular hyperbolic equation^26^ for hyperbolic curves, the Morrison equation^27^ for tight-binding ligands (where *K*_d_ values are typically ≤ 5x the P450 concentration), or the Hill equation^28^ for sigmoidal curves.

**Table 1:** Binding Affinities for BM3 DM and WT Heme Domains with Structurally Diverse Drug Compounds^a^

FDA compound	Drug Class	MW (g/mol)	3 μM BM3 DM + 150 μM ligand soret peak shift (%)	WT BM3 *K*_d_ (μM)	DM BM3 *K*_d_ (μM)
Artemisinin	Terpene Lactones	298.4	41.0	NB	15.8 ± 0.90
Drospirenone	Steroids	366.5	45.8	NB	11.3 ± 0.10
Estradiol	Steroids	272.4	38.1	NB	2.22 ± 0.23
Darglitazone*	Glitazones	420.5	N/A	17.40 ± 0.56	1.15 ± 0.07
Pioglitazone	Glitazones	356.4	52.3	NB	2.01 ± 0.09
Rosiglitazone	Glitazones	357.4	55.7	NB	0.85 ± 0.15
Troglitazone*	Glitazones	441.5	N/A	NB	0.04 ± 0.01
Mitiglinide*	Meglitinides	315.4	N/A	NB	1.17 ± 0.08
Nateglinide	Meglitinides	317.4	15.6	NB	5.08 ± 0.30
Repaglinide	Meglitinides	452.6	46.6	NB	12.5 ± 0.86
Metformin*	Biguanides	129.2	N/A	NB	0.43 ± 0.61

a Many drugs were shown to bind to the BM3 WT and DM heme domains by eliciting a type I Soret spectral shift. The table shows examples of such molecules and the relevant *K*_d_ values determined for these compounds by optical titration. Only darglitazone elicited a type I spectral shift with the BM3 WT heme domain, with a much lower binding affinity than for the BM3 DM heme domain; all other compounds showed no spectral binding (NB) to the WT. Asterisks (*) indicate compounds that were not in the initial screening library.

### Compound Turnover and Product Determination by HPLC, LCMS/MS, and NMR

Reactions were monitored for metabolite formation over 180 min. A cofactor regeneration system (7.76 mM glucose-6-phosphate, 0.6 mM NADP^+^, and 0.75 U/mL G6P dehydrogenase) and 5 μM BM3 DM full-length were made up in buffer B to a total volume of 60 mL. BM3 WT and relevant controls were made up to a total volume of 5 mL. All reactions were preincubated at 37 °C, 60 rpm for 5 min before the addition of the relevant ligand (1% [v/v] DMSO, 100 μM). A 0.5 mL aliquot was taken and immediately quenched in 200 μL 37% HCl. Subsequent samples were taken at 1, 2, 3, 4, 5, 10, 15, 20, 40, 60, and 180 min. All controls were quenched at 180 min in 200 μL 37% HCl. The quenched samples were neutralized to pH 7 with saturated sodium hydroxide and centrifuged in an Eppendorf 5810R centrifuge (4000 rpm, 4 °C, 20 min) before removing the supernatant.

For each sample, 300 μL supernatant was removed and added to 300 μL 100% HPLC-grade acetonitrile containing internal standard phenacetin (final concentration 50 μM) in preparation for LCMS/MS analyses. In all cases, the parent compound was used as a standard except for repaglinide. For repaglinide, we used the parent compound or 21-hydroxy-repaglinide (purchased as 3-hydroxy-repaglinide but renumbered to match the literature). UPLC-MS/MS full scan analyses (scan range 50–1000 *m*/*z*) were completed on a Q Exactive Plus instrument equipped with a heated electrospray ionization source and a U3000 UHPLC (Thermo Fisher Scientific) using a Hypersil Gold column, 100 × 2.1 mm, 3 μm particle size (Thermo Fisher Scientific). For all samples, 5 μL was injected per run at a flow rate of 0.4 mL min^–1^ with a collision voltage of 75 at 40 °C. An individual method was optimized for each compound to allow for full gradient separation of all metabolites. Repaglinide and rosiglitazone were chromatographically separated in positive ESI mode using a linear gradient of 30–99% Optima LC/MS grade H_2_O + 0.1% FA (solvent A) and Optima LC/MS grade ACN + 0.1% FA (solvent B) over 24.5 min, followed by a hold of 2 min at 100% solvent B before returning to 5% solvent B over 2 min then increasing to 30% solvent B over 2 min. Troglitazone, pioglitazone, fenofibrate, and isotretinoin were chromatographically separated in positive ESI mode using a linear gradient of 5–95% solvent A and solvent B over 15 min, followed by a hold of 2 min at 95% solvent B before returning to 5% solvent B over 2 min. Estradiol was separated in negative ESI mode using a linear gradient of 5–98 Optima LC/MS grade H_2_O + 0.05% ammonium hydroxide (NH_4_OH) (solvent C) and Optima LC/MS grade methanol +0.05% NH_4_OH (solvent D) over 9 min, followed by a hold of 2 min at 98% solvent D before returning to 5% solvent C over 2 min. Artemisinin was separated in positive ESI mode using a gradient of 0–100% 50:50 (v/v) ACN:10 mM ammonium acetate, pH 3.5 (solvent E) and 25:75 (v/v) acetonitrile:methanol (solvent F) over 6 min, followed by a hold of 2 min at 100% solvent F before returning to 0% solvent E over 1 min. Data were analyzed using Thermo Scientific Xcalibur 3.1.66.10 software. Peak and fragmentation chromatograms were graphically represented using OriginPro 9.4.2.380 (OriginLab Corporation). Sample mixtures were prepared for NMR analyses by solid phase extraction (SPE). Strata-X 33 μm and 10 mg/mL Polymeric Reversed Phase tubes (Phenomenex, Macclesfield, UK) were primed with 10 mL of Chromasolv methanol and then washed with 5 mL Chromasolv H_2_O before adding the samples. The columns were then washed sequentially with 3 mL Chromasolv H_2_O and then 2 mL deuterium oxide before drying under nitrogen for 10 min. All products were eluted into 0.7 mL DMSO-d_6_ in HPLC vials and transferred to NMR tubes on the day of analysis. 20 μM TSP was added as a reference peak. NMR spectra were recorded on a Bruker AVIII 500 MHz spectrophotometer with a 1*H*/13C QCI cryoprobe equipped with Z-gradients. 1D 1H NMR spectra were at 298 K using a 1D 1H NMR method with presaturation profiling. From the LCMS/MS peak chromatograms, the time point(s) with all of the metabolites produced were selected for further 2D analyses. All 2D NMR spectra were recorded at 298 K; the following experiments were recorded for each compound standard and selected incubation time point: 1H 13C HSQC multiplicity method edited with gradient selection and adiabatic carbon pulses; magnitude mode COSY with gradients and purge pulses (COSYGPSW); phase-sensitive ROESY (ROESYPHPR.2); 2D TOCSY with excitation sculpting (dipsi2esgpph). For each sample mixture, a combination of identifying and correlating LCMS/MS and 1H NMR peak area changes for each metabolite, selTOCSY experiments to identify the multiplicity of overlapping 1H NMR peaks, and preparative HPLC for very complicated 1D spectra to purify and collect the 1D 1H NMR spectra was used to identify significant metabolites. The pulse sequence ‘seldigpzs’ was used to collect 1D 1H selTOCSY data; standalone peak frequencies were targeted to reveal overlapping peak multiplicities. Data were analyzed using MestReNova 11.0.4 (Mestrelab Research).

For complicated metabolite mixtures, semipreparative HPLC was conducted to yield a metabolite for 1D 1H NMR analyses. Metabolite mixtures for repaglinide, fenofibrate, isotretinoin, and troglitazone were separated on an Agilent 1260 Infinity II LC system with a HypersilODS column (250 × 4.6 mm, 5 μM particle size) with a flow rate of 1 mL min^–1^; peaks were identified by measuring wavelengths at 280 nm. Optimized gradients of HPLC-grade water +0.1% FA (solvent G) and HPLC-grade acetonitrile +0.1% FA (solvent H) were developed to fully separate out each metabolite in the mixtures. The following gradients were used: 30–80% over 40 min for repaglinide, 30–90% over 60 min for troglitazone, 30–95% over 120 min for isotretinoin, and 45–95% over 40 min for fenofibrate. Fractions were collected for each peak elution, and solvent was evaporated under reduced pressure using a vacuum centrifuge at 30 °C. Purified samples were then resuspended in 0.5 mL DMSO-d_6_ containing 20 μM as a reference peak for 1D NMR analyses as above. An aliquot was taken and added to 50:50 water:acetonitrile (1% DMSO) for LCMS/MS analyses as above.

### Electron Paramagnetic Resonance (EPR) Spectroscopy

The binding of ligands to the WT or DM heme domain (in the ferric state) was analyzed using X-band EPR. Samples were prepared with 200 μM protein in 25 mM KPi, pH 7, with the addition of 5 mM compound in DMSO (5% v/v), or appropriate volumes of DMSO or buffer as controls. Samples were incubated overnight at 4 °C with stirring at 10 rpm. The samples were then centrifuged (14,000 rpm for 10 min at 4 °C in a microfuge) before transferring to EPR tubes and freezing in liquid nitrogen. For all data analyzed, a buffer control was subtracted from the spectrum. X-band EPR spectra were recorded on a Bruker ER-300D series electromagnet with a microwave source interfaced with a Bruker EMX control unit and fitted with an ESR-9 liquid helium flow cryostat (Oxford Instruments), and a dual-mode microwave cavity from Bruker (ER-4116DM). Spectra were recorded at 10 K with a microwave power of 2.08 mW and a modulation amplitude of 1 mT.

### Structural Determination by X-ray Crystallography

Samples of ligand-bound DM heme domain were prepared with 9 mg mL^–1^ protein and 5 mM ligand (5% v/v DMSO) in 25 mM KPi, pH 7. Liquid handling was achieved using a Mosquito liquid handling robot (TTP LabTech Ltd., Melbourn, Cambridgeshire, UK) for the addition of the protein to Molecular Dimensions screening plates (PACT, SG1, MORP, JCSG^+^, BCS, Midas, LMB) (Newmarket, Cambridgeshire, UK) and by mixing with the mother liquor provided within the plates. Crystallography was performed using the sitting drop method using 300 nL BM3 DM heme domain protein and 300 nL mother liquor. Seeding was required for improved crystal quality. Seeding was accomplished with 250 nL protein (prepared as above) mixed with 50 nL seed stock (prepared by standard seed bead protocols (Hampton Research, CA, USA)) and 300 nL mother liquor. Plates were stored at 4 °C for crystallogenesis. X-ray diffraction data were collected at Diamond synchrotron beamlines. Data were reduced and scaled using XDS. Structures were solved and refined by molecular replacement with a previously solved BM3 DM heme domain structure (PDB 4O4P) using Phenix,^29^ PDB redo,^30^ and Coot.^31^

## Results

### Promiscuous “Gatekeeper” Variant Binds to 59% of an FDA-Approved Drug Screening Library

The BM3 DM variant was screened against a 978 FDA-approved drug compound library for identification of shifts in the UV–visible spectrum indicative of ligand binding. In the ligand-free form of most P450 proteins, the heme iron is in the low-spin state (s = 1/2). This corresponds to a 6-coordinated iron atom central to the heme group with a water molecule occupying the distal position, and the fully conserved cysteine proximally coordinated. Upon substrate binding, the water molecule is displaced by the ligand, typically causing the iron to adopt a high-spin state (s = 5/2). This binding is visualized as a wavelength shift in the UV–visible absorbance spectrum for the indicative peak of the heme *b* rosthetic group, known as the Soret peak. The substrate-induced Soret peak blue shift is termed as a type I spectra shift (typically from ∼418 nm to ∼394 nm). In comparison, the binding of inhibitors elicits a type II spectral red shift from ∼418 nm to longer wavelengths (∼420–435 nm).^32^ The spectral differences between the two modes of binding are due to many factors, including the bond length between the ligand and heme iron.

Each compound of the library was screened for binding by UV–visible spectroscopy against the DM variant at a 150 μM compound concentration. All compounds were solubilized in DMSO by the manufacturer. For inhibitor (type II) shifts, the wavelength was recorded. For substrate (type I) shifts, the percentage shift was calculated using the shift demonstrated by the commonly used P450 BM3 subtrateubstrate N-palmitoyl glycine as 100% (eq 1).^25^ As DMSO causes a slight shift in the UV–vis spectrum (∼3.5% high-spin), this value was subtracted from all calculated shift values to remove the solvent effect (for more information, see Materials and Methods). At such high concentrations of ligand (150 μM ligand bound to 3 μM protein), it was expected that ‘true’ ligands would cause substantial shifts in the UV–visible spectrum, and so the decision was taken to disregard ligands resulting in shifts of less than 10%. This resulted in 59% of compounds able to produce a significant shift of the Soret peak, indicative of ligand binding (Figure 1 and Table S1). The library was not balanced in terms of mass, logP, or target, and so no structural trends could be deduced (Supplementary Figures S1–S3).

**Figure 1:**
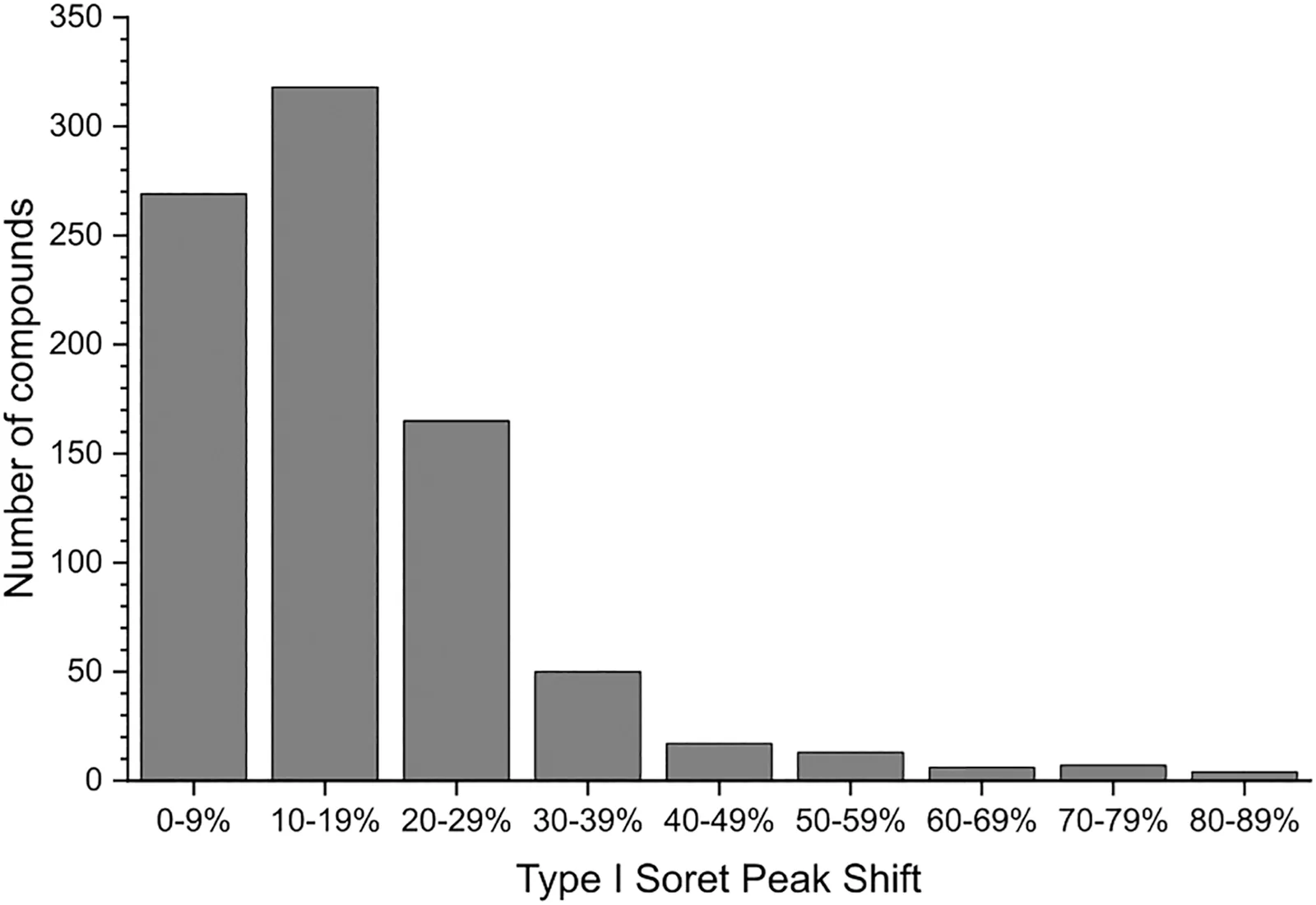
BM3 DM has a wide ligand profile shown by the binding of 59% of compounds within an FDA-approved drug library. The graph shows ligands that produce type I spectral spin-shifts in the DM BM3 heme domain, indicative of substrate binding. The percentage spin-state change is shown with reference to the binding of N-palmitoyl glycine to the DM heme domain, where a near 100% conversion to the high-spin state is observed.

After setting a threshold of 10% Soret peak shift to determine true substrate binding, 518 compounds (52.9% of the compound library) of the 978 compounds tested were found to elicit a type I Soret peak shift, indicative of substrate binding to the BM3 DM heme domain. The majority of the compounds induced small shifts, for example, 318 compounds of the library elicited shifts from 10 to 19%, whereas only 30 compounds elicited shifts of over 50%. Among the substantial number of structurally diverse drugs that elicited type I Soret peak shifts, several drug classes were observed that include but are not limited to steroids, retinoids, terpene lactones, the antidiabetic glitazones and meglitinides, fibrates, and macrolide antibiotics. The four compounds to elicit the greatest shifts at 150 μM were bezafibrate (87.0%), cephalomannine (80.8%), omeprazole (80.5%), and cyclandelate (80.1%). Among these compounds, there was a consistent high percentage shift exhibited by compounds with an antidiabetic function, such as rosiglitazone (55.7%), pioglitazone (52.6%), and repaglinide (46.6%). Despite their shared function, these compounds display differences in various properties, for example, the logP values for rosiglitazone and repaglinide are 2.4 and 5.9, respectively. Repaglinide is a member of the meglitinide drug class, which is characterized by the benzamide moiety responsible for its mode of action. In comparison, rosiglitazone is from the thiazolidinedione drug class, which is characterized by a five-membered C_3_NS ring.

Sixty-one inhibitors were observed with various type II Soret peak shifts observed from 419 to 430 nm, for which prominent classes included azole derivatives and dihydropyridine calcium channel blockers. Forty-five compounds caused protein depletion (PD) during the screening process, which was observed by the reduction of the Soret peak (418 nm) but had no effect on the 280 nm aromatic peak. This was likely due to protein unfolding, leading to the loss of the heme prosthetic group. The classes that caused these changes included diuretics and kinase inhibitors.

### Binding Affinity Determination for Structurally Diverse Drugs Highlighted Key Differences between BM3 WT and DM Proteins

Key compounds from several structurally diverse drug classes were carried forward for binding affinity experiments (Table 1). A particular focus was paid to steroids and antidiabetic compounds, as several steroids, thiazolidinediones (glitazones), and meglitinides were found to elicit large amounts of type I spin shift on the BM3 DM heme domain (Table S1). Additional glitazone and meglitinide compounds were also acquired to further probe the binding to the BM3 DM heme domain, as shown with asterisks in Table 1. Briefly, compounds were added in small volumes to observe the spectral change from ligand-free (low-spin) to ligand-bound (high-spin). Using the difference between the two species, we can calculate a binding affinity for each compound.

In Table 1, all 16 drugs from several structurally diverse drug classes were shown to bind to the BM3 DM heme domain with high affinity. In comparison, only darglitazone bound to the BM3 WT heme domain, eliciting a spectral shift, but the DM variant was still able to bind more tightly with lower *K*_d_ values. Interestingly, metformin contains several nitrogen atoms like well-known P450 inhibitors such as the azole compounds yet elicits a type I (substrate) shift, suggesting an interesting binding mode (Figure 2).^24^ Unfortunately, this binding mode could not be studied further with NMR (due to the requirement for a labeled compound) or X-ray crystallography (due to poor crystallogenesis).

**Figure 2:**
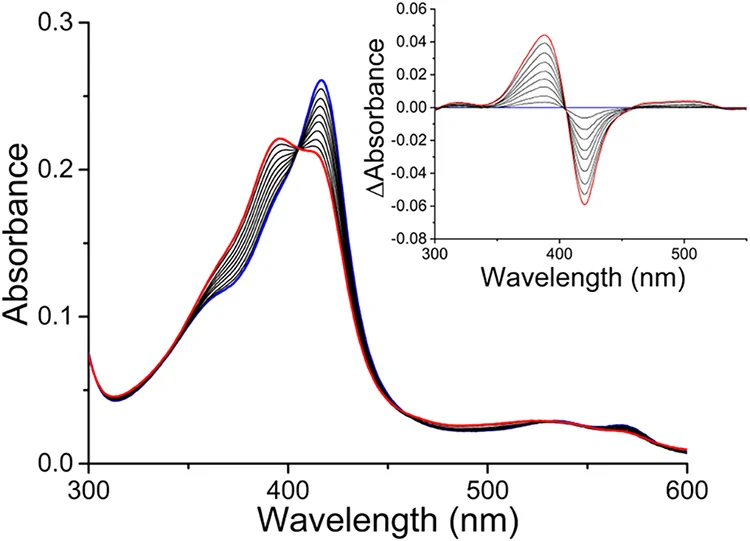
Metformin induces a type I (substrate) shift with the DM BM3 heme domain. The low-spin substrate-free protein with DMSO as a control (blue) gives a Soret shift from 418 nm to a high-spin substrate-bound protein (red) at 395 nm, allowing for binding affinity determination. Metformin was added from a 30 mM stock solubilized in DMSO as 0.1 μL additions with spectral collection following each addition (for clarity of the figure, not all additions are shown). Intermediate spectra can be seen in black. The titration continued until no further spectral changes were observed. Shown in the inset are difference spectra created by subtraction of the starting (DMSO-bound) spectrum from all subsequent spectra, and the red spectrum shows the final spectrum collected in the titration corresponding to 540 μM metformin.

### Product Determination by LCMS/MS and NMR Led to the Identification of a Wide Range of Drug Metabolites

Key compounds from structurally diverse drug classes were carried forward for metabolite production studies based on their high binding affinities for the BM3 DM heme domain and the binding of other members in the same drug class, suggesting a favorable chemical structure for catalysis. The compounds selected (and the drug class) were estradiol (steroids), artemisinin (terpene lactones), rosiglitazone, pioglitazone, and troglitazone (glitazones), and repaglinide (meglitinides). For each drug, there were several major and minor metabolites formed, with human P450-derived metabolites produced for most compounds. All drugs apart from artemisinin displayed 100% conversion by LCMS/MS within 180 min. The metabolic profiles of several drugs were relatively complex, with many minor metabolites produced that proved difficult to elucidate and separate. Table 2 details the BM3 DM-derived metabolite(s) produced and related human P450 metabolizers. BM3 DM metabolites, in comparison to WT metabolites, are shown in Table S2. HPLC, LCMS/MS, and NMR spectra and structural elucidation information can be found in the Supporting Information (Figures S4–S40). The structures of the metabolites produced and their similarity to human metabolites are considered in the Discussion section.

**Table 2:**
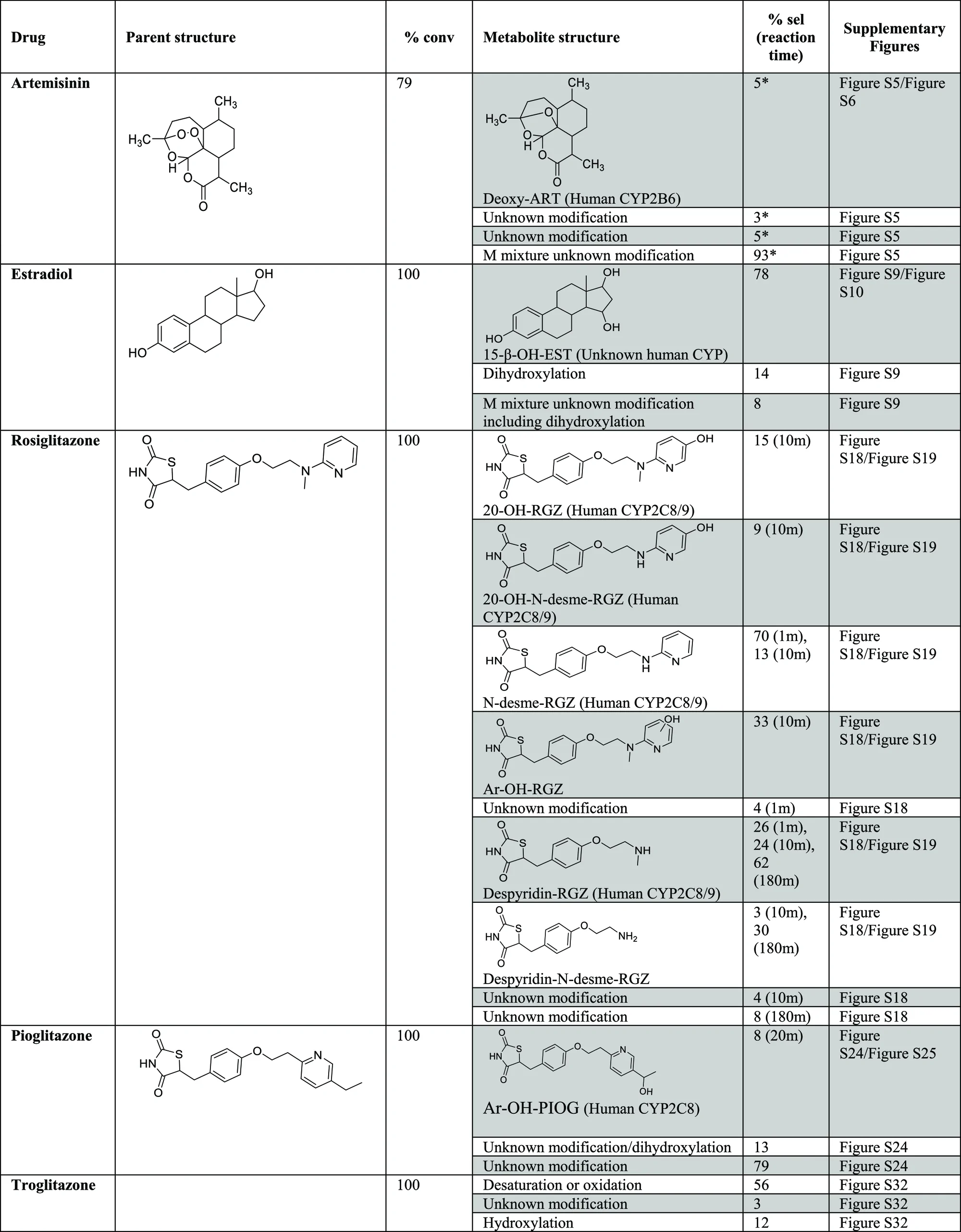

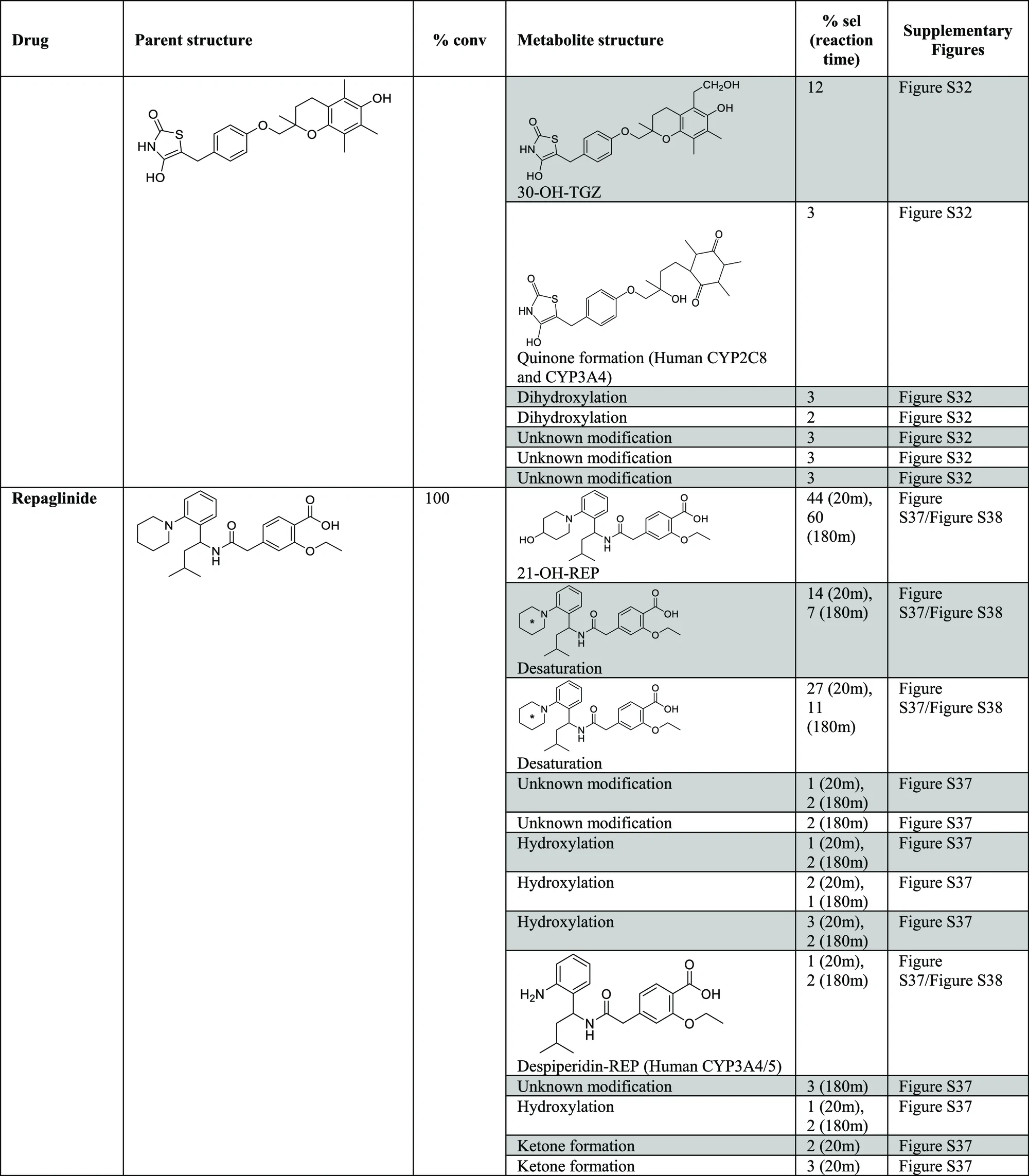
Summary Table of BM3 WT and DM Conversions and Metabolite Production of Structurally Diverse Pharmaceuticals^a^

a The percentage conversion (% conv) and selectivity (% sel) for BM3 WT and DM-derived metabolites (M) were calculated by determining the peak area ratio of the parent compound in the peak chromatogram in comparison to the culminative peak area of all compounds, and by determining the peak area ratio of the metabolite in the peak chromatogram in comparison to the culminative peak area of all metabolites, respectively. Fully structurally elucidated metabolite schematics are shown, with the related human P450 isoform stated in brackets if relevant. N/A indicates the metabolite was not produced by the BM3 enzyme. An asterisk (*) denotes the presence of several adducts in the MS led to selectivity ratios being approximated. For artemisinin, this resulted in a total of 106%, as the M mixture unknown modification is a mixed species, which also includes the 5% unknown modification. All MS and NMR data are shown in Supplementary Figures S4–S40. An expanded version of this table is available in the Supplementary Information (Table S2).

It was found that the time between LCMS and NMR experiments provided the opportunity for a number of compounds to convert to other metabolites. For example, we observed the conversion of the hydroxylated pioglitazone metabolite (“M-IV” in literature) during our MS experiments to the keto metabolite (“M-III” in literature) observed in MS and NMR experiments.^33^ Similarly, during the MS experiments, the methine metabolite of troglitazone is observed, but through a combination of isomerization and Michael addition, the quinone intermediate (″M3″ in literature) is the only metabolite observed in significant quantities for NMR assignment.^34,35^ The metabolites confirmed by NMR are shown in Figure 3. For some compounds, the high number of metabolites produced by BM3 DM caused great difficulty in the full identification of the structure of the metabolite. Repaglinide was particularly difficult; the 21-OH-repaglinide metabolite (sometimes called 3-OH-repaglinide due to different naming conventions) could be tentatively assigned using the standard in NMR (Figure S40), and by a combination of MSMS and NMR, a despiperidine metabolite was observed. Many of the masses observed during mass spectrometry could correspond to human metabolites. For artemisinin, estradiol, rosiglitazone, and troglitazone, many metabolites were identified, including several human metabolites. Estradiol is hydroxylated at seven different positions in humans by P450 enzymes;^36^ we observed two of these in our NMR experiments corresponding to 15- and 16-hydroxyestradiol. Rosiglitazone has a number of modifications in humans, including hydroxylations and demethylation.^37^ We observed the demethylation metabolite called “M12” in literature^37^ in our NMR experiments, though more human metabolites were observed in our MS experiments.

**Figure 3:**
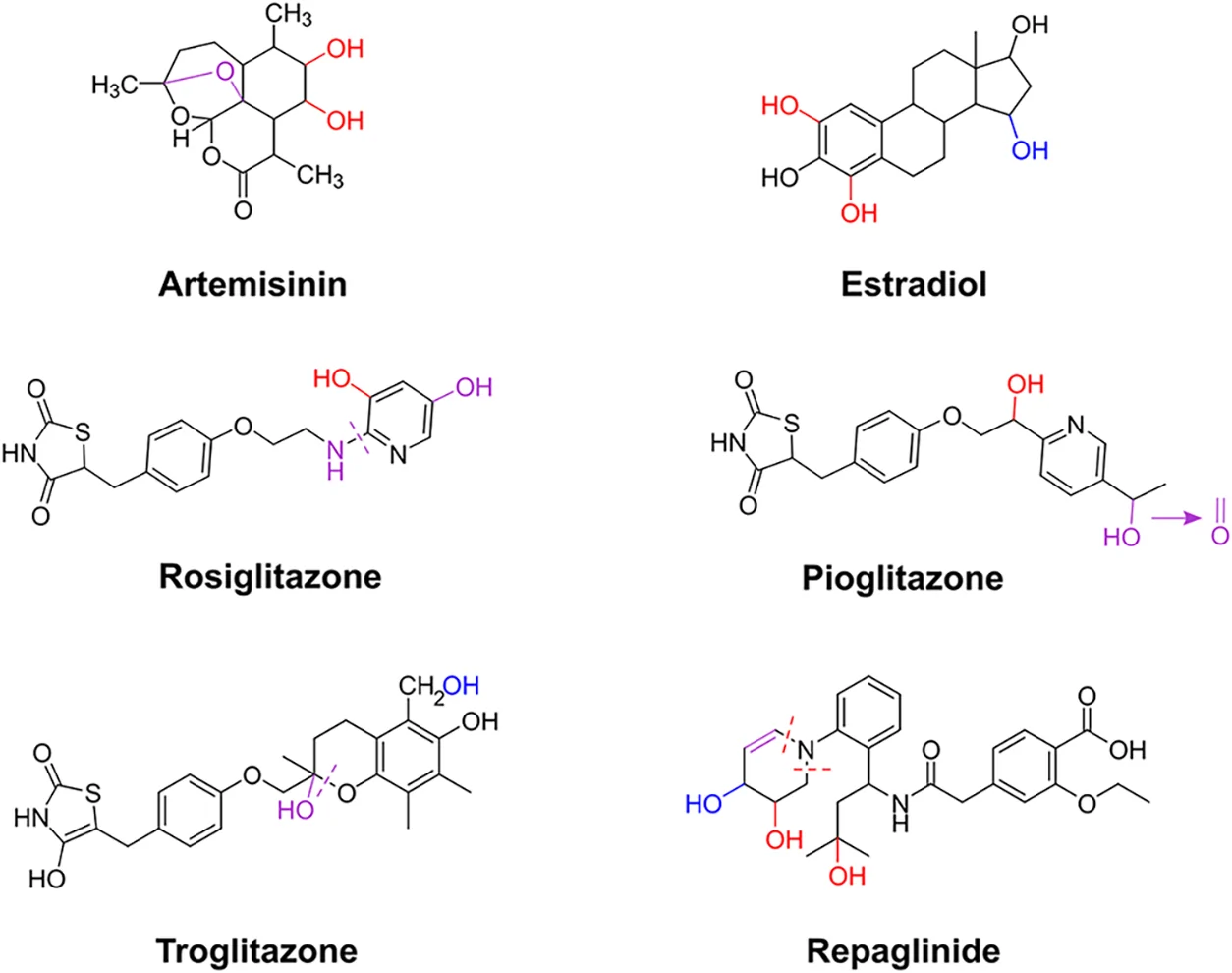
Pharmaceutical metabolism of compounds of interest. Modifications made by P450 enzymes are shown in red (human CYPs), blue (BM3 DM), and purple (human CYPs and BM3 DM). Bond breaks are denoted with a dashed line. The modifications of human CYP enzymes are taken from literature for artemisinin,^12^ estradiol,^31^ rosiglitazone,^32^ pioglitazone,^30^ troglitazone,^31,32^ and repaglinide.^33^ Only major modifications validated by NMR for BM3 DM are shown (full structural elucidation shown in Supplementary Figures S4–S40).

### EPR Spectroscopy of the DM Heme Domain Showed Substrate-Bound Characteristics at Low Temperatures

Focusing on the structurally diverse antidiabetic compounds, EPR spectra were collected for the BM3 DM heme domain (Table S3). In most cases, the binding of these compounds induced only minor shifts to the *g*-values in the EPR spectra and were similar to the spectra of the DMSO solvent control for the BM3 WT or DM-ligand-bound proteins. This is a common result, as the high-spin species (s = 5/2), indicative of substrate binding, is not favored at the low temperatures used in EPR. Despite this, the BM3 DM heme domain troglitazone-bound spectrum showed a substantially high-spin component (*g* = 7.94/3.64/1.70), suggesting that the tight-binding troglitazone substrate binds close to the heme iron, displacing the distal water and inducing the ligand-bound formation even at temperatures as low as 10 K (Figure S41).

### Co-Crystallization of the Troglitazone-Bound BM3 DM Heme Domain Showed Tight Interactions with the Heme Prosthetic Group

The BM3 DM heme domain in complex with troglitazone was successfully crystallized at 4 °C by the sitting drop method in the condition 0.2 M ammonium chloride and 20% w/v PEG 3350. The troglitazone-bound structure (PDB 6HN8) adopts the closed conformation attributed to substrate binding. Troglitazone makes very close H-bonding interactions with the residues Ala264, Thr268, and Tyr51 in the active site, and with the heme prosthetic group (at distances of 2.8, 3.1, 3.1, and 3.0 Å, respectively), as shown in Figure 4. The distance between the heme iron and the troglitazone molecule (3.2 Å) is surprisingly close for a typical substrate interaction and nearer to the 2.2 Å distance demonstrated by the inhibitor voriconazole,^24^ than to substrate distances, such as the 7.7 Å distance demonstrated by N-palmitoyl glycine or the 4.1 Å distance demonstrated by omeprazole.^23,24,38^ It should be noted that the troglitazone molecule shown is the 2R-5R enantiomer (PDB TDZ). The molecule binds with its trimethylated/hydroxylated aromatic ring closest to the heme prosthetic group. This ring is the location of modification during turnover, as observed in Table 2, although in the observed structure, the C30 oxidation is not accessible and the close distance between the heme iron and methyl would not permit oxygen binding, suggesting that this is possibly an equilibrium position stabilized in crystal formation and not the catalytic position for turnover. All crystallographic data are presented in Table S4.

**Figure 4:**
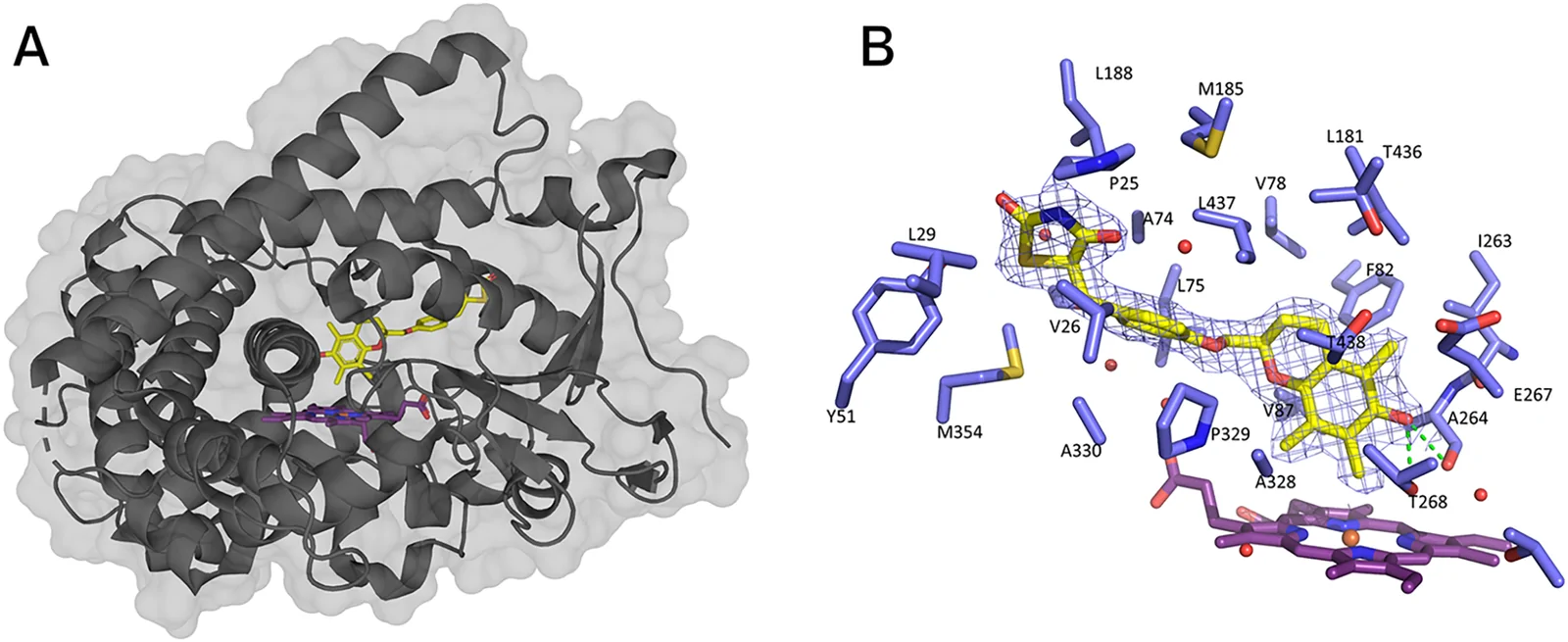
Troglitazone binding in the active site of the DM BM3 heme domain. Panel A shows the full heme domain in gray with its surface semitransparent. Troglitazone is shown in yellow, and the heme prosthetic group is shown in purple. Panel B shows how troglitazone (yellow) makes a number of close interactions with active site residues (lilac) and the heme prosthetic group (purple). The electron density for troglitazone is shown in blue.

## Discussion and Outlook

The binding screen showed that BM3 DM can bind over 53% of a structurally diverse 978 FDA compound library, eliciting a type I UV–visible spectral shift indicative of substrate binding. Additionally, 61 compounds (6.2% of the compounds screened) were found to elicit type II inhibitory shifts. The binding mode of such compounds is of interest, as this could lead to drug interactions, particularly when compounds interact with human P450 enzymes, such as CYP3A4, which is responsible for metabolizing ∼50% of pharmaceutical drugs.^39,40^ The compounds that elicited spectral shifts contained many different functional groups and displayed a wide range of molecular weights, highlighting the promiscuous and highly flexible nature of the A82F/F87V DM variant. Based on previous results indicating the formation of human P450-derived metabolites for proton pump inhibitor drugs by BM3 DM^22,22^ product determination for our compounds of interest was undertaken. BM3 DM successfully produced metabolites for all drugs tested, with 100% turnover by LCMS parent compound depletion reached within 3 h for all drugs apart from artemisinin. Rapid BM3 DM turnover of structurally diverse drug classes highlights its potential as a fast and efficient tool for P450-related metabolite production. The formation of human P450-derived metabolites for all drugs shows that BM3 DM has the potential to be used as an alternative to typically slower and expensive human P450 metabolite production. Significant metabolites that are dually produced by BM3 DM (Table 2) and human P450 isoforms are deoxy-artemisinin (CYP2B6),^41^ N-desmethyl-rosiglitazone (CYP2C8/9),^37,42^ keto-pioglitazone (CYP2C8),^33^ and despiperidinyl-repaglinide (CYP2A8 and CYP3A4).^43,44^ These structures are summarized in Figure 3. While there was a trend of selective aromatic/aliphatic hydroxylations for both major and minor metabolites of each drug, there was a wide range of biotransformations yielding several structurally diverse metabolites. The metabolites produced showed that BM3 DM is capable of targeting many different functional group sites and performing a range of biotransformations, including but not limited to group migrations on terpene lactones (deoxy-artemisinin); aliphatic N-demethylations (N-desmethyl-rosiglitazone); pyridine (despyridinil-rosiglitazone) and piperidine (N-despiperidinyl-repaglinide) ring cleavages and piperidine ring openings; aromatic hydroxylations (20-OH-rosiglitazone); stereoselective aliphatic ring hydroxylations (15-β–OH-estradiol and 28-ax–OH-repaglinide); ketone formation (keto-pioglitazone); and aliphatic dehydrogenation (repaglinide desaturation metabolites). The modifications typically occur on aliphatic chains/rings, which is in line with BM3′s natural substrate preference for fatty acids.^7^ However, modifications were also common on pyridine/piperidine/lactone rings, showing the expansion of BM3 DM’s catalytic repertoire and its ability to be an excellent tool for the completion of challenging reactions.

For the majority of drugs, there was typically one major BM3 DM metabolite produced, alongside several minor secondary metabolites. The lack of selectivity for one metabolite and the wide metabolic profile for each drug are potential caveats when utilizing BM3 DM for metabolite production. For instance, BM3 DM produces 13 different repaglinide metabolites that exhibit a wide variety of modification modes, of which many are not produced in high enough quantities for structural elucidation by NMR. The wide metabolic profile is most likely due to the active, open conformation of BM3 DM, leading to many binding modes for flexible drug structures such as aliphatic ring/chain motifs seen in repaglinide. For selectivity of one desired metabolite, further engineering would need to be conducted to avoid the need for extensive compound purification, such as the six mutations required for stereospecific hydroxylated testosterone products.^17^ However, this ambiguity allows the characterization of potential metabolites produced by a range of P450 enzymes, possibly allowing for the identification of toxic metabolites such as the quinone-type metabolite able to scavenge radicals produced in the metabolism of paracetamol^45^ or the troglitazone quinone metabolite produced here, thought to be responsible for liver toxicity leading to the removal of troglitazone from the market.^35^

The tight-binding observed by P450 BM3 with a number of antidiabetic compounds was of interest, particularly the glitazone drug class. Glitazones act by targeting PPAR-γ receptors to lower serum glucose and in doing so reverse the insulin resistance observed in type II diabetes.^46^ Pioglitazone is the best-selling glitazone drug and is widely prescribed, either in isolation or in tablet form containing other antidiabetic compounds, such as metformin. Yet other drugs within the drug class have failed during clinical trials or been withdrawn from the market. For example, within two years of being sold, troglitazone was removed from the market because of the death of 63 patients due to liver failure.^47^ This compound was found to produce a toxic quinone-type metabolite in humans.^35,48^ Metabolism resulting in toxic metabolites can cause reduced drug half-lives, side effects, and other problems, resulting in failed drug trials, loss of money and time, such as the loss of income due to troglitazone being removed from the market after only 2 years. The production of toxic metabolites such as this can be found during the characterization of human metabolites during preclinical trials using large commercial P450 screens. However, such screens have a number of limitations, including but not limited to long turnover times, short enzyme half-lives, the need for many P450 enzymes with specific mutations, amounts of compound needed, and, perhaps most importantly, expense.^2^ Recent advances in human P450 screens are slowly overcoming such limitations but also introduce new limitations such as path length issues, solvent binding, and the yield of protein.^49^ Interestingly, troglitazone causes many other issues such as drug interactions with paracetamol, leading to liver toxicity due to competition over CYP3A4 ^50^ and widespread immune responses due to the activation of inflammasomes in macrophages.^51^ Yet the structurally similar compound pioglitazone is a safe treatment for ameliorating hyperglycemia, adverse lipid metabolism, and blood pressure.^46^ Identification of metabolites leading to side effects or toxicity earlier in the drug discovery process would lead to many benefits for the pharmaceutical industry and consumers alike.

Many compounds induced substrate shifts in the UV–vis spectrum, however, troglitazone was the only substrate observed to produce a substantial shift in the *g*-values of the EPR spectrum indicative of ligand binding. P450 enzymes do not typically exhibit the ligand-bound conformation at the low temperatures required for heme EPR (10 K), as the ligand-free species is favored at low temperatures.^52,53^ The large substrate-bound signal from EPR suggested that troglitazone had an unusual binding mode for P450 BM3. Probing the binding mode further, X-ray crystallography was undertaken with the troglitazone-bound complex (PDB 6HN8). The ligand made a number of short-distance interactions with active site residues, in addition to its close approach to the heme prosthetic group. Comparing the troglitazone-bound BM3 DM heme domain structure to another BM3 DM structure (bound to the FDA-approved compound omeprazole, 4KEY), it is clear that the heme-troglitazone interaction distance is much shorter (approximately 3 Å and 4 Å, respectively).^22^ In both structures, the substrate interacts with Tyr51, a residue at the mouth of the active site associated with binding of the carboxylate group of fatty acid substrates.^54–56^ Troglitazone has two chiral centers (giving rise to four enantiomers), and yet only one enantiomer (PDB TDZ) is present in our structure (PDB 6HN8). This is in common with three other structures of diverse proteins: fatty acid binding protein 4 (PDB 2QM9), human carbonic anhydrase II (PDB 7M23), and CYP2C8DH (PDB 2VN0). In the CYP2C8 structure, troglitazone is described as a competitive inhibitor, as the binding mode of the molecule is more than 7.5 Å distant from the heme, suggesting this compound cannot interact with the heme prosthetic group but blocks other molecules from interacting with it. However, other studies have found that CYP2C8 is one of the P450 enzymes necessary for troglitazone metabolism, particularly for the formation of the toxic quinone metabolite.^57^ Interestingly, in our structure and the CYP2C8 structure, the troglitazone molecule binds with its trimethylated/hydroxylated aromatic ring closest to the heme prosthetic group.^34^ The low *K*_d_ value for troglitazone, high-spin EPR g-values, and its interactions observed within the crystal structure are consistent with this ligand binding at a short distance. While remaining a substrate interaction, this is nearer to distances expected for inhibitors, yet despite this, very tight-binding enzyme turnover was successful and products were observed. The distance of 3.2 Å between the troglitazone and the heme iron is particularly short, meaning the substrate is not in a catalytic position, as binding of oxygen would be precluded. It is expected that there are structural rearrangements during catalysis to allow oxygen binding, as have been demonstrated during early stages of catalysis with styrene as the substrate.^58^

In conclusion, this paper demonstrates how two “gatekeeper” mutations in the BM3 active site open up a plethora of diverse biotransformations for structurally diverse drug classes. BM3 DM has a promiscuity profile in the range of human P450 enzymes, with the advantage of easier and more cost-efficient metabolite production. The ability of BM3 DM to bind and metabolize structurally diverse drug classes while producing human P450 metabolites marks it as a valuable future tool in the production of human P450 metabolites or as part of biosynthetic routes to diversify compound libraries.

## Supplementary Material



## Notes

All spectral shifts (Table S1) and the characteristics associated with the compounds tested (Figures S1–S3), the metabolites generated for the WT and DM protein variants (Table S2), the EPR data (Table S3 and Figure S41), the crystallographic data (Table S4), and all LCMS and NMR data (Figures S4–S40) (PDF)
