# Supplementary material for: Expanding the Biocatalytic Toolbox: A Singular P450 Enzyme Efficiently Produces Diverse Pharmaceutical Metabolites
Source: ACS Omega. 2026 Aug 12;11(33):49151–62. doi: 10.1021/acsomega.6c01534 (PMC13520036; doi:10.1021/acsomega.6c01534)
Supplement: Supplementary file 1 [file ao6c01534_si_001.pdf]

Expanding the biocatalytic toolbox: a singular P450 enzyme efficiently produces diverse pharmaceutical metabolites

Laura N. Jeffreys [+*1], *Sian Thistlethwaite*[+1], *Matthew J. Cliff*[1], *Harshwardhan Poddar*[1], *Sahara Bhanot*[1], *Richard B. Tunnicliffe*[1], *Marina Golovanova*[1], *Katherine Hollywood*[1], *Colin W. Levy*[1], *Michael W. Voice*[2], *David Leys*[1], *Kirsty J. McLean*[3], *Jonathan P. Waltho*[*1 4], *Andrew W. Munro*[1] *and Hazel M. Girvan*[3]

AUTHOR INFORMATION

[+] - Jeffreys and Thistlethwaite contributed equally to this paper

* - Corresponding authors to whom correspondence should be addressed Laura.Jeffreys@manchester.ac.uk and J.Waltho@manchester.ac.uk

1 - Manchester Institute of Biotechnology, University of Manchester, Manchester M1 7DN, UK

2 - Cypex Ltd., 6 Tom McDonald Avenue, Dundee DD2 1NH, Scotland, United Kingdom

3 - Dept. of Physical and Life Sciences, School of Applied Sciences, University of Huddersfield, Queensgate, Huddersfield, HD1 3DH

4 – School of Biosciences, University of Sheffield, Firth Court, Western Bank, Sheffield, S10 2TN, UK

KEYWORDS

P450 BM3, antidiabetics, P450 metabolites, drug metabolites, type II diabetes, troglitazone, crystal structure, CYPs

RUNNING TITLE

A singular P450 efficiently produces diverse metabolites

SUPPLEMENTARY FIGURES

Table S1. Percentage spin-state shift values obtained under near-saturating ligand concentrations for the DM BM3 heme domain

For each substrate the percentage high-spin shift was calculated using a Python script and the percentage high spin equation, as described in the Methods section. The relevant compound/drug class is indicated in the final column. Type II (inhibitor-like) Soret red shifts are labelled as "inhibitors". N/A indicates a drug missing from the library received. NSB indicates that "no steady baseline" could be achieved due to solubility issues with the relevant ligands. PD indicates "protein depletion" as observed by heme dissociation from the DM heme domain, accompanied by protein aggregation/turbidity in many cases.

Drug	Molecular Weight	Indicator	%HS-DMSO
**1-Hexadecanol**	242.44	N/A	0.00%
**Acyclovir (Aciclovir)**	225.2	Infection	0.00%
**Allopurinol (Zyloprim)**	136.11	Neurological Disease	0.00%
**Amiloride hydrochloride dihydrate**	302.12	Cardiovascular Disease	0.00%
**Avanafil**	483.95	Cardiovascular Disease	0.00%
**Benzbromarone**	424.08	N/A	0.00%
**Betaxolol hydrochloride (Betoptic)**	343.89	Cardiovascular Disease	0.00%
**Bicalutamide (Casodex)**	430.37	Endocrinology	0.00%
**Cabazitaxel (Jevtana)**	835.93	Neurological Disease	0.00%
**Cephalexin (Cefalexin)**	347.39	Infection	0.00%
**Chlorpheniramine Maleate**	390.86	Neurological Disease	0.00%
**Chlorprothixene**	315.86	Neurological Disease	0.00%
**Cinchophen**	249.26	Immunology	0.00%
**Clemastine Fumarate**	459.96	Immunology	0.00%
**Clobetasol propionate**	466.97	Neurological Disease	0.00%
**Crizotinib (PF-02341066)**	450.34	Cancer	0.00%
**Daunorubicin HCl (Daunomycin HCl)**	563.98	Cancer	0.00%
**Diminazene Aceturate**	515.52	Vermifuge	0.00%
**Dipyridamole (Persantine)**	504.63	Cardiovascular Disease	0.00%
**Dopamine hydrochloride (Inotropin)**	189.64	Infection	0.00%
**Doripenem Hydrate**	438.52	Infection	0.00%
**Ethionamide**	166.24	Infection	0.00%

**Fenbendazole**	299.35	N/A	0.00%
**Flavoxate HCl**	427.92	Neurological Disease	0.00%
**Floxuridine**	246.19	Cancer	0.00%
**Gatifloxacin**	375.39	N/A	0.00%
**Gemcitabine (Gemzar)**	263.2	Metabolic Disease	0.00%
**Levobetaxolol HCl**	343.89	Cardiovascular Disease	0.00%
**Levofloxacin (Levaquin)**	361.37	Infection	0.00%
**Lonidamine**	321.16	Cardiovascular Disease	0.00%
**Lovastatin (Mevacor)**	404.54	Respiratory Disease	0.00%
**MDV3100 (Enzalutamide)**	464.44	Cancer	0.00%
**Mequinol**	124.14	Infection	0.00%
**Methimazole (Tapazole, Northyx)**	114.17	Endocrinology	0.00%
**Metolazone (Zaroxolyn)**	365.83	Cardiovascular Disease	0.00%
**Nadifloxacin**	360.38	Neurological Disease	0.00%
**Nifuroxazide**	275.22	Infection	0.00%
**Nitrendipine**	360.3699	Neurological Disease	0.00%
**Oxeladin Citrate**	527.6	N/A	0.00%
**Pralatrexate (Folotyn)**	477.47	Metabolic Disease	0.00%
**Ranitidine (Zantac)**	350.86	Metabolic Disease	0.00%
**Rifabutin (Mycobutin)**	847	Infection	0.00%
**Rimantadine (Flumadine)**	179.3	Infection	0.00%
**Ronidazole**	200.15	Neurological Disease	0.00%
**Sildenafil citrate**	666.7	Cardiovascular Disease	0.00%
**Sparfloxacin**	392.4	Infection	0.00%
**Sulfasalazine (Azulfidine)**	398.39	Inflammation	0.00%
**Sulfathiazole**	255.32	Infection	0.00%
**Sulindac (Clinoril)**	356.41	Cancer	0.00%
**Tigecycline**	585.65	Infection	0.00%
**tinidazole**	247.27	Infection	0.00%
**Vincristine sulfate**	923.04	Cancer	0.00%
**Zolmitriptan (Zomig)**	287.36	Neurological Disease	0.00%
**Tiotropium Bromide hydrate**	490.43	Infection	0.09%
**Trichlormethiazide (Achletin)**	380.66	Cardiovascular Disease	0.25%
**Difluprednate**	508.55	Endocrinology	0.27%
**Olmesartan medoxomil (Benicar)**	558.59	Cardiovascular Disease	0.27%
**Cyclosporine (Neoral)**	1202.61	Immunology	0.27%
**Dofetilide (Tikosyn)**	441.56	Cardiovascular Disease	0.29%

**Dextrose (D-glucose)**	180.16	Infection	0.40%
**Acipimox**	154.12	Cardiovascular Disease	0.46%
**Ketoprofen (Actron)**	254.28	Inflammation	0.54%
**Moxalactam Disodium**	564.44	Infection	0.58%
**Acetanilide (Antifebrin)**	135.16	Neurological Disease	0.60%
**Medroxyprogesterone acetate**	386.52	Infection	0.74%
**Phentolamine mesilate**	377.46	Cardiovascular Disease	0.75%
**Indomethacin (Indocid, Indocin)**	357.79	Inflammation	0.77%
**Nepafenac**	254.28	Inflammation	0.77%
**TAME**	342.41	Cancer	0.88%
**Vecuronium Bromide**	637.73	Neurological Disease	0.97%
**Trifluridine (Viroptic)**	296.2	Infection	1.00%
**Nithiamide**	187.18	N/A	1.09%
**Prednisolone (Hydroretrocortine)**	360.44	Infection	1.20%
**Doxylamine Succinate**	388.46	Neurological Disease	1.28%
**Ranolazine dihydrochloride**	500.46	Cardiovascular Disease	1.33%
**Cytidine**	243.22	Cardiovascular Disease	1.37%
**Adenine hydrochloride**	171.59	Cancer	1.39%
**Amfenac Sodium (monohydrate)**	295.27	Inflammation	1.43%
**Genistein**	270.24	Cancer	1.47%
**Rapamycin (Sirolimus)**	914.18	Immunology	1.47%
**Dutasteride**	528.53	Endocrinology	1.58%
**Etoposide (VP-16)**	588.56	Cancer	1.71%
**Probucol**	516.84	Cardiovascular Disease	1.87%
**Rivastigmine tartrate (Exelon)**	400.42	Cardiovascular Disease	1.87%
**Sitafloxacin Hydrate**	436.84	N/A	1.91%
**Fesoterodine fumarate (Toviaz)**	527.65	Immunology	1.95%
**Sotalol (Betapace)**	308.82	Neurological Disease	1.97%
**Tizanidine HCl**	290.17	Neurological Disease	2.00%
**Moclobemide**	268.74	Neurological Disease	2.03%
**Indapamide (Lozol)**	365.83	Cardiovascular Disease	2.07%
**Sulfanilamide**	172.2	Infection	2.16%
**Ethacridine lactate monohydrate**	361.39	Infection	2.16%
**Tylosin tartrate**	1066.19	Neurological Disease	2.20%
**Trimebutine**	387.47	Neurological Disease	2.21%
**Roxatidine acetate HCl**	384.9	Digestive system disease	2.26%
**Enoxacin (Penetrex)**	320.32	Infection	2.27%

**Paroxetine HCl**	365.83	Infection	2.33%
**Vidarabine (Vira-A)**	267.24	Infection	2.34%
**Enalaprilat dihydrate**	348.4	Cardiovascular Disease	2.45%
**Asenapine**	401.84	Neurological Disease	2.52%
**Raltegravir (MK-0518)**	444.42	Immunology	2.61%
**Propafenone (Rytmonorm)**	377.9	Cardiovascular Disease	2.63%
**Niacin (Nicotinic acid)**	123.11	Metabolic Disease	2.65%
**Sulfaguanidine**	214.24	Infection	2.71%
**Metoprolol tartrate**	684.81	Cardiovascular Disease	2.72%
**Cisatracurium besylate (Nimbex)**	1243.48	Neurological Disease	2.80%
**Mesalamine (Lialda)**	153.14	Inflammation	2.89%
**Oxytetracycline dihydrate**	496.46	Infection	2.95%
**Idarubicin HCl**	533.95	Cancer	2.99%
**Zaltoprofen**	298.36	Inflammation	3.01%
**Atracurium besylate**	1243.48	Neurological Disease	3.01%
**Almotriptan malate (Axert)**	469.55	Cardiovascular Disease	3.04%
**Rifapentine (Priftin)**	877.03	Infection	3.06%
**Solifenacin succinate**	480.55	Cardiovascular Disease	3.15%
**Anagrelide HCl**	292.55	Endocrinology	3.15%
**Diphenhydramine HCl (Benadryl)**	291.82	Immunology	3.16%
**Rifaximin (Xifaxan)**	785.88	Infection	3.20%
**Chlorpromazine (Sonazine)**	355.33	Neurological Disease	3.27%
**Epirubicin HCl**	579.98	N/A	3.32%
**Isoniazid (Tubizid)**	137.14	Infection	3.39%
**Telbivudine (Sebivo, Tyzeka)**	242.23	Infection	3.54%
**Prilocaine**	220.31	Neurological Disease	3.56%
**Meropenem**	383.46	Infection	3.59%
**Linezolid (Zyvox)**	337.35	Infection	3.60%
**Erythromycin (E-Mycin)**	733.93	Infection	3.77%
**Ranolazine (Ranexa)**	427.54	Cardiovascular Disease	3.79%
**Scopine**	155.19	Metabolic Disease	3.87%
**Flurbiprofen (Ansaid)**	244.26	Inflammation	3.89%
**Megestrol Acetate**	384.51	Infection	3.93%
**Isradipine (Dynacirc)**	371.39	Neurological Disease	4.01%
**Bortezomib (Velcade)**	384.24	Cancer	4.04%
**Dapoxetine hydrochloride (Priligy)**	341.87	Neurological Disease	4.07%
**Adefovir Dipivoxil (Preveon, Hepsera)**	501.47	Infection	4.10%

**Memantine HCl (Namenda)**	215.76	Neurological Disease	4.10%
**Apixaban**	459.5	Cardiovascular Disease	4.18%
**Lincomycin hydrochloride (Lincocin)**	443	Cancer	4.25%
**Ornidazole**	219.63	Endocrinology	4.25%
**Vitamin C (Ascorbic acid)**	176.12	Respiratory Disease	4.26%
**Sumatriptan succinate**	413.49	Neurological Disease	4.26%
**Clorsulon**	380.66	Cancer	4.33%
**Flutamide (Eulexin)**	276.21	Cancer	4.38%
**Triamcinolone (Aristocort)**	394.43	Inflammation	4.41%
**Mycophenolate mofetil (CellCept)**	433.49	Immunology	4.45%
**Adenosine (Adenocard)**	267.24	Cardiovascular Disease	4.57%
**Iloperidone (Fanapt)**	426.48	Neurological Disease	4.58%
**Pyridostigmine Bromide (Mestinon)**	261.12	Cardiovascular Disease	4.66%
**Geniposide**	388.37	N/A	4.67%
**Trimipramine Maleate**	410.51	N/A	4.79%
**Mirtazapine (Remeron, Avanza)**	265.35	Immunology	4.88%
**Prednisone (Adasone)**	358.43	Immunology	4.93%
**Silodosin (Rapaflo)**	495.53	Cardiovascular Disease	4.93%
**Argatroban**	508.63	Cardiovascular Disease	5.00%
**OSI-420 (Desmethyl Erlotinib)**	415.87	Cancer	5.05%
**Cyproheptadine HCl (Periactin)**	323.86	Neurological Disease	5.08%
**Imidapril (Tanatril) HCl**	441.91	Cardiovascular Disease	5.09%
**Fluoxetine HCl**	345.79	Neurological Disease	5.17%
**Axitinib**	386.47	Cancer	5.23%
**Candesartan (Atacand)**	440.45	Cardiovascular Disease	5.24%
**Ivacaftor (VX-770)**	392.49	Respiratory disease	5.25%
**Fenoprofen calcium hydrate**	558.63	Immunology	5.30%
**Vinblastine sulfate**	909.05	Neurological Disease	5.30%
**Ethambutol HCl**	277.23	Neurological Disease	5.49%
**Diltiazem HCl (Tiazac)**	450.98	Cardiovascular Disease	5.61%
**Diclofenac Potassium**	334.24	Infection	5.64%
**Altretamine (Hexalen)**	210.28	Cancer	5.65%
**Amantadine hydrochloride (Symmetrel)**	187.7	Cardiovascular Disease	5.68%
**Tenofovir**	287.21	N/A	5.68%
**Resveratrol**	228.24	Infection	5.70%

**Lidocaine (Alphacaine)**	234.34	Neurological Disease	5.71%
**Niflumic acid**	282.22	Infection	5.84%
**Melatonin**	232.28	Endocrinology	5.85%
**Pergolide mesylate**	410.59	Neurological Disease	5.87%
**Arecoline**	236.11	Endocrinology	5.88%
**Tebipenem pivoxil (L-084)**	497.63	Cardiovascular Disease	5.90%
**Xylose**	150.13	Metabolic Disease	5.96%
**Rivaroxaban (Xarelto)**	435.88	Metabolic Disease	5.99%
**Primidone (Mysoline)**	218.25	Neurological Disease	6.01%
**Thiamphenicol (Thiophenicol)**	356.22	Cardiovascular Disease	6.05%
**Risperidone (Risperdal)**	410.48	Neurological Disease	6.06%
**Adiphenine HCl**	347.88	Cardiovascular Disease	6.07%
**10-Deacetylbaccatin-III**	544.59	N/A	6.23%
**Epinephrine bitartrate (Adrenalinium)**	333.29	Cancer	6.30%
**Flunarizine 2HCl**	477.42	Cancer	6.40%
**Pitavastatin calcium (Livalo)**	880.98	Cardiovascular Disease	6.44%
**Uridine**	244.2	Vermifuge	6.48%
**Tolterodine tartrate (Detrol LA)**	475.57	Neurological Disease	6.48%
**Nitrofurazone (Nitrofural)**	198.14	Infection	6.50%
**Mevastatin**	390.51	Cardiovascular Disease	6.55%
**Broxyquinoline**	302.95	Vermifuge	6.58%
**Ketotifen fumarate (Zaditor)**	425.5	Neurological Disease	6.59%
**Etodolac (Lodine)**	287.35	Inflammation	6.62%
**Pefloxacin mesylate**	429.46	Infection	6.70%
**Nifedipine (Adalat)**	346.33	Cardiovascular Disease	6.73%
**Methscopolamine (Pamine)**	398.29	Neurological Disease	6.74%
**Stavudine**	224.21	Infection	6.81%
**Chlormezanone (Trancopal)**	273.74	Respiratory Disease	6.94%
**Formoterol hemifumarate**	402.4	Neurological Disease	6.94%
**Temocapril HCl**	513.07	Cancer	6.97%
**Prochlorperazine Dimaleate**	606.09	N/A	7.00%
**Rofecoxib (Vioxx)**	314.36	Digestive system disease	7.08%
**Ritodrine hydrochloride (Yutopar)**	323.81	Infection	7.08%
**Moroxydine**	207.66	Cancer	7.10%
**Sulphadimethoxine**	310.33	Infection	7.15%

**Teniposide (Vumon)**	656.65	Cancer	7.27%
**Clindamycin hydrochloride (Dalacin)**	461.44	Neurological Disease	7.35%
**Reboxetine mesylate**	409.5	Neurological Disease	7.43%
**Pyrazinamide (Pyrazinoic acid amide)**	123.11	Infection	7.44%
**Quetiapine fumarate (Seroquel)**	883.09	Neurological Disease	7.44%
**Famotidine (Pepcid)**	337.45	Cardiovascular Disease	7.45%
**Tadalafil (Cialis)**	389.4	Cardiovascular Disease	7.54%
**Methyclothiazide**	360.24	Cardiovascular Disease	7.59%
**Vinorelbine Tartrate**	1079.11	N/A	7.68%
**Enalapril maleate (Vasotec)**	492.52	Cardiovascular Disease	7.70%
**Propranolol HCl**	295.8	Cardiovascular Disease	7.76%
**Oxytetracycline (Terramycin)**	460.43	Infection	7.83%
**Chlorothiazide**	295.72	Cardiovascular Disease	7.98%
**Flumequine**	261.25	Metabolic Disease	8.09%
**Nalmefene HCl**	375.89	Neurological Disease	8.11%
**Lafutidine**	431.55	Infection	8.12%
**Pancuronium (Pavulon)**	732.67	Cardiovascular Disease	8.13%
**Bleomycin sulfate**	1512.62	Cancer	8.16%
**Maraviroc**	513.67	Inflammation	8.26%
**Pazopanib HCl**	473.98	Cancer	8.28%
**Emtricitabine (Emtriva)**	247.25	Infection	8.29%
**Diclofenac**	318.13	Neurological Disease	8.33%
**Nialamide**	298.34	Neurological Disease	8.34%
**Phenacetin**	179.22	Infection	8.38%
**Imipramine HCl**	316.87	N/A	8.38%
**Brucine**	510.56	N/A	8.45%
**Busulfan (Myleran, Busulfex)**	246.3	Cardiovascular Disease	8.49%
**Furaltadone HCl**	360.75	Infection	8.50%
**Flucytosine (Ancobon)**	129.09	Infection	8.55%
**Nabumetone**	228.29	Inflammation	8.58%
**Cilostazol**	369.46	Cardiovascular Disease	8.65%
**Chloramphenicol (Chloromycetin)**	323.13	Infection	8.66%
**Tropisetron**	320.81	Neurological Disease	8.67%
**Divalproex sodium**	310.41	Neurological Disease	8.73%

**Diclofenac Diethylamine**	369.29	Neurological Disease	8.74%
**Mecarbinate**	233.26	Metabolic Disease	8.74%
**valganciclovir hydrochloride**	390.82	Endocrinology	8.74%
**Fluorometholone Acetate**	418.5	Inflammation	8.78%
**Oxcarbazepine**	252.27	Neurological Disease	8.81%
**Azelastine hydrochloride (Astelin)**	418.36	Neurological Disease	8.84%
**Methacycline hydrochloride (Physiomycine)**	478.88	Cancer	8.86%
**Betamethasone (Celestone)**	392.46	Inflammation	8.94%
**Phenindione (Rectadione)**	222.24	Cardiovascular Disease	8.96%
**Methazolamide**	236.27	Neurological Disease	9.01%
**Maprotiline hydrochloride**	313.86	Neurological Disease	9.01%
**Articaine HCl**	320.84	Neurological Disease	9.06%
**Betaxolol (Betoptic)**	307.43	Neurological Disease	9.08%
**Suplatast tosylate**	499.64	Cardiovascular Disease	9.08%
**Genipin**	226.23	N/A	9.08%
**Procaine (Novocaine) HCl**	272.77	Neurological Disease	9.19%
**Fenoprofen calcium**	522.6	Inflammation	9.20%
**Gefitinib (Iressa)**	446.9	Cancer	9.27%
**Isoxicam**	335.34	N/A	9.29%
**Nalidixic acid (NegGram)**	232.24	Infection	9.30%
**Meglumine**	195.21	N/A	9.33%
**Mianserin hydrochloride**	300.83	Neurological Disease	9.35%
**Ambrisentan**	378.42	Neurological Disease	9.51%
**Praziquantel (Biltricide)**	312.41	Vermifuge	9.52%
**Chlorpropamide**	276.74	Infection	9.63%
**Telmisartan (Micardis)**	514.62	Cardiovascular Disease	9.65%
**Pramoxine HCl**	329.86	Neurological Disease	9.70%
**Darunavir Ethanolate (Prezista)**	593.73	Infection	9.71%
**Dimethyl Fumarate**	144.13	Inflammation	9.73%
**Mometasone furoate**	521.43	Inflammation	9.74%
**Azithromycin (Zithromax)**	748.98	Cancer	9.81%
**Pranoprofen**	255.27	Inflammation	9.91%
**Ivermectin**	875.09	Vermifuge	10.00%
**Amoxicillin sodium (Amox)**	387.39	Infection	10.08%
**Proadifen HCl**	390.01	N/A	10.09%
**Sulfisoxazole**	267.3	Infection	10.11%

**Clorprenaline HCL**	250.16	Cardiovascular Disease	10.13%
**Doxapram HCl**	432.98	Neurological Disease	10.19%
**Doxorubicin (Adriamycin)**	579.98	Cancer	10.22%
**Bendamustine HCL**	394.72	Cancer	10.26%
**Zalcitabine**	211.22	Infection	10.27%
**Menadione**	172.18	Endocrinology	10.28%
**Desonide**	416.51	Inflammation	10.29%
**Nafamostat mesylate**	539.58	Cardiovascular Disease	10.33%
**Venlafaxine**	313.86	Neurological Disease	10.36%
**Noscapine HCl**	449.88	N/A	10.39%
**Cilnidipine**	492.52	Cardiovascular Disease	10.41%
**Dyclonine HCl**	325.87	Inflammation	10.44%
**Guanabenz acetate**	291.13	Endocrinology	10.46%
**Abitrexate (Methotrexate)**	454.44	Cancer	10.50%
**Evista (Raloxifene Hydrochloride)**	510.04	Endocrinology	10.50%
**Pidotimod**	244.27	Immunology	10.66%
**Anisotropine Methylbromide**	362.35	Neurological Disease	10.66%
**Bepotastine Besilate**	547.06	Cancer	10.70%
**Acetylcholine chloride**	181.66	Neurological Disease	10.73%
**Potassium iodide**	166	Endocrinology	10.81%
**Trospium chloride (Sanctura)**	427.96	Cardiovascular Disease	10.83%
**Ramipril (Altace)**	416.51	Cardiovascular Disease	10.88%
**Acarbose**	645.6	Metabolic Disease	10.90%
**Ibuprofen (Advil)**	206.28	Inflammation	10.94%
**Aztreonam (Azactam, Cayston)**	435.43	Infection	10.94%
**Dyphylline (Dilor)**	254.24	Respiratory Disease	10.94%
**Spironolactone**	416.57	Infection	10.99%
**Methylprednisolone**	374.47	Immunology	11.00%
**sulfacetamide sodium**	236.22	Cardiovascular Disease	11.08%
**Pentamidine**	413.34	Infection	11.10%
**Phenylephrine HCl**	203.67	Endocrinology	11.10%
**Clomipramine HCl**	351.31	N/A	11.13%
**Sulfamerazine**	264.3	Infection	11.14%
**Amitriptyline HCl**	313.86	Infection	11.22%
**Valnemulin HCl**	601.28	Infection	11.22%
**Ondansetron hydrochloride (Zofran)**	329.82	Infection	11.22%
**Hydrochlorothiazide**	297.74	Cardiovascular Disease	11.23%
**Thiabendazole**	201.25	Vermifuge	11.25%
**Terazosin HCl (Hytrin)**	459.92	Infection	11.31%
**Dasatinib (BMS-354825)**	488.01	Cancer	11.33%

**Naproxen (Aleve)**	252.24	Inflammation	11.37%
**Sasapyrine**	258.23	Inflammation	11.39%
**Scopolamine hydrobromide**	384.26	Respiratory Disease	11.40%
**Cefoperazone (Cefobid)**	645.67	Infection	11.41%
**Pyrimethamine**	248.71	Immunology	11.45%
**Carbamazepine (Carbatrol)**	236.27	Neurological Disease	11.46%
**Hydrocortisone (Cortisol)**	362.46	Infection	11.46%
**Oxaliplatin (Eloxatin)**	397.29	Cancer	11.48%
**Pizotifen malate**	429.53	Inflammation	11.49%
**Nicotinamide (Niacinamide)**	122.12	Neurological Disease	11.51%
**Dibucaine HCL**	379.92	Endocrinology	11.51%
**Phenformin hydrochloride**	241.72	Metabolic Disease	11.51%
**Hydroxyzine 2HCl**	447.83	Neurological Disease	11.51%
**Triflupromazine HCl**	388.88	N/A	11.51%
**Disopyramide Phosphate**	437.47	Cardiovascular Disease	11.52%
**tetrahydrozoline hydrochloride**	236.74	Inflammation	11.61%
**Cepharanthine**	606.71	Metabolic Disease	11.61%
**Azacyclonol**	267.37	Neurological Disease	11.62%
**Rocuronium bromide**	609.68	Neurological Disease	11.69%
**Bisoprolol fumarate**	441.52	N/A	11.69%
**Edaravone (MCI-186)**	174.2	Cardiovascular Disease	11.80%
**Deferasirox (Exjade)**	373.36	Endocrinology	11.84%
**Amfebutamone (Bupropion)**	276.2	Infection	11.87%
**Cyclophosphamide monohydrate**	279.1	Cancer	11.89%
**Noradrenaline bitartrate monohydrate (Levophed)**	337.28	Metabolic Disease	11.89%
**Tiopronin (Thiola)**	163.19	Cardiovascular Disease	11.89%
**Naltrexone HCl**	377.86	Neurological Disease	11.91%
**Ethamsylate**	263.31	Cardiovascular Disease	11.93%
**Flunixin meglumin**	491.46	Immunology	12.01%
**Roxithromycin (Roxl-150)**	837.0673	Metabolic Disease	12.03%
**Trometamol**	121.14	N/A	12.03%
**Nelarabine (Arranon)**	297.27	Cancer	12.04%
**Lenalidomide**	259.26	Cardiovascular Disease	12.06%
**Nefiracetam (Translon)**	246.3	Neurological Disease	12.10%
**Vardenafil (Vivanza)**	579.11	Infection	12.12%
**Fluocinolone acetonide (Flucort-N)**	452.4999	Infection	12.13%

**Atazanavir sulfate**	802.93	Cancer	12.13%
**Urapidil HCl**	423.94	Respiratory Disease	12.17%
**Sertraline HCl**	342.69	Inflammation	12.17%
**Albendazole Oxide (Ricobendazole)**	281.33	Infection	12.22%
**Didanosine (Videx)**	236.23	Infection	12.22%
**Cladribine**	285.69	Cancer	12.26%
**Clofarabine**	303.68	Cancer	12.30%
**Thioridazine HCl**	407.04	N/A	12.31%
**Carvedilol**	406.47	Cardiovascular Disease	12.36%
**Brinzolamide**	383.51	Neurological Disease	12.36%
**Valsartan (Diovan)**	435.52	Cardiovascular Disease	12.39%
**Brompheniramine**	435.31	Infection	12.41%
**Methocarbamol (Robaxin)**	241.24	Neurological Disease	12.46%
**Lornoxicam (Xefo)**	371.82	Inflammation	12.50%
**Suprofen (Profenal)**	260.31	Inflammation	12.51%
**Lamivudine (Epivir)**	229.26	Infection	12.61%
**Dexamethasone**	392.46	Inflammation	12.63%
**Azatadine dimaleate**	522.55	Infection	12.63%
**Atomoxetine HCl**	291.82	Neurological Disease	12.64%
**Alibendol**	251.28	Neurological Disease	12.68%
**Clarithromycin (Biaxin, Klacid)**	747.95	Neurological Disease	12.69%
**Glipizide (Glucotrol)**	445.54	Endocrinology	12.69%
**Cimetidine (Tagamet)**	252.34	Inflammation	12.75%
**Rebamipide**	370.79	Infection	12.86%
**Clozapine (Clozaril)**	326.82	Cardiovascular Disease	12.87%
**Celecoxib**	381.37	Inflammation	12.98%
**Trifluoperazine 2HCl**	480.42	Neurological Disease	13.00%
**Antazoline HCl**	301.81	Neurological Disease	13.00%
**Amisulpride**	369.48	Neurological Disease	13.01%
**Pyrilamine Maleate**	401.46	N/A	13.05%
**Erythromycin Ethylsuccinate**	862.05	Infection	13.19%
**Ivabradine HCl (Procoralan)**	505.05	Neurological Disease	13.25%
**Zanamivir**	332.31	N/A	13.26%
**Antipyrine**	188.23	Infection	13.27%
**XL-184 (Cabozantinib)**	501.51	Cancer	13.28%
**Aminophylline (Truphylline)**	420.43	Respiratory Disease	13.30%
**Aceclidine HCl**	205.68	N/A	13.42%
**Bimatoprost**	415.57	Cardiovascular Disease	13.46%

**Mexiletine HCl**	215.72	Cardiovascular Disease	13.49%
**Balofloxacin**	389.42	Metabolic Disease	13.50%
**Dropropizine**	236.31	Respiratory Disease	13.51%
**Diclazuril**	407.64	Infection	13.53%
**Ciclopirox ethanolamine**	268.35	Infection	13.53%
**Pramipexole (Mirapex)**	211.33	Neurological Disease	13.55%
**Orlistat (Alli, Xenical)**	495.73	Metabolic Disease	13.56%
**Atropine sulfate monohydrate**	694.83	Respiratory Disease	13.58%
**Nilotinib (AMN-107)**	529.52	Cancer	13.59%
**Glyburide (Diabeta)**	494	Endocrinology	13.60%
**Desloratadine**	310.82	Cardiovascular Disease	13.61%
**Benzydamine Hydrochloride**	345.87	Inflammation	13.62%
**2-Thiouracil**	128.15	Endocrinology	13.63%
**Hydroxyurea (Cytodrox)**	76.05	Cancer	13.65%
**Tetracycline HCl**	480.9	Infection	13.68%
**Thalidomide**	258.23	Immunology	13.71%
**Voglibose**	267.28	Metabolic Disease	13.74%
**Hyoscyamine (Daturine)**	289.37	Neurological Disease	13.75%
**Pramiracetam**	269.38	Endocrinology	13.89%
**Tacrine HCl**	234.72	N/A	13.96%
**Candesartan cilexetil (Atacand)**	610.66	Cardiovascular Disease	14.06%
**Topiramate**	339.36	Neurological Disease	14.08%
**Proparacaine HCl**	330.85	Neurological Disease	14.11%
**5-Aminolevulinic acid hydrochloride**	167.59	Neurological Disease	14.13%
**Sulfameter (Bayrena)**	280.3	Infection	14.14%
**Terbinafine (Lamisil, Terbinex)**	291.43	Infection	14.14%
**Vorinostat (SAHA)**	264.3	Cancer	14.19%
**Erdosteine**	249.31	Respiratory Disease	14.19%
**Azaguanine-8**	152.11	Cancer	14.21%
**Zileuton**	236.29	Respiratory Disease	14.32%
**Aminosalicylate sodium**	211.15	Neurological Disease	14.34%
**Zidovudine (Retrovir)**	267.24	Cardiovascular Disease	14.37%
**Reserpine**	608.68	Cardiovascular Disease	14.41%
**Fludarabine Phosphate (Fludara)**	365.21	Cancer	14.48%
**Benztropine mesylate**	403.53	Infection	14.50%

**D-Mannitol (Osmitrol)**	182.17	Cardiovascular Disease	14.54%
**Monobenzone (Benoquin)**	200.23	Metabolic Disease	14.55%
**Spiramycin**	843.058	Infection	14.56%
**Furosemide (Lasix)**	330.74	Cardiovascular Disease	14.57%
**Mosapride citrate**	614.02	Neurological Disease	14.58%
**Flumazenil**	303.29	Neurological Disease	14.61%
**Trimethoprim**	290.32	Infection	14.62%
**Mupirocin**	500.62	N/A	14.63%
**Amprolium HCl**	315.24	Metabolic Disease	14.65%
**Alfuzosin hydrochloride (Uroxatral)**	425.91	Cardiovascular Disease	14.65%
**Acemetacin (Emflex)**	415.82	Infection	14.66%
**Dirithromycin**	835.07	Infection	14.70%
**Agomelatine**	243.3	N/A	14.74%
**Gabexate mesylate**	417.48	Cardiovascular Disease	14.75%
**Mirabegron (YM178)**	396.51	Cancer	14.79%
**Sodium 4-aminohippurate Hydrate**	216.17	N/A	14.80%
**Everolimus (RAD001)**	958.22	Cancer	14.80%
**Guaifenesin (Guaiphenesin)**	198.22	Respiratory Disease	14.81%
**Phthalylsulfacetamide**	362.36	N/A	14.84%
**Dicyclomine HCl**	345.95	N/A	14.87%
**Ouabain**	728.77	Neurological Disease	14.88%
**Orphenadrine citrate (Norflex)**	461.5	Infection	14.90%
**Salicylanilide**	213.23	Infection	14.97%
**Buflomedil HCl**	343.85	Neurological Disease	15.00%
**Nevirapine (Viramune)**	266.3	Infection	15.06%
**Uracil**	112.09	N/A	15.06%
**Novobiocin sodium (Albamycin)**	634.61	Cardiovascular Disease	15.08%
**Vitamin B12**	1355.37	Metabolic Disease	15.09%
**Cetirizine Dihydrochloride**	461.81	Inflammation	15.14%
**Diperodon HCl**	433.93	N/A	15.26%
**Aprepitant (MK-0869)**	534.43	Neurological Disease	15.32%
**Esmolol HCl**	331.83	Cardiovascular Disease	15.33%
**Moguisteine**	339.41	Respiratory Disease	15.34%
**Methylthiouracil**	142.18	Infection	15.34%
**Cysteamine HCl**	113.61	Metabolic Disease	15.35%

**Diphemanil methylsulfate**	389.51	Neurological Disease	15.39%
**Miglitol (Glyset)**	207.22	Neurological Disease	15.39%
**Nebivolol (Bystolic)**	441.9	Cardiovascular Disease	15.39%
**Levetiracetam**	170.21	Neurological Disease	15.42%
**Ponatinib (AP24534)**	532.56	Cancer	15.50%
**Ropinirole HCl**	296.84	Neurological Disease	15.53%
**Mepenzolate Bromide**	205.68	N/A	15.56%
**Nateglinide (Starlix)**	317.42	Immunology	15.62%
**Haloperidol (Haldol)**	375.86	Neurological Disease	15.62%
**Propylthiouracil**	170.23	Endocrinology	15.75%
**Loxapine Succinate**	445.9	Neurological Disease	15.81%
**Aniracetam**	219.24	Neurological Disease	15.86%
**Simvastatin (Zocor)**	418.57	Cardiovascular Disease	15.90%
**Bosentan**	551.61	N/A	15.93%
**Triamcinolone Acetonide**	434.5	Inflammation	15.99%
**Aminocaproic acid (Amicar)**	131.17	Cardiovascular Disease	16.03%
**Procyclidine HCl**	323.9	N/A	16.03%
**Riluzole (Rilutek)**	234.2	Neurological Disease	16.06%
**Ceftiofur HCl**	560.02	N/A	16.08%
**Isoprenaline hydrochloride**	247.72	Infection	16.09%
**Ruxolitinib (INCB018424)**	306.37	Cancer	16.17%
**Sodium salicylate**	161.11	Infection	16.24%
**Finasteride**	372.54	Endocrinology	16.27%
**Ibutilide fumarate**	885.23	Cardiovascular Disease	16.30%
**Flumethasone**	410.45	Endocrinology	16.32%
**Rufinamide (Banzel)**	238.19	Neurological Disease	16.36%
**Tolbutamide**	270.35	Cancer	16.36%
**Olanzapine (Zyprexa)**	312.4398	Infection	16.38%
**Disulfiram (Antabuse)**	296.54	Neurological Disease	16.41%
**Amiloride hydrochloride (Midamor)**	266.09	Metabolic Disease	16.42%
**Rasagiline mesylate**	267.34	Cardiovascular Disease	16.43%
**Metaproterenol Sulfate**	520.59	Respiratory Disease	16.43%
**Captopril (Capoten)**	217.29	Metabolic Disease	16.46%
**Clindamycin**	424.98	Infection	16.48%
**Cisplatin**	300.05	N/A	16.48%

**Meticrane**	275.34	Cardiovascular Disease	16.53%
**Pomalidomide**	273.24	Cancer	16.54%
**Telaprevir (VX-950)**	679.85	Infection	16.66%
**Deflazacort (Calcort)**	441.52	Endocrinology	16.67%
**Methenamine (Mandelamine)**	140.19	Inflammation	16.67%
**Famprofazone**	377.52	Inflammation	16.70%
**Procodazole**	190.2	N/A	16.72%
**Carfilzomib (PR-171)**	719.91	Cardiovascular Disease	16.72%
**Spectinomycin hydrochloride**	405.27	Cardiovascular Disease	16.76%
**Bindarit**	324.37	Cancer	16.78%
**Zonisamide**	212.23	Neurological Disease	16.87%
**Piromidic Acid**	288.32	N/A	16.87%
**Acetarsone**	275.09	N/A	16.96%
**Estriol**	288.39	Neurological Disease	17.02%
**Rosuvastatin calcium (Crestor)**	500.57	Infection	17.03%
**Pramipexole dihydrochloride monohydrate**	302.26	Neurological Disease	17.03%
**Sulfamethoxazole**	253.28	Infection	17.04%
**Dienogest**	311.42	Endocrinology	17.13%
**Valproic acid sodium salt (Sodium valproate)**	166.19	Cardiovascular Disease	17.13%
**Homatropine Bromide**	356.25	Infection	17.14%
**Temsirolimus (Torisel)**	1030.29	Cancer	17.15%
**Irsogladine**	256.09	Neurological Disease	17.20%
**Tenofovir Disoproxil Fumarate**	635.51	N/A	17.29%
**Mycophenolic (Mycophenolate)**	320.34	Infection	17.30%
**Docetaxel (Taxotere)**	807.88	Cancer	17.35%
**Pridinol Methanesulfonate**	391.52	N/A	17.38%
**Oxybuprocaine HCl**	344.88	Neurological Disease	17.40%
**Lamotrigine**	256.09	Cancer	17.44%
**Ftorafur**	200.17	Cancer	17.51%
**Phenylbutazone (Butazolidin, Butatron)**	308.37	Cancer	17.63%
**Chloroxine**	214.05	Infection	17.68%
**Deoxycorticosterone acetate**	372.5	Endocrinology	17.71%
**Benzthiazide**	431.94	Cardiovascular Disease	17.71%
**Gimeracil**	145.54	Neurological Disease	17.73%
**Bufexamac**	223.27	Metabolic Disease	17.75%
**Naloxone HCl**	363.84	Cancer	17.77%
**Bumetanide**	364.42	Cardiovascular Disease	17.78%
**Ginkgolide A**	408.4	Cardiovascular Disease	17.79%

**Aspartame**	294.3	Metabolic Disease	17.80%
**Nizatidine**	331.46	Metabolic Disease	17.82%
**Bupivacaine hydrochloride (Marcain)**	324.89	Neurological Disease	17.87%
**Dexrazoxane Hydrochloride**	304.73	Cardiovascular Disease	17.90%
**Mifepristone (Mifeprex)**	429.59	Metabolic Disease	17.92%
**Loperamide hydrochloride**	513.5	Infection	17.92%
**Ampiroxicam**	447.46	Cardiovascular Disease	17.92%
**Cyromazine**	166.18	Vermifuge	18.00%
**Cinepazide maleate**	533.57	Inflammation	18.04%
**Tolperisone HCl**	281.82	Neurological Disease	18.09%
**Amprenavir (Agenerase)**	505.63	Infection	18.13%
**Metaraminol Bitartrate**	317.29	N/A	18.16%
**Tioxolone**	168.17	Endocrinology	18.19%
**Diphenylpyraline HCl**	317.85	Neurological Disease	18.23%
**Serotonin HCl**	212.68	Neurological Disease	18.27%
**(+,-)-Octopamine HCl**	189.64	Immunology	18.30%
**Dydrogesterone**	312.45	Endocrinology	18.32%
**Arbidol HCl**	513.88	Cardiovascular Disease	18.37%
**Sulbactam sodium (Unasyn)**	255.22	Infection	18.39%
**Coumarin**	146.14	N/A	18.44%
**Rotigotine**	315.47	N/A	18.54%
**Rizatriptan Benzoate**	391.47	N/A	18.74%
**Ractopamine HCl**	337.84	N/A	18.75%
**Carbidopa**	226.23	Neurological Disease	18.80%
**Vildagliptin (LAF-237)**	303.4	Metabolic Disease	18.86%
**Aminothiazole**	100.14	Infection	18.90%
**Fluvastatin sodium (Lescol)**	433.45	Cardiovascular Disease	18.93%
**Bisacodyl**	361.39	Cardiovascular Disease	19.03%
**Tripelennamine HCl**	291.82	Neurological Disease	19.06%
**Carbachol**	182.65	N/A	19.09%
**Fexofenadine HCl**	538.12	Neurological Disease	19.09%
**Saxagliptin (BMS-477118,Onglyza)**	315.41	Infection	19.13%
**Estrone**	270.37	Endocrinology	19.21%
**Ribavirin (Copegus)**	244.2086	Metabolic Disease	19.22%
**Linagliptin (BI-1356)**	472.54	Cancer	19.22%

**Milrinone (Primacor)**	211.22	Cardiovascular Disease	19.25%
**Alverine Citrate**	473.56	Digestive system disease	19.31%
**Sodium ascorbate**	201.13	Endocrinology	19.43%
**Licofelone**	379.88	N/A	19.49%
**Decitabine**	228.21	Cardiovascular Disease	19.51%
**Eprosartan Mesylate**	520.62	Cardiovascular Disease	19.57%
**Chlorzoxazone**	169.57	Metabolic Disease	19.60%
**FK-506 (Tacrolimus)**	804.02	N/A	19.71%
**Pimecrolimus**	810.45	N/A	19.71%
**Daidzein**	254.24	Cardiovascular Disease	19.73%
**L-Adrenaline (Epinephrine)**	183.2	Cardiovascular Disease	19.74%
**PMSF (Phenylmethylsulfonyl Fluoride)**	174.19	Inflammation	19.77%
**Chlorocresol**	142.58	N/A	19.80%
**Rifampin (Rifadin, Rimactane)**	822.94	Infection	19.86%
**Isovaleramide**	101.15	Neurological Disease	19.87%
**Sulfadiazine**	250.28	Infection	19.87%
**Cyclamic acid**	179.24	Inflammation	19.89%
**Cefdinir (Omnicef)**	395.41	Infection	19.89%
**Mesna (Uromitexan, Mesnex)**	164.18	Cancer	19.91%
**Sulbactam**	233.24	Infection	19.93%
**Meptazinol HCl**	269.81	N/A	19.95%
**Dexamethasone acetate**	434.5	Inflammation	19.99%
**Aspirin (Acetylsalicylic acid)**	180.16	Cancer	20.06%
**Exemestane**	296.4	Endocrinology	20.06%
**Pasiniazid**	290.27	N/A	20.13%
**Ethynodiol diacetate**	384.51	Endocrinology	20.17%
**Entecavir hydrate**	295.29	Infection	20.17%
**Decamethonium bromide**	418.29	Neurological Disease	20.30%
**Elvitegravir (GS-9137)**	447.88	Immunology	20.30%
**Naphazoline hydrochloride (Naphcon)**	246.74	Neurological Disease	20.50%
**Azlocillin sodium salt**	484.48	Neurological Disease	20.56%
**Bemegride**	155.19	N/A	20.64%
**Homatropine Methylbromide**	370.28	N/A	20.69%
**Gallamine triethiodide (Flaxedil)**	891.53	Inflammation	20.72%
**Sulfamethazine**	278.33	Endocrinology	20.89%
**Piperacillin Sodium**	539.54	Infection	20.95%
**DL-Carnitine hydrochloride**	197.66	Cardiovascular Disease	21.04%
**Xylazine HCl**	256.79	Cardiovascular Disease	21.10%
**Darifenacin HBr**	507.46	Infection	21.10%
**Nelfinavir Mesylate**	663.89	N/A	21.12%

**Acebutolol HCl**	372.89	Neurological Disease	21.18%
**Sucralose**	397.63	N/A	21.18%
**Felbamate**	238.24	Neurological Disease	21.25%
**Ziprasidone hydrochloride**	449.4	Neurological Disease	21.28%
**Doxifluridine**	246.19	Immunology	21.34%
**Olopatadine hydrochloride (Opatanol)**	373.87	Neurological Disease	21.39%
**Bethanechol chloride**	196.68	Neurological Disease	21.45%
**Oxymetazoline hydrochloride**	296.84	Immunology	21.45%
**Naratriptan HCl**	371.93	Neurological Disease	21.51%
**Penicillin G Sodium**	356.37	Infection	21.55%
**Conivaptan HCl (Vaprisol)**	535.04	Cardiovascular Disease	21.58%
**Betamipron**	193.2	Infection	21.63%
**Dicloxacillin Sodium**	510.32	Infection	21.65%
**Retapamulin**	517.76	Neurological Disease	21.72%
**Moxonidine**	241.68	Digestive system disease	21.76%
**Aliskiren hemifumarate**	609.83	Cardiovascular Disease	21.79%
**Budesonide**	430.53	Endocrinology	21.80%
**Nicotine Ditartrate**	462.46	N/A	21.82%
**Adrucil (Fluorouracil)**	130.08	Cancer	21.86%
**Carbenicillin disodium**	422.36	Infection	21.89%
**Carprofen**	273.71	Inflammation	22.00%
**Albendazole (Albenza)**	265.33	Vermifuge	22.05%
**Benzocaine**	165.19	Respiratory Disease	22.12%
**Liothyronine Sodium**	672.96	Endocrinology	22.17%
**Ciclopirox (Penlac)**	207.27	Neurological Disease	22.20%
**Isosorbide**	146.14	N/A	22.25%
**Atorvastatin calcium (Lipitor)**	1155.34	Cardiovascular Disease	22.30%
**Aclidinium Bromide**	564.55	Neurological Disease	22.36%
**Acetylcysteine**	163.19	Respiratory Disease	22.41%
**Quinine hydrochloride dihydrate**	396.91	Cardiovascular Disease	22.46%
**Moexipril HCl**	535.03	Digestive system disease	22.52%
**Glimepiride**	490.62	Metabolic Disease	22.53%
**Gemfibrozil (Lopid)**	250.33	Cardiovascular Disease	22.53%

**Sorbitol (Glucitol)**	182.17	Digestive system disease	22.60%
**Febuxostat (Uloric)**	316.37	Inflammation	22.61%
**Acadesine**	258.23	Cardiovascular Disease	22.73%
**Quinapril HCl (Accupril)**	474.98	Inflammation	22.80%
**Fosfomycin Tromethamine**	259.19	N/A	22.85%
**Amidopyrine**	231.29	Neurological Disease	22.94%
**Sodium Picosulfate**	481.41	Metabolic Disease	22.98%
**Fosaprepitant dimeglumine**	1004.83	Cardiovascular Disease	23.02%
**Otilonium Bromide**	563.57	Cardiovascular Disease	23.05%
**Benserazide**	293.7	Neurological Disease	23.14%
**Ampicillin sodium**	371.39	Infection	23.19%
**Ampicillin Trihydrate**	403.45	Infection	23.19%
**Dehydroepiandrosterone (DHEA)**	288.43	Endocrinology	23.20%
**Prednisolone acetate (Omnipred)**	402.48	Immunology	23.23%
**Azithromycin Dihydrate**	785.02	Infection	23.29%
**Bazedoxifene HCl**	507.06	Metabolic Disease	23.30%
**Oxaprozin**	293.32	Inflammation	23.35%
**Betahistine 2HCl**	209.12	N/A	23.49%
**Pravastatin sodium**	446.51	Metabolic Disease	23.49%
**Lomerizine HCl**	541.46	Cardiovascular Disease	23.49%
**Choline Chloride**	139.62	N/A	23.53%
**Sodium nitrite**	69	Neurological Disease	23.68%
**Azacitidine (Vidaza)**	244.2	Cancer	23.69%
**Ropivacaine HCl**	310.86	Infection	23.75%
**Camylofin Chlorhydrate**	393.39	N/A	23.78%
**Mezlocillin Sodium**	561.56	Infection	23.84%
**Benazepril hydrochloride**	460.95	Cardiovascular Disease	23.90%
**Famciclovir (Famvir)**	321.3386	Cancer	23.92%
**Biotin (Vitamin B7)**	244.31	Infection	23.97%
**Beclomethasone dipropionate**	521.04	Inflammation	24.01%
**Florfenicol**	358.21	Infection	24.04%
**Allylthiourea**	116.18	Metabolic Disease	24.04%
**Teriflunomide**	270.21	Immunology	24.05%
**Catharanthine**	336.43	N/A	24.08%
**Tetracaine hydrochloride (Pontocaine)**	300.82	Endocrinology	24.10%

**Mercaptopurine**	152.18	Cancer	24.14%
**Escitalopram oxalate**	414.43	Infection	24.17%
**Fenspiride HCl**	296.79	Inflammation	24.17%
**Sulfamethizole (Proklar)**	270.33	Infection	24.24%
**nafcillin sodium monohydrate**	454.47	Endocrinology	24.26%
**Bromhexine HCl**	412.59	Cardiovascular Disease	24.30%
**Clofibric acid**	214.65	Metabolic Disease	24.40%
**Ganciclovir**	255.23	Infection	24.46%
**Ramelteon (TAK-375)**	259.34	Neurological Disease	24.49%
**Capecitabine (Xeloda)**	359.35	Cancer	24.54%
**Nifenazone**	308.33	N/A	24.55%
**Mepivacaine HCl**	282.81	Metabolic Disease	24.55%
**Methoxsalen (Oxsoralen)**	216.19	Inflammation	24.56%
**Streptozotocin (Zanosar)**	265.22	Cancer	24.58%
**Penfluridol**	523.97	Neurological Disease	24.63%
**Azaperone**	327.4	Neurological Disease	24.64%
**Cortisone acetate (Cortone)**	402.48	Cancer	24.66%
**Methazolastone**	194.15	Cancer	24.83%
**Etravirine (TMC125)**	435.28	Neurological Disease	24.88%
**Mepiroxol**	125.13	N/A	24.89%
**Triflusal**	248.16	Infection	24.89%
**Halobetasol Propionate**	484.96	Inflammation	24.89%
**BIBR-1048 (Dabigatran)**	627.73	Infection	24.94%
**Vandetanib (Zactima)**	475.35	Cancer	24.96%
**Pentoxifylline**	278.31	N/A	25.11%
**Tolvaptan (OPC-41061)**	448.94	Metabolic Disease	25.14%
**Ceftazidime Pentahydrate**	636.65	Infection	25.15%
**Azilsartan (TAK-536)**	456.45	Neurological Disease	25.16%
**Dibenzothiophene**	184.26	N/A	25.35%
**Bacitracin**	1408.67	Infection	25.46%
**Calcium Gluceptate**	490.42	N/A	25.46%
**Oxfendazole**	315.35	Vermifuge	25.50%
**Penciclovir**	253.26	Infection	25.58%
**Meloxicam (Mobic)**	351.4	Inflammation	25.68%
**Isoetharine Mesylate**	335.42	Cardiovascular Disease	25.68%
**Meclofenamate Sodium**	318.13	N/A	25.94%
**Pazopanib**	437.52	Cardiovascular Disease	25.97%
**Levosulpiride (Levogastrol)**	341.43	Neurological Disease	26.04%

**Ursodiol (Actigal Urso)**	392.57	Metabolic Disease	26.08%
**Fidaxomicin**	1058.04	Infection	26.15%
**Guanidine HCl**	95.53	Vermifuge	26.23%
**Altrenogest**	310.43	Neurological Disease	26.37%
**Losartan potassium**	462.01	Cardiovascular Disease	26.63%
**Carbimazole**	186.23	Infection	26.66%
**Droperidol**	379.43	Neurological Disease	26.69%
**Ifosfamide**	261.09	Cancer	26.78%
**Tilmicosin**	869.13	Infection	26.82%
**Pheniramine Maleate**	356.42	Neurological Disease	26.83%
**Doxofylline**	266.25	Metabolic Disease	26.84%
**Lansoprazole**	369.36	Infection	26.91%
**Geniposidic acid**	374.34	N/A	26.97%
**Tofacitinib citrate (CP-690550 citrate)**	504.49	N/A	27.08%
**Ethoxzolamide**	258.32	Neurological Disease	27.11%
**Itraconazole (Sporanox)**	705.6503	Cancer	27.15%
**Cloxacillin sodium (Cloxacap)**	475.88	Cardiovascular Disease	27.18%
**Azelnidipine**	582.65	Neurological Disease	27.35%
**Caspofungin acetate**	1213.42	Infection	27.50%
**Xylometazoline HCl**	280.84	Infection	27.51%
**Beta Carotene**	536.87	N/A	27.57%
**Mitoxantrone Hydrochloride**	517.4	Cardiovascular Disease	27.63%
**Leflunomide**	270.21	Inflammation	27.71%
**Paeoniflorin**	480.46	N/A	27.76%
**Troxipide**	294.35	Digestive system disease	27.91%
**Gliclazide (Diamicron)**		323.41	Neurological
**Ammonium Glycyrrhizinate**	839.96	N/A	27.99%
**Fludarabine (Fludara)**	285.23	Cancer	28.09%
**Amoxicillin (Amoxycillin)**	365.4	Neurological Disease	28.41%
**Loratadine**	382.88	Inflammation	28.50%
**Closantel Sodium**	685.06	Vermifuge	28.58%
**Pyrithione zinc**	317.7	Infection	28.72%
**Tianeptine sodium**	458.93	Neurological Disease	28.89%
**Sodium Nitroprusside**	261.92	Cardiovascular Disease	28.89%
**Betapar (Meprednisone)**	372.455	Inflammation	28.97%
**Oxybutynin (Ditropan)**	357.49	Neurological Disease	29.29%

**Betamethasone Dipropionate**	504.59	N/A	29.50%
**Lopinavir (ABT-378)**	628.8	Infection	29.50%
**Mitotane (Lysodren)**	320.04	Cancer	29.50%
**Cefditoren pivoxil**	620.72	Infection	29.62%
**Ulipristal**	475.62	Infection	29.73%
**Tolfenamic acid**	261.7	Inflammation	29.86%
**Azilsartan Medoxomil (TAK-491)**	568.53	Cardiovascular Disease	30.61%
**Idebenone**	338.44	Inflammation	30.94%
**L-Thyroxine**	776.87	Neurological Disease	31.11%
**Ketorolac (Toradol)**	376.4	Neurological Disease	31.26%
**Alfacalcidol**	400.64	Endocrinology	31.26%
**Terfenadine**	471.67	N/A	31.29%
**Estradiol valerate**	356.5	Endocrinology	31.38%
**Chromocarb**	190.15	Cardiovascular Disease	31.42%
**2-Methoxyestradiol**	302.41	Cancer	31.67%
**Racecadotril (Acetorphan)**	385.48	Infection	32.01%
**Metronidazole (Flagyl)**	171.15	Infection	32.12%
**toltrazuril**	425.38	Infection	32.14%
**Loteprednol etabonate**	466.95	N/A	32.15%
**Atovaquone (Atavaquone)**	366.84	Neurological Disease	32.37%
**Deoxyarbutin**	194.23	Cardiovascular Disease	32.39%
**Diethylstilbestrol (Stilbestrol)**	268.35	Cancer	32.45%
**Oxybutynin chloride**	393.95	Neurological Disease	32.49%
**Estradiol Cypionate**	396.56	N/A	32.49%
**Bextra (valdecoxib)**	314.36	Neurological Disease	32.53%
**Clofoctol**	365.34	N/A	32.63%
**Dexlansoprazole**	369.36	Cardiovascular Disease	32.67%
**Phenoxybenzamine HCl**	340.3	Endocrinology	32.99%
**Clopidogrel (Plavix)**	419.9	Cardiovascular Disease	33.02%
**Vitamin D2**	396.65	Endocrinology	33.30%
**Vismodegib (GDC-0449)**	421.3	Cancer	33.31%
**Benzoic acid**	122.12	N/A	33.68%
**Rolipram**	275.34	Inflammation	33.74%
**Probenecid (Benemid)**	285.36	Metabolic Disease	34.04%
**Phenytoin sodium (Dilantin)**	274.25	Metabolic Disease	34.09%
**Clindamycin palmitate HCl**	699.85	Infection	34.14%

**Pyridoxine hydrochloride**	205.64	Endocrinology	34.15%
**Cleviprex (Clevidipine)**	456.32	Cardiovascular Disease	34.20%
**Prasugrel (Effient)**	373.44	Cardiovascular Disease	34.25%
**Butenafine HCl**	353.93	Neurological Disease	34.43%
**Calcitriol (Rocaltrol)**	416.64	Endocrinology	34.50%
**Fulvestrant (Faslodex)**	606.77	Cancer	34.50%
**Carbenoxolone Sodium**	614.72	N/A	34.85%
**Camptothecin**	348.35	Cancer	35.06%
**Oxethazaine**	467.64	Neurological Disease	35.44%
**Zafirlukast (Accolate)**	575.68	Inflammation	35.68%
**Erlotinib HCl**	429.9	Cancer	35.71%
**Difloxacin HCl**	259.19	N/A	36.17%
**PCI-32765 (Ibrutinib)**	440.5	Neurological Disease	36.29%
**Ethinyl Estradiol**	296.4	Endocrinology	37.01%
**Benzethonium chloride**	448.08	Neurological Disease	37.63%
**Progesterone (Prometrium)**	314.46	Endocrinology	37.74%
**Estradiol**	272.38	Endocrinology	38.05%
**Doxercalciferol (Hectorol)**	412.65	Endocrinology	39.50%
**Indacaterol Maleate**	508.56	Infection	39.50%
**Pimozide**	461.55	N/A	39.88%
**Artemisinin**	282.33	Infection	40.97%
**Vitamin D3 (Cholecalciferol)**	384.64	Cardiovascular Disease	41.73%
**Naftopidil (Flivas)**	392.49	Endocrinology	42.19%
**S-(+)-Rolipram**	275.34	Cardiovascular Disease	42.72%
**AMG-073 HCl (Cinacalcet hydrochloride)**	393.87	Endocrinology	42.77%
**Trilostane**	329.43	Endocrinology	43.07%
**Paclitaxel (Taxol)**	853.91	Cancer	45.50%
**Terbinafine hydrochloride (Lamisil)**	327.89	Infection	45.83%
**Drospirenone**	366.49	Endocrinology	45.84%
**Repaglinide**	452.59	Endocrinology	46.56%
**Manidipine (Manyper)**	610.7	Metabolic Disease	46.78%
**Gestodene**	310.43	Endocrinology	47.10%
**Lithocholic acid**	376.57	Neurological Disease	47.19%
**Phenytoin (Lepitoin)**	252.27	Endocrinology	47.92%
**Pimobendan (Vetmedin)**	334.37	Cardiovascular Disease	48.26%
**Pioglitazone (Actos)**	356.44	Cancer	49.05%

**Vemurafenib (PLX4032)**	489.92	Cancer	49.85%
**Idoxuridine**	354.1	Infection	50.85%
**Lacidipine (Lacipil, Motens)**	455.54	Cardiovascular Disease	51.86%
**Pioglitazone hydrochloride (Actos)**	392.9	Metabolic Disease	52.55%
**Levonorgestrel (Levonelle)**	312.45	Endocrinology	53.45%
**Rosiglitazone HCl**	393.89	Cardiovascular Disease	53.56%
**DAPT (GSI-IX)**	432.46	Cancer	54.30%
**Rosiglitazone maleate**	473.5	Infection	54.76%
**Isotretinoin**	300.44	Metabolic Disease	55.43%
**Rosiglitazone (Avandia)**	357.43	Cancer	55.71%
**Nystatin (Mycostatin)**	926.09	Infection	56.43%
**Chlorquinaldol**	228.07	Infection	56.54%
**Lomustine (CeeNU)**	233.7	Cancer	56.61%
**Bexarotene**	348	Cardiovascular Disease	57.29%
**Doxazosin mesylate**	547.58	Cardiovascular Disease	60.88%
**Artemether (SM-224)**	298.37	Cancer	61.17%
**Chenodeoxycholic acid**	392.57	Infection	61.59%
**Esomeprazole sodium (Nexium)**	367.4	Cancer	62.64%
**Carmofur**	257.26	Cancer	68.01%
**Betamethasone valerate (Betnovate)**	476.58	Inflammation	69.09%
**Domperidone (Motilium)**	425.91	Neurological Disease	70.89%
**Pranlukast**	481.5	Immunology	71.26%
**Adapalene**	412.52	Inflammation	75.05%
**Esomeprazole magnesium (Nexium)**	713.12	Digestive system disease	75.50%
**Amorolfine Hydrochloride**	353.97	Infection	75.79%
**norethindrone**	298.42	Neurological Disease	76.25%
**Rimonabant (SR141716)**	463.79	Inflammation	76.40%
**Cyclandelate**	276.37	Neurological Disease	80.12%
**Omeprazole (Prilosec)**	345.42	Metabolic Disease	80.50%
**Cephalomannine**	831.9	Cancer	80.78%
**Bezafibrate**	361.82	Metabolic Disease	87.03%
**Aminoglutethimide (Cytadren)**	232.28	Endocrinology	Inhibitor
**Amlodipine (Norvasc)**	408.88	Cardiovascular Disease	Inhibitor
**Amlodipine besylate (Norvasc)**	567.05	Cardiovascular Disease	Inhibitor

**Amphotericin B**	924.08	N/A	Inhibitor
**Anastrozole**	293.37	Endocrinology	Inhibitor
**Apatinib (YN968D1)**	493.58	Cancer	Inhibitor
**Azathioprine (Azasan, Imuran)**	277.26	Immunology	Inhibitor
**Bifonazole**	310.39	Infection	Inhibitor
**Butoconazole nitrate**	474.79	Infection	Inhibitor
**Carbazochrome sodium sulfonate**	322.27	Cancer	Inhibitor
**Cetrimonium Bromide**	364.45	Infection	Inhibitor
**Cetylpyridinium Chloride**	339.99	Infection	Inhibitor
**Climbazole**	292.76	Infection	Inhibitor
**Clotrimazole (Canesten)**	344.84	Infection	Inhibitor
**Cobicistat (GS-9350)**	776.02	Cancer	Inhibitor
**Curcumin**	368.38	N/A	Inhibitor
**Detomidine HCl**	222.71	Cardiovascular Disease	Inhibitor
**Dexmedetomidine**	200.28	Neurological Disease	Inhibitor
**Dexmedetomidine HCl (Precedex)**	236.74	Neurological Disease	Inhibitor
**Domiphen Bromide**	414.46	Infection	Inhibitor
**Duloxetine HCl (Cymbalta)**	333.88	Neurological Disease	Inhibitor
**Econazole nitrate (Spectazole)**	444.7	Neurological Disease	Inhibitor
**Etomidate**	244.29	Neurological Disease	Inhibitor
**Fenticonazole nitrate**	518.41	Neurological Disease	Inhibitor
**Fluconazole**	306.27	Infection	Inhibitor
**Fluvoxamine maleate**	434.41	Neurological Disease	Inhibitor
**Imatinib (Gleevec)**	493.6	Neurological Disease	Inhibitor
**Imatinib Mesylate**	589.71	Cancer	Inhibitor
**Isoconazole nitrate (Travogen)**	479.14	Infection	Inhibitor
**Ketoconazole**	531.43	Infection	Inhibitor
**Letrozole**	285.3	Endocrinology	Inhibitor
**Masitinib (AB1010)**	498.64	Respiratory Disease	Inhibitor
**Medetomidine HCl**	236.74	Infection	Inhibitor
**Miconazole (Monistat)**	416.13	Neurological Disease	Inhibitor
**Miconazole nitrate**	479.14	Infection	Inhibitor
**Milnacipran HCl**	282.81	Endocrinology	Inhibitor
**Nicardipine HCl**	515.99	Neurological Disease	Inhibitor
**Nicorandil (Ikorel)**	211.17	Cardiovascular Disease	Inhibitor
**Nilvadipine (ARC029)**	385.37	Cancer	Inhibitor

**Nimesulide**	308.31	Cardiovascular Disease	Inhibitor
**Nimodipine (Nimotop)**	418.44	Cardiovascular Disease	Inhibitor
**Ospemifene**	378.89	N/A	Inhibitor
**Ozagrel**	228.25	Cardiovascular Disease	Inhibitor
**Ozagrel HCl**	264.71	Cardiovascular Disease	Inhibitor
**Pilocarpine HCl**	244.72	Neurological Disease	Inhibitor
**Piroxicam (Feldene)**	331.35	Inflammation	Inhibitor
**Posaconazole**	700.78	Infection	Inhibitor
**Primaquine Diphosphate**	455.34	N/A	Inhibitor
**Protionamide (Prothionamide)**	180.27	Infection	Inhibitor
**Ritonavir**	720.94	Infection	Inhibitor
**Roflumilast (Daxas)**	403.21	Neurological Disease	Inhibitor
**Sertaconazole nitrate**	500.78	Infection	Inhibitor
**Sulconazole Nitrate**	460.76	Infection	Inhibitor
**Tazarotene (Avage)**	351.46	Inflammation	Inhibitor
**Tioconazole**	387.71	Infection	Inhibitor
**Tiratricol**	621.93	Endocrinology	Inhibitor
**Tolcapone**	273.24	Metabolic Disease	Inhibitor
**Tretinoin (Aberela)**	300.4	Cancer	Inhibitor
**Tropicamide**	284.35	Neurological Disease	Inhibitor
**Valaciclovir HCl**	360.8	Infection	Inhibitor
**Voriconazole**	349.31	Infection	Inhibitor
**Zoxazolamine**	168.58	Neurological Disease	N/A
**Adrenalone HCl**	217.65	Cardiovascular Disease	NSB
**Afatinib (BIBW2992)**	485.94	Cancer	NSB
**Avobenzone (Parsol 1789)**	310.39	N/A	NSB
**Bephenium Hydroxynaphthoate**	443.53	N/A	NSB
**Bergapten**	216.19	Cancer	NSB
**Bosutinib (SKI-606)**	530.45	Cancer	NSB
**Clonidine hydrochloride (Catapres)**	266.5	Infection	NSB
**Dacarbazine (DTIC-Dome)**	182.18	Cancer	NSB
**Glafenine HCl**	409.26	N/A	NSB
**Irinotecan**	586.68	Cancer	NSB
**Malotilate**	288.38	Metabolic Disease	NSB
**Marbofloxacin**	362.36	N/A	NSB

**Mefenamic acid**	241.29	Cardiovascular Disease	NSB
**Mesoridazine Besylate**	544.75	Neurological Disease	NSB
**Moxifloxacin hydrochloride**	437.89	Infection	NSB
**Natamycin (Pimaricin)**	665.73	Infection	NSB
**Regorafenib (BAY 73-4506)**	482.82	Cancer	NSB
**Secnidazole (Flagentyl)**	185.18	Infection	NSB
**Sorafenib (Nexavar)**	637.03	Cancer	NSB
**Tolmetin Sodium**	315.3	N/A	NSB
**Tranilast (SB 252218)**	327.33	Respiratory Disease	NSB
**9-Aminoacridine**	194.23	N/A	PD
**Acitretin**	326.43	Infection	PD
**Alprostadil (Caverject)**	354.48	Endocrinology	PD
**Amiodarone HCl**	681.77	Cardiovascular Disease	PD
**Aripiprazole (Abilify)**	448.39	Neurological Disease	PD
**Benidipine hydrochloride**	542.02	Cardiovascular Disease	PD
**Carbadox**	262.22	Infection	PD
**Clofazimine**	473.4	Infection	PD
**Clomifene citrate (Serophene)**	598.08	Cancer	PD
**Closantel**	663.07	Vermifuge	PD
**Crystal violet**	407.98	Infection	PD
**Dabrafenib (GSK2118436)**	519.56	Infection	PD
**Dronedarone HCl (Multaq)**	593.22	Neurological Disease	PD
**Eltrombopag (SB-497115-GR)**	564.63	Cancer	PD
**Entacapone**	305.29	Neurological Disease	PD
**Epalrestat**	319.4	Inflammation	PD
**Ezetimibe (Zetia)**	409.4	Cardiovascular Disease	PD
**Felodipine (Plendil)**	384.25	Cardiovascular Disease	PD
**Fenofibrate (Tricor, Trilipix)**	360.83	Cardiovascular Disease	PD
**Fluocinonide (Vanos)**	494.52	Endocrinology	PD
**Fluticasone propionate (Flonase, Veramyst)**	500.57	Inflammation	PD
**Gliquidone**	527.63	Metabolic Disease	PD
**Ipratropium bromide**	412.37	Respiratory Disease	PD
**Irinotecan HCl Trihydrate (Campto)**	677.18	Neurological Disease	PD

**Lapatinib**	581.06	Neurological Disease	PD
**Lapatinib Ditosylate (Tykerb)**	925.46	Cancer	PD
**Levosimendan**	280.28	Metabolic Disease	PD
**Mestranol**	310.43	Endocrinology	PD
**Montelukast Sodium**	608.17	Respiratory Disease	PD
**Nisoldipine (Sular)**	388.41	Cardiovascular Disease	PD
**Nitazoxanide (Alinia, Annita)**	307.28	Vermifuge	PD
**olsalazine sodium**	346.2	Inflammation	PD
**Phenazopyridine HCl**	249.7	Cardiovascular Disease	PD
**Phenothrin**	350.45	N/A	PD
**Pregnenolone**	316.48	Neurological Disease	PD
**Sunitinib Malate (Sutent)**	532.56	Cancer	PD
**Tamoxifen Citrate (Nolvadex)**	563.64	Endocrinology	PD
**Tenoxicam (Mobiflex)**	337.3783	Infection	PD
**Ticagrelor**	522.57	Cardiovascular Disease	PD
**Tolnaftate**	307.41	Cancer	PD
**Topotecan HCl**	457.91	Cancer	PD
**Toremifene Citrate (Fareston, Acapodene)**	598.08	Endocrinology	PD
**triamterene**	253.26	Inflammation	PD
**Triclabendazole**	359.66	Vermifuge	PD
**Verteporfin (Visudyne)**	718.79	Endocrinology	PD

Table S2. Summary table of BM3 WT and DM conversions and metabolite production of structurally diverse pharmaceuticals. The percentage conversion (% conv) and selectivity (% sel) for BM3 WT and DM-derived metabolites (M) were calculated by determining the peak area ratio of the parent compound in the peak chromatogram in comparison to the culminative peak area of all compounds, and by determining the peak area ratio of the metabolite in the peak chromatogram in comparison to the culminative peak area of all metabolites, respectively. Fully structurally elucidated metabolites schematics are shown, with the related human P450 isoform stated in brackets if relevant. N/A indicates the metabolite was not produced by the BM3 enzyme. An asterisk (*) denotes the presence of several adducts in the MS led to selectivity ratios being approximated. All MS and NMR data are shown in Supplementary Figures S1-S31.

**Drug**	**Parent structure**	**% conv**		**M**	**Metabolite structure**	**% sel**	
		**WT**	**DM**			**WT**	**DM**
**Artemisinin**	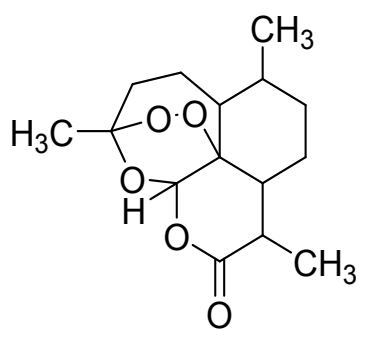	91	79	AM1*	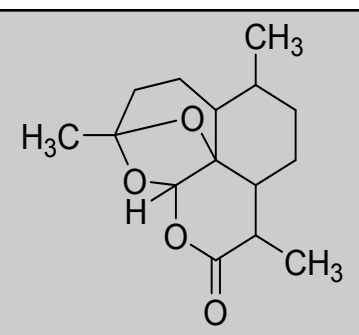 Deoxy-artemisinin (Human CYP2B6)	17	5
				AM2*	Unknown modification	3	3
				AM3*	Unknown modification	31	5
				AM4*	M mixture unknown modification	66	93
**Estradiol**	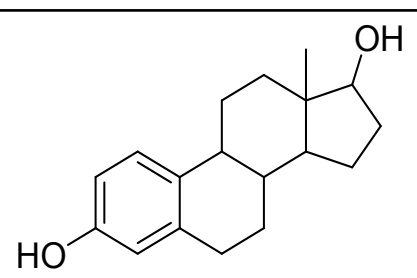	2	100	EM1	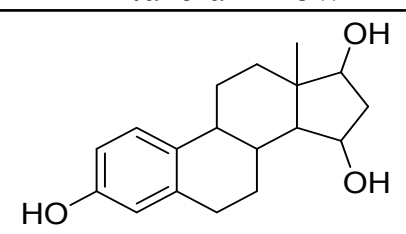 15-β-OH-estradiol (Unknown human CYP)	100	78
				EM2	Dihydroxylation	N/A	14
				EM3	M mixture unknown modification	N/A	8
**Rosiglitazone**	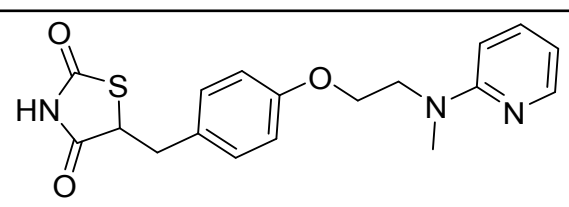	87	100	RoM1	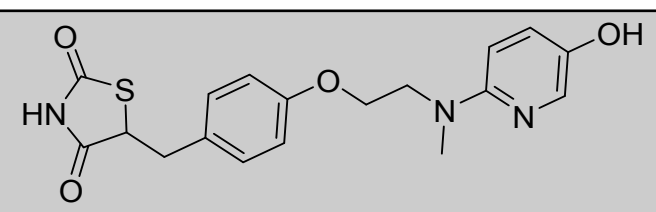 20-OH-rosiglitazone (Human CYP2C8/9)	31	15 (10m)
				RoM2	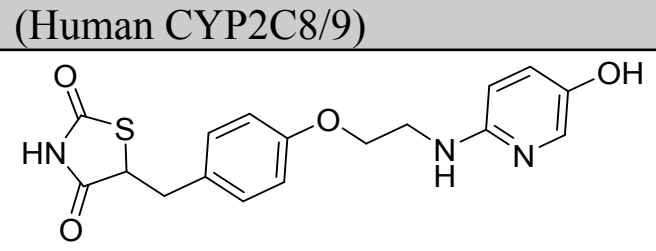 20-OH-N-desme-rosiglitazone (Human CYP2C8/9)	2	9 (10m)
				RoM3	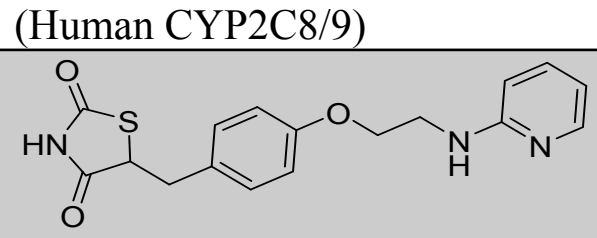 N-desme-rosiglitazone (Human CYP2C8/9)	20	70 (1m), 13 (10m)
				RoM4	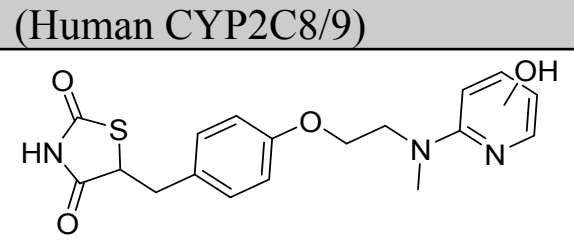 Ar-OH- rosiglitazone	24	33 (10m)
				RoM5	Unknown modification	2	4 (1m)

				RoM6	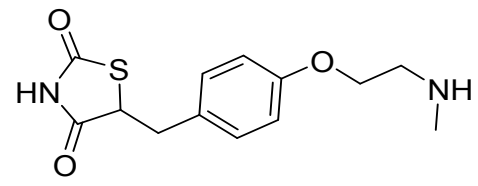 Despyridin-rosiglitazone (Human CYP2C8/9))	21	26 (1m), 24 (10m), 62 (180m)
				RoM7	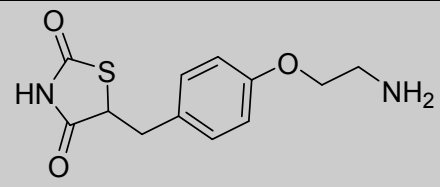 Despyridin-N-desme-rosiglitazone	N/A	3 (10m), 30 (180m)
				RoM8	Unknown modification	N/A	4 (10m)
				RoM9	Unknown modification	N/A	8 (180m)
**Pioglitazone**	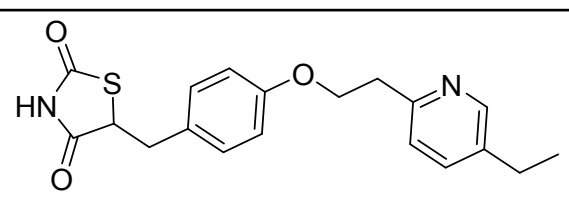	88	100	PiM1	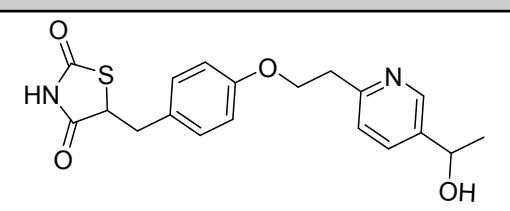 Ar-OH-pioglitazone (Human CYP2C8)	N/A	(20m)
				PiM2	Unknown modification/dihydroxylation	N/A	4 (10m)
				PiM3	Unknown modification	N/A	8 (180m)
**Troglitazone**	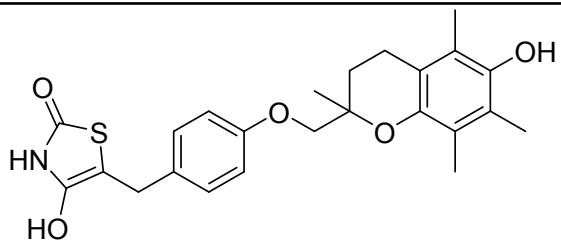	100	100	TM1	Desaturation or oxidation	71	56
				TM2	Unknown modification	10	3
				TM3	Hydroxylation	9	12
				TM4	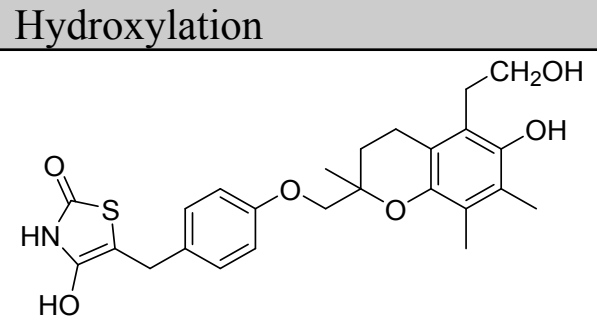 30-OH-troglitazone	12	12
				TM5	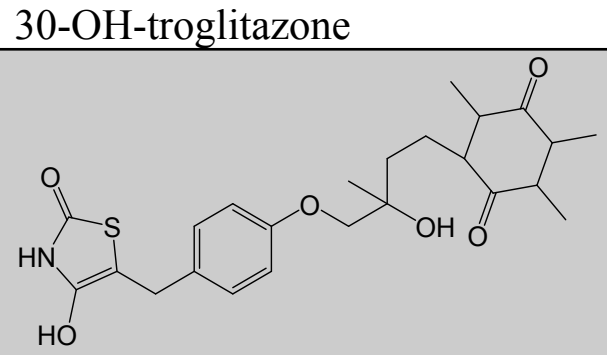 Quinone formation	N/A	3
				TM6	Dihydroxylation	N/A	3
				TM7	Dihydroxylation	N/A	2
				TM8	Unknown modification	N/A	3
				TM9	Unknown modification	N/A	2.9
				TM10	Unknown modification	N/A	3.19
**Repaglinide**	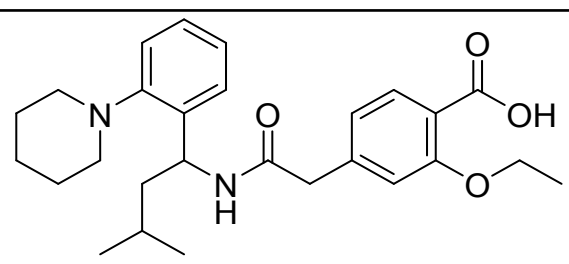	8	100	ReM1	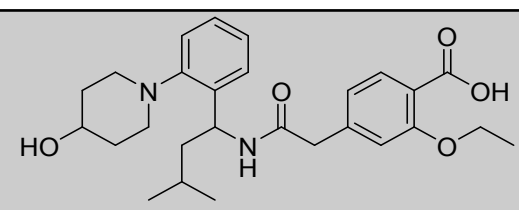 21-OH-repaglinide	48	44 (20m), 60 (180m)
				ReM2	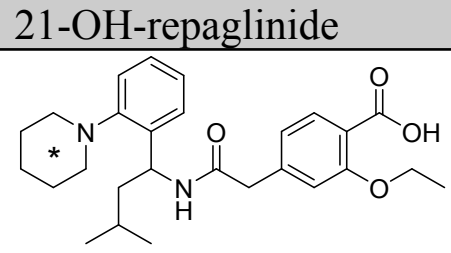 Desaturation	22	14 (20m), 7 (180m)

				ReM3	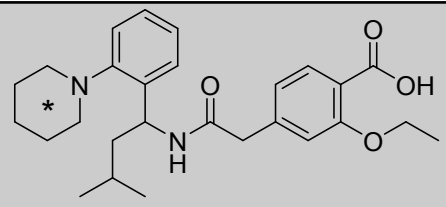<br>Desaturation	30	27 (20m), 11 (180m)
				ReM4	Unknown modification	N/A	1 (20m), 2 (180m)
				ReM5	Unknown modification	N/A	2 (180m)
				ReM6	Hydroxylation	N/A	1 (20m), 2 (180m)
				ReM7	Hydroxylation	N/A	2 (20m), 1 (180m)
				ReM8	Hydroxylation	N/A	3 (20m), 2 (180m)
				ReM9	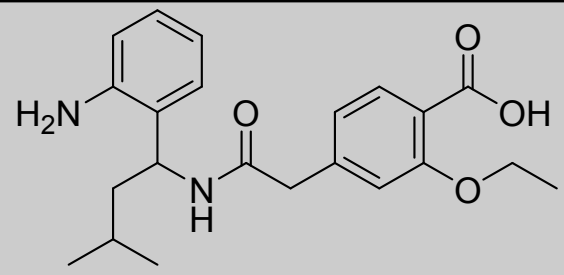<br>Despiperidin-repaglinide (Human CYP3A4/5)	N/A	1 (20m), 2 (180m)
				ReM10	Unknown modification	N/A	3 (180m)
				ReM11	Hydroxylation	N/A	1 (20m), 2 (180m)
				ReM12	Ketone formation	N/A	2 (20m)
				ReM13	Ketone formation	N/A	3 (20m)

Table S3: Table of EPR g-values for the WT and DM heme domain proteins bound to a variety of antidiabetic compounds

Compound	Rhombic LS signals ($g_z/g_y/g_x$)	
	Major species	Minor species
**WT Heme**	2.41/2.25/1.91	None
**WT Heme + DMSO**	2.41/2.25/1.91	2.44/2.25/1.90
**WT Heme + Troglitazone**	2.44/2.25/1.90	2.41/2.25/1.91
**DM Heme**	2.43/2.25/1.91	2.40/2.25/1.92; 2.51/2.25/1.89
**DM Heme + DMSO**	2.45/2.25/1.91	2.51/2.25/1.89
**DM Heme + Darglitazone**	2.44/2.25/1.91	None
**DM Heme + Pioglitazone**	2.45/2.25/1.91	2.51/2.25/1.89
**DM Heme + Rosiglitazone**	2.45/2.25/1.91	2.51/2.25/1.89
**DM Heme + Troglitazone**	LS 2.45/2.25/1.91<br>HS 7.94/3.64/1.70	2.39/2.25/1.97; 2.51/2.25/1.89
**DM Heme + Mitiglinide**	2.45/2.25/1.91	2.51/2.25/1.89
**DM Heme + Nateglinide**	2.44/2.25/1.91	2.47/2.25/1.90
**DM Heme + Repaglinide**	2.44/2.25/1.91	2.47/2.25/1.90
**DM Heme + Metformin**	2.43/2.25/1.91	2.40/2.25/1.92; 2.51/2.25/1.89

Table S4: Table of crystallographic data

	DM BM3 + Troglitazone
PDB ID	6HN8
Data collection	
Space group	$P2_12_12_1$
Cell dimensions	
a, b, c (Å)	60.9 120.4 146.0
α, β, γ (°)	90 90 90
Resolution range (Å)	29.5-2.00
R_{meas}	0.149 (0.952)
$I / \sigma I$	10.8 (2.2)
Completeness (%)	99.4 (95.1)
Redundancy	6.6 (6.4)
Refinement	
Resolution (Å)	29.5-2.00
No. reflections	73144
R_{work} / R_{free}	15.9/18.7 (20.8/23.3)
No. of residues	
Protein	910
Water	903
Ligand/ion	2
B-Factors (Å²)	
Protein	28.9
Ligand	33.2
R.m.s. deviations	
Bond lengths (Å)	0.003
Bond angles (°)	0.86
Ramachandran	
Favored (%)	96.9
Outlier (%)	0.1

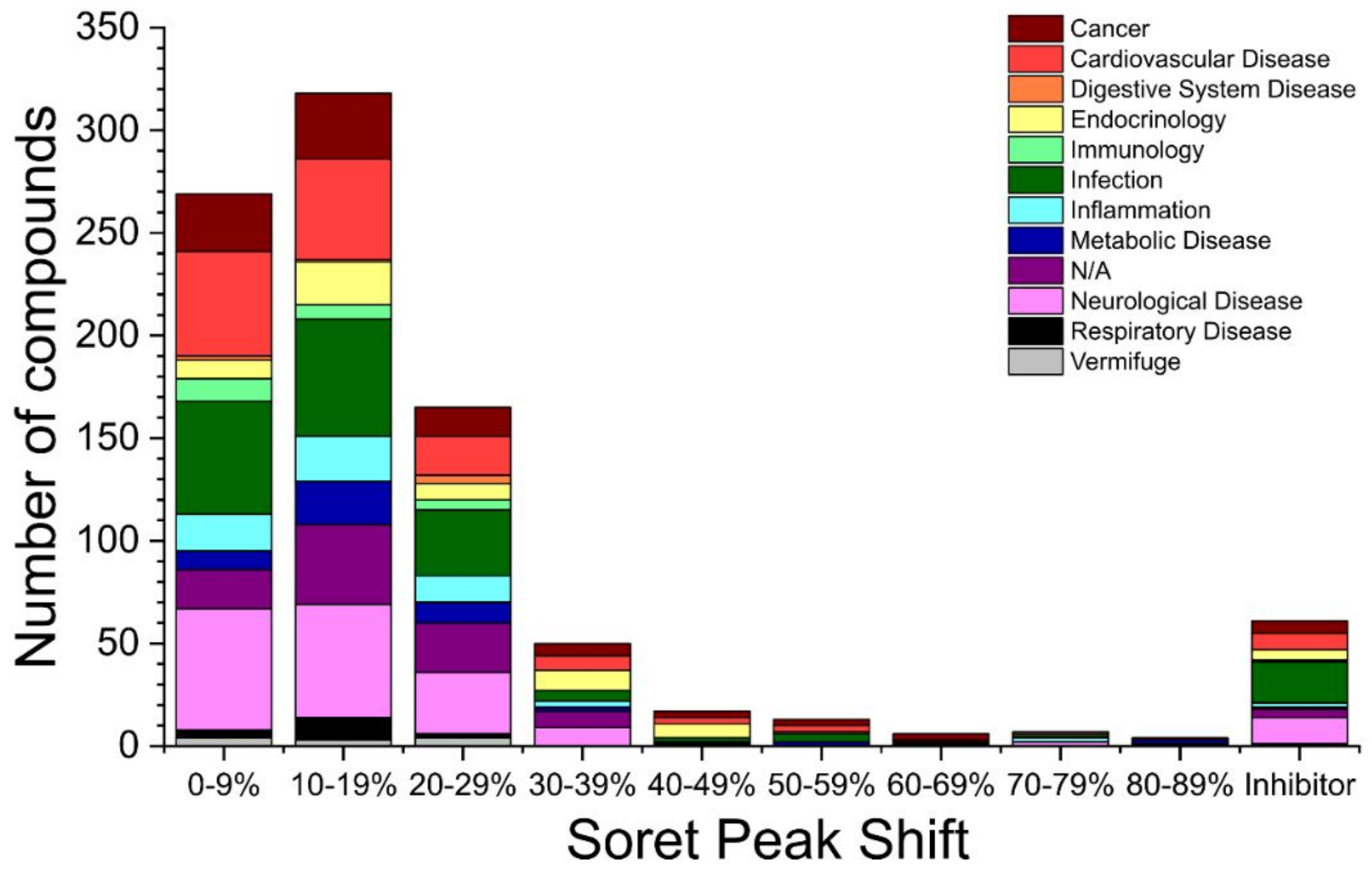

Figure S1: BM3 DM Soret peak shift by compound classification.

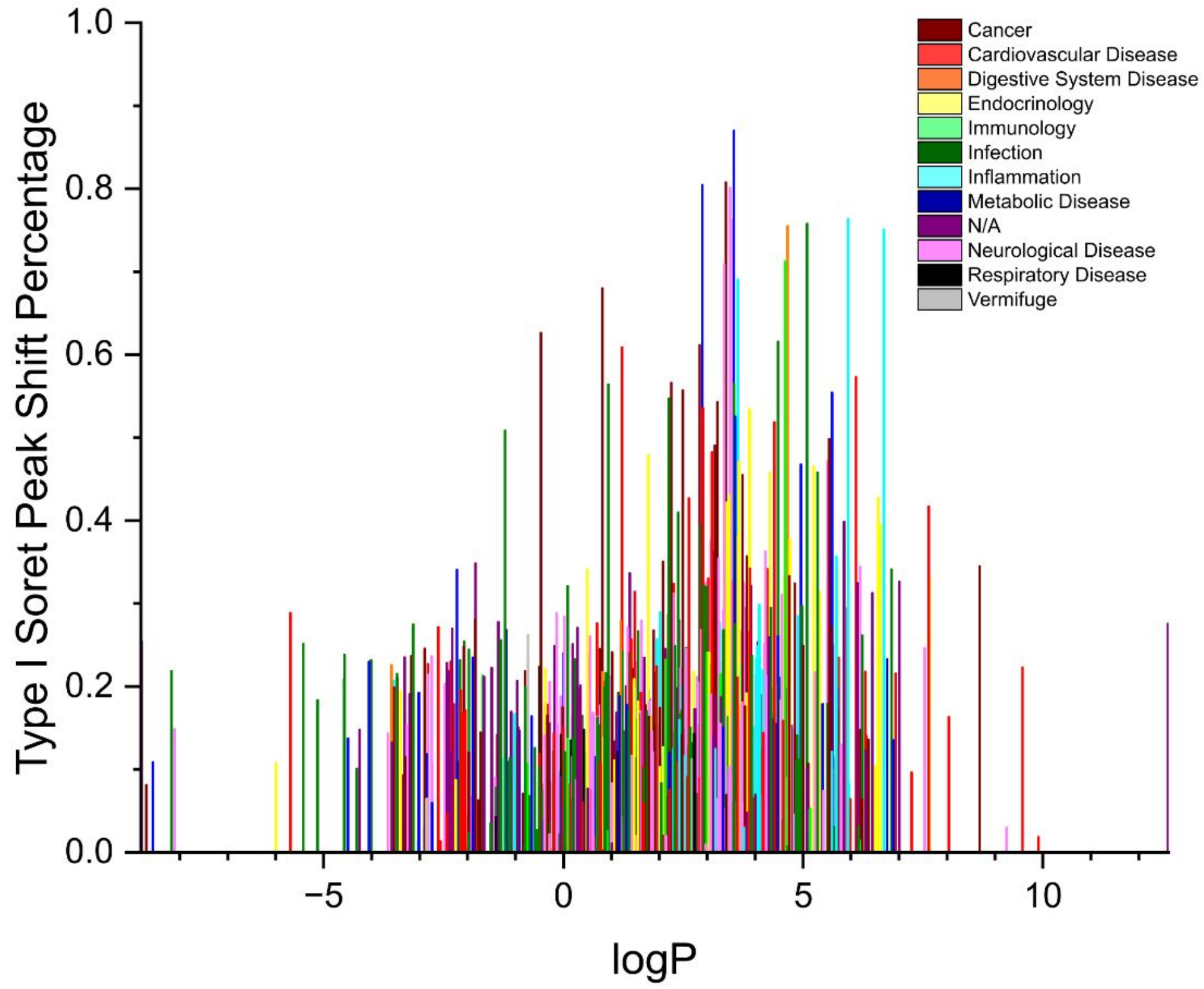

Figure S2: BM3 DM Soret peak shift by compound classification and logP value. A value of 1.0 corresponds to 100% shift as observed with the compound N-palmitoyl glycine.

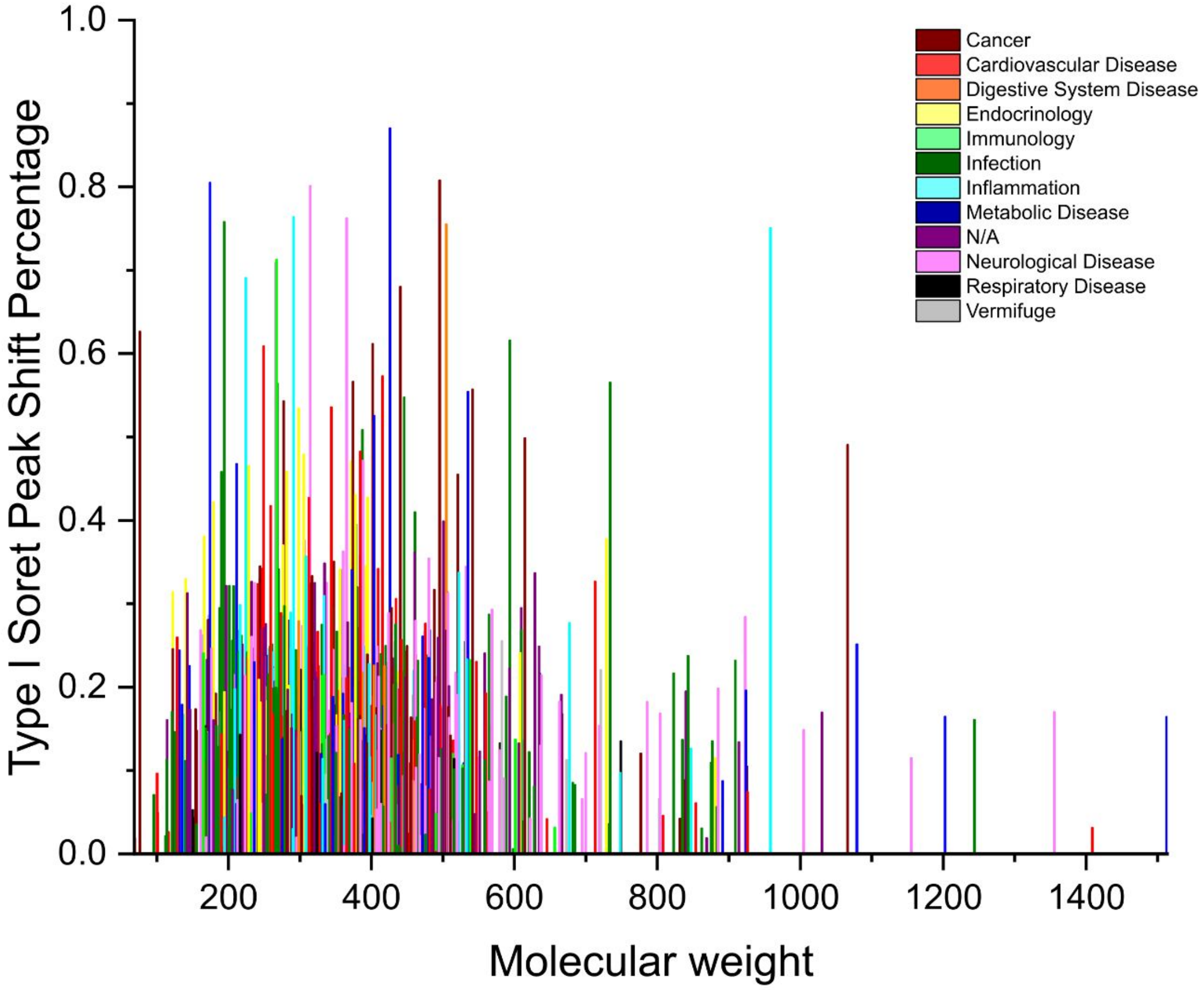

Figure S3: BM3 DM Soret peak shift by compound classification and molecular weight. A value of 1.0 corresponds to 100% shift as observed with the compound N-palmitoyl glycine.

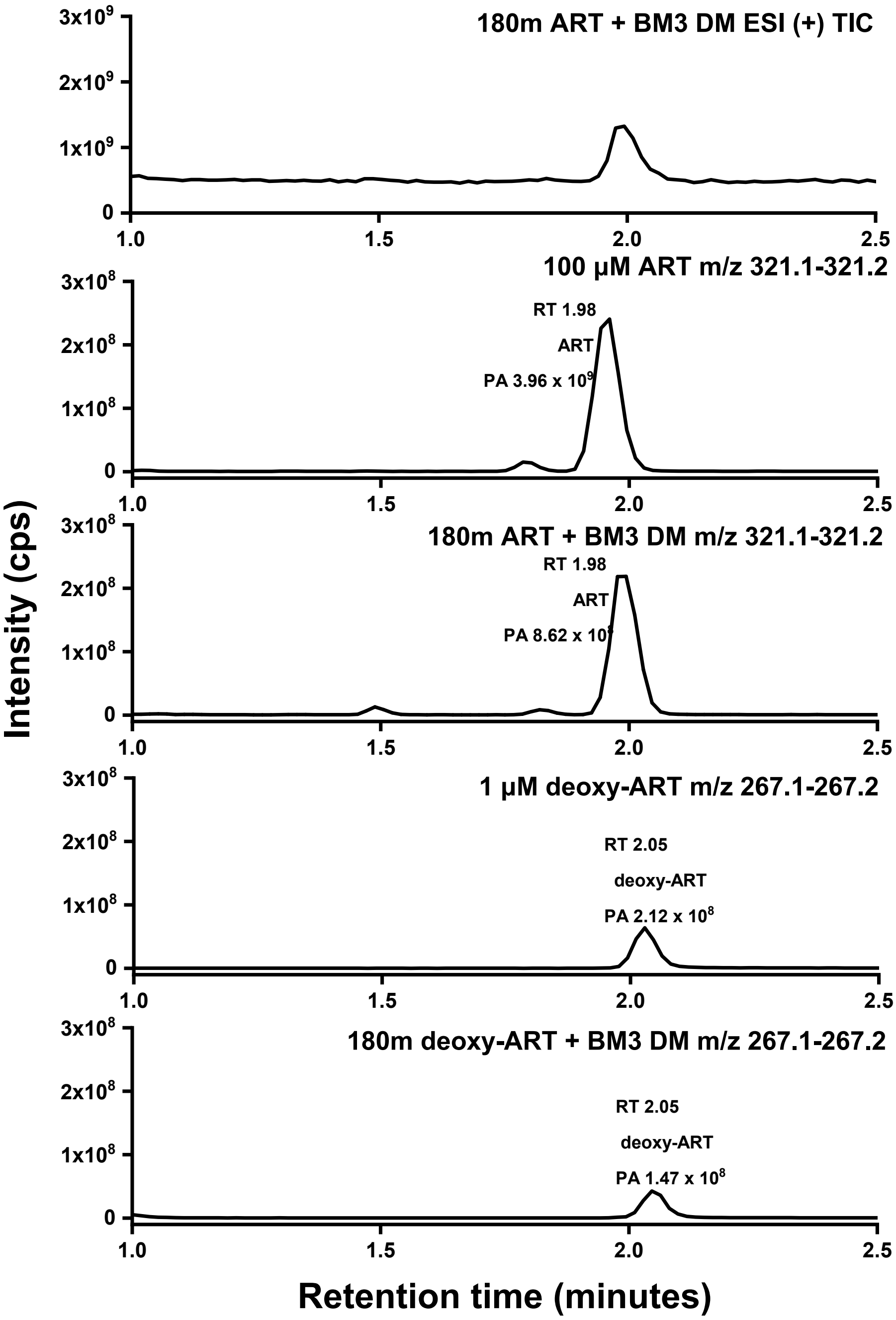

Figure S4: ESI (+) LC-MS/MS peak chromatogram of BM3 DM turnover of artemisinin (ART) at 180 minutes. Mass range windows (±0.1 Da) displayed for parent (artemisinin) and its main metabolite (deoxyartemisinin [deoxy-ART]). Artemisinin and deoxyartemisinin standards are also displayed.

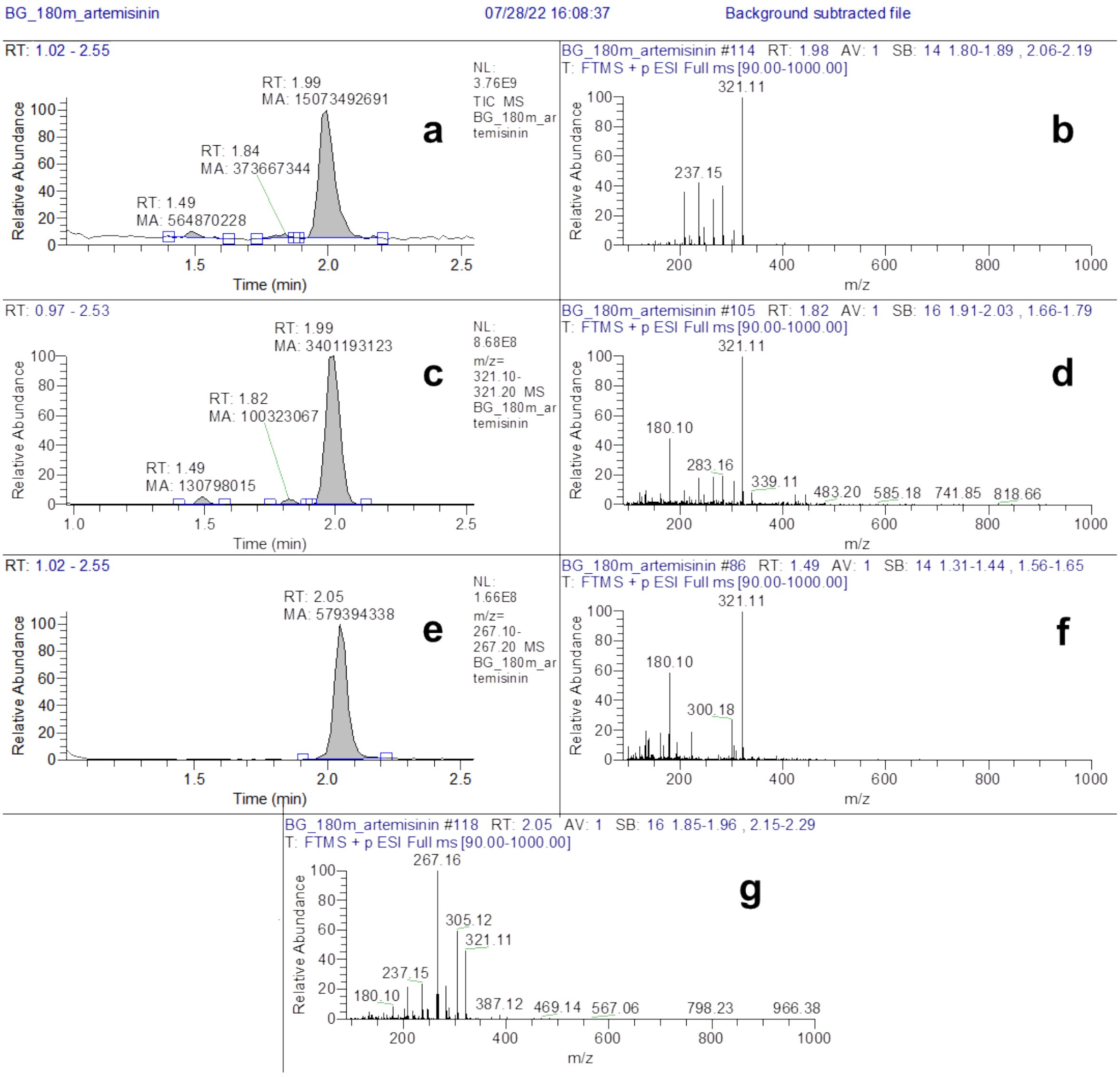

Figure S5: LC-MS/MS chromatograms of ART and BM3 DM incubation.
Panel a shows TIC peak chromatogram with peak areas (MA) indicated for identified parent compound and metabolites. Panel b shows ion chromatogram at RT 1.98 minutes, most abundant species at *m/z* 321.1 is indicative of ART $[M+K]^+$, minor adducts correlate to ART standard adducts (Figure S1). Panel c shows *m/z* 321.1-321.2 peak chromatogram with peak areas (MA) indicated for ART at RT 1.99, DM-AM2 at RT 1.82 and DM-AM3 at 1.49. Panel d shows ion chromatogram at RT 1.82 minutes, most abundant species at *m/z* 321.1 of unknown metabolite DM-AM2. Panel e shows *m/z* 267.1-267.2 peak chromatogram with peak areas (MA) indicated for DM-AM1 at RT 2.05 minutes. Panel f shows ion chromatogram at RT 1.49 minutes, most abundant species at *m/z* 321.1 of unknown metabolite DM-AM3. Panel g shows ion chromatogram at RT 2.05 minutes, most abundant species at *m/z* 267.2 and 305.1 of metabolite DM-AM1 $[M+H]^+$ and $[M+K]^+$, respectively

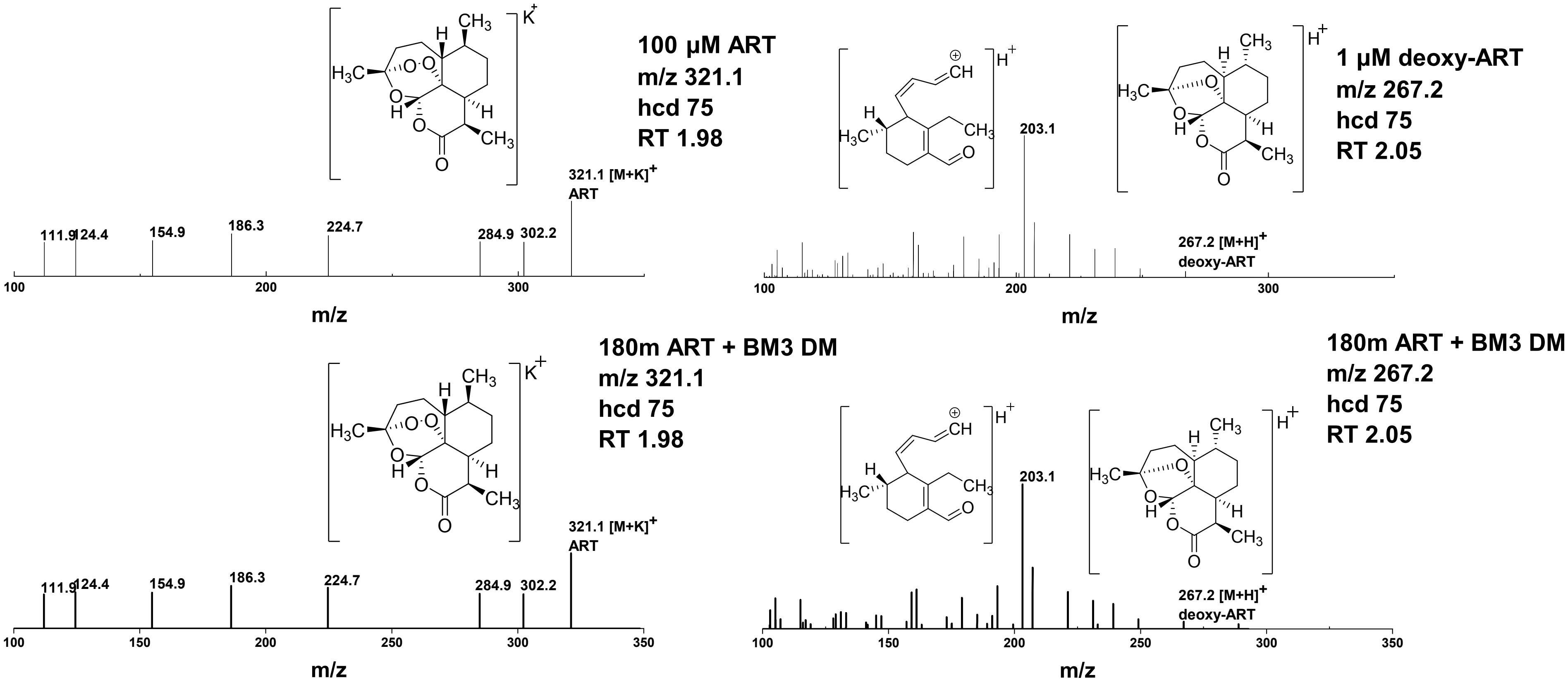

Figure S6: ESI (+) MS/MS fragmentation patterns of BM3 DM turnover of artemisinin at 180 minutes for parent (ART) and its main metabolite (deoxyART). RTs are related to peak chromatograms in Figure S1.

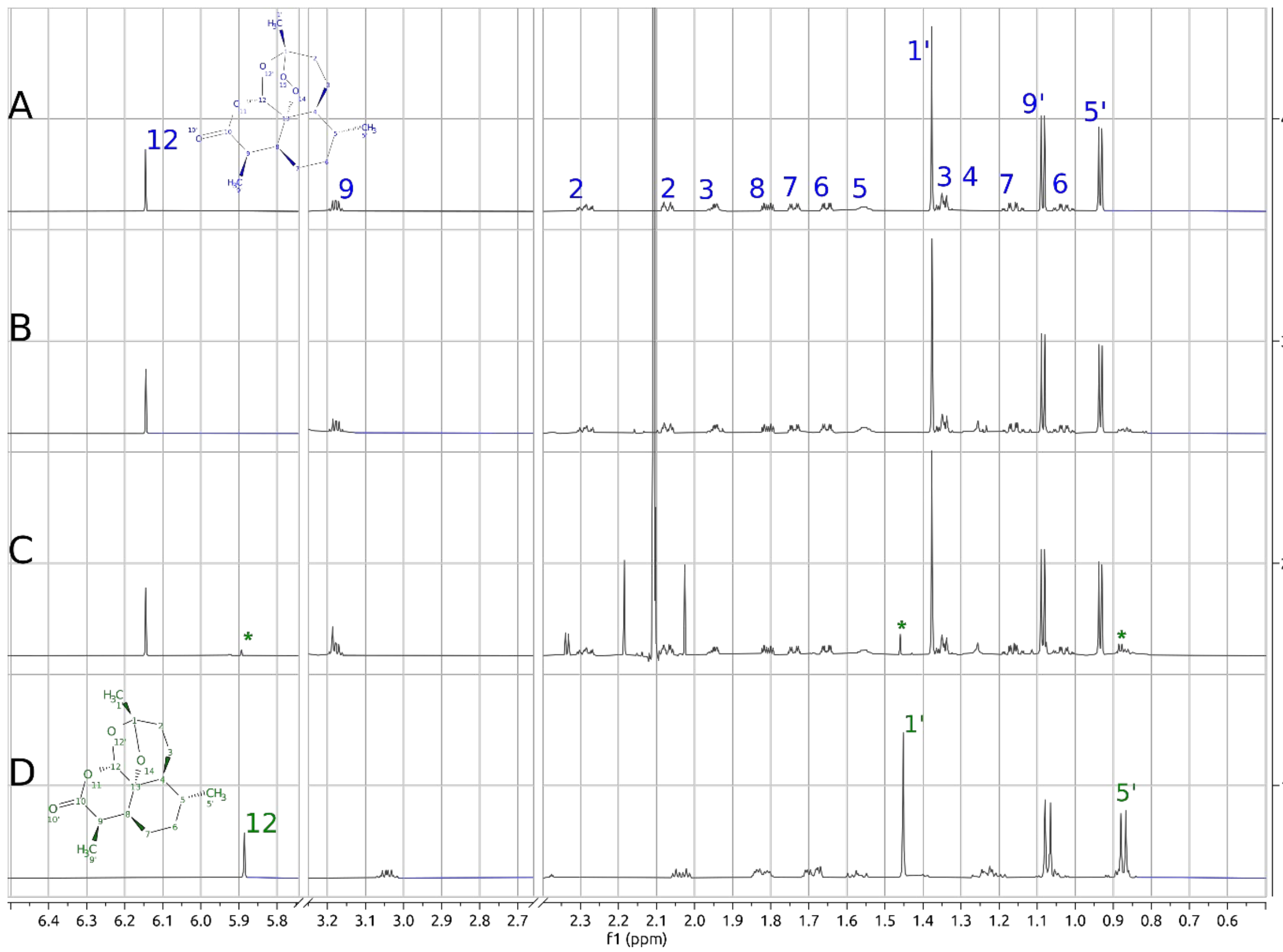

Figure S7: NMR of artemisinin and the metabolites derived from BM3 DM. ^{1}H NMR spectra of artemisinin (**A**) and deoxyartemisinin (**D**) standards and artemisinin in assay mix before (**B**) and 40 (**C**) minutes after the addition of DM BM3 P450. Blue shows artemesininin assignments taken from Zhu & Cook 2012 (confirmed by HSQC and COSY) and dark green show peaks arising from deoxyartemesinin. Spectrum in **C** shows accumulation ~10% metabolite. **A**, **B** & **C** recorded at 800MHz and **D** at 400 MHz.

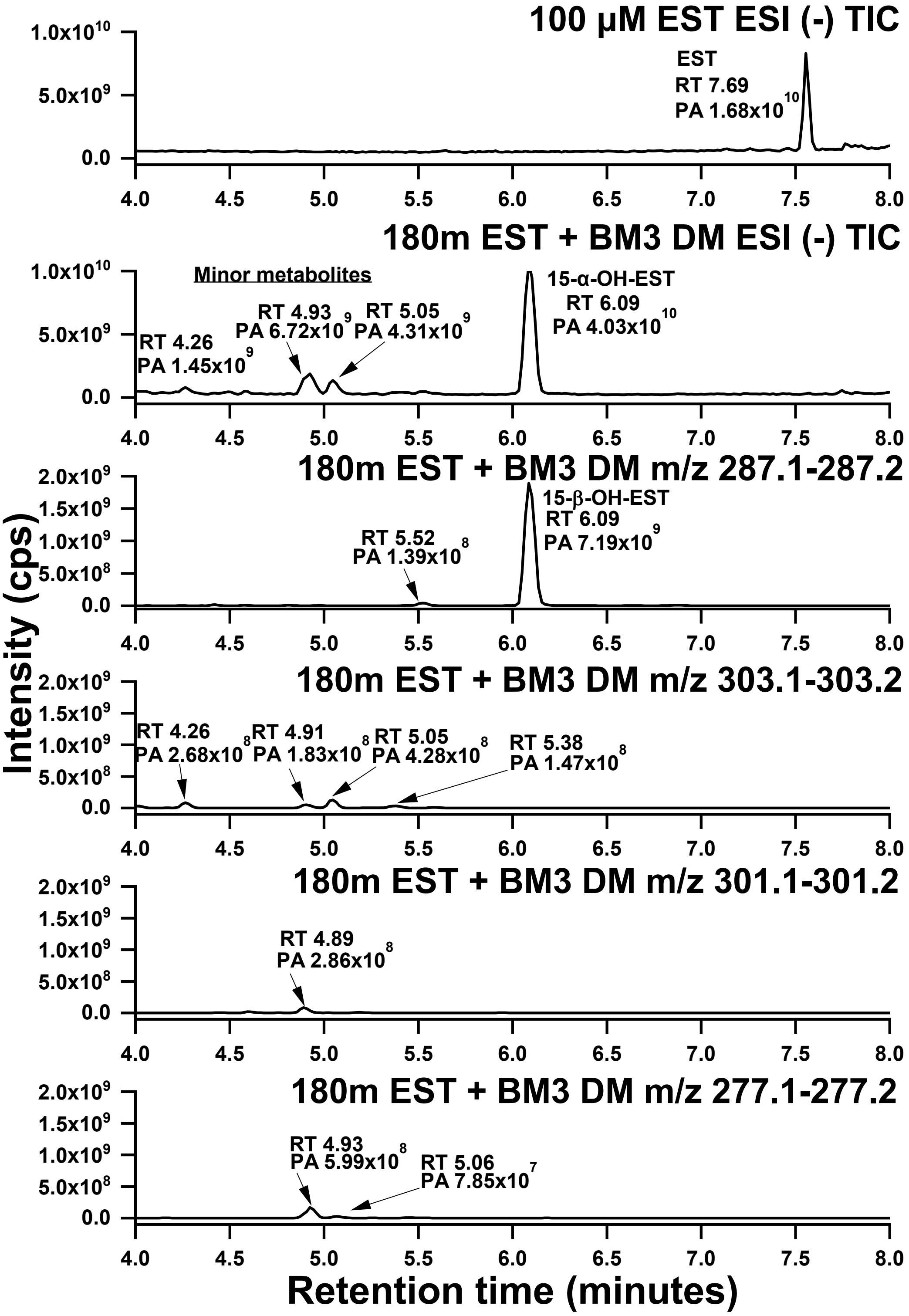

Figure S8: ESI (-) LCMS/MS peak chromatogram of BM3 DM turnover of estradiol (EST) at 180 minutes, significant metabolites RTs and peak areas indicated. EST standard is also displayed.

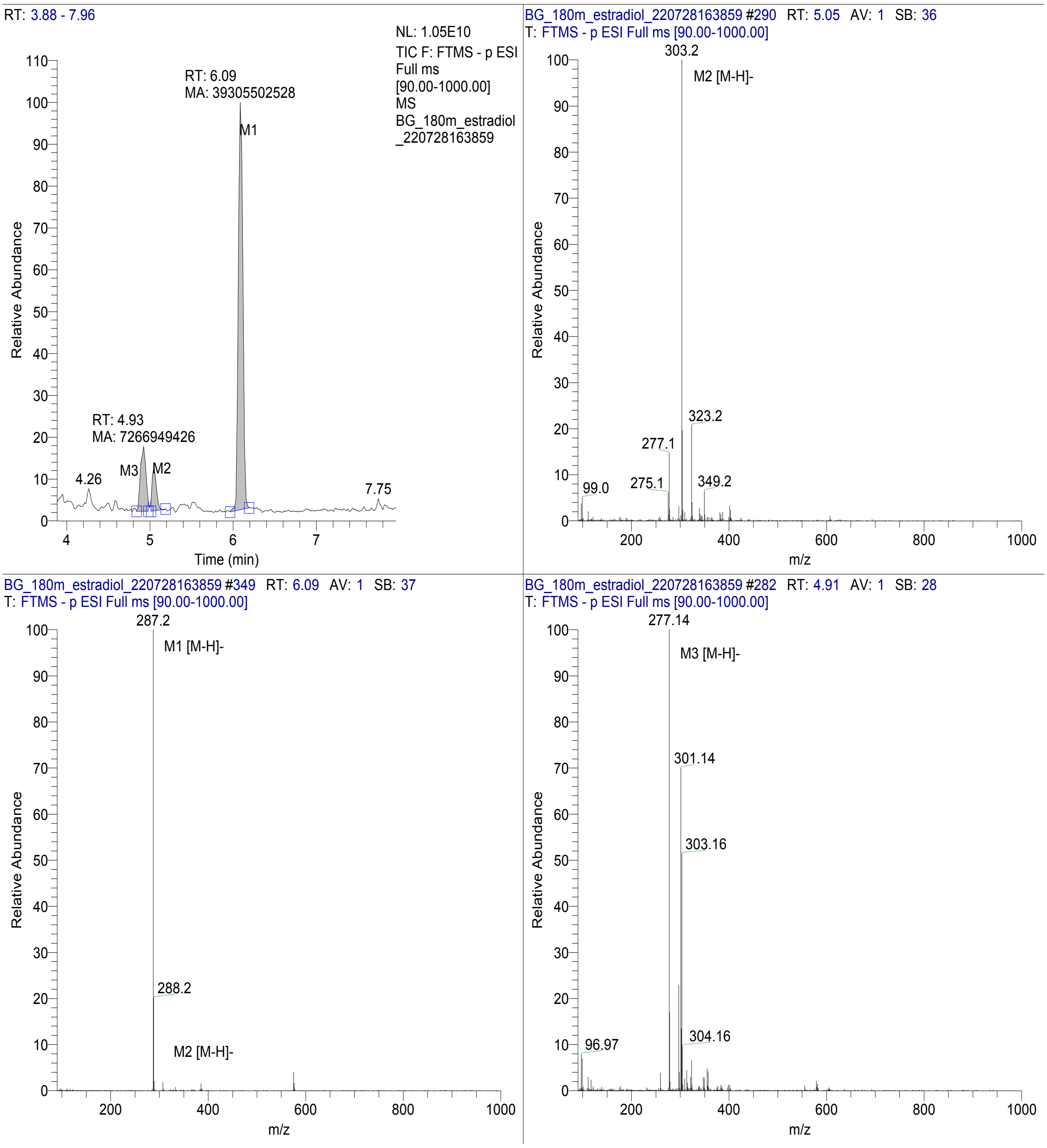

Figure S9: LC-MS/MS chromatogram of the 180 minute timepoint of incubation EST + BM3 DM. Top left panel shows peak chromatogram of 180 minute timepoint with RTs and peak areas (MA) indicated for significant metabolites. Metabolites are labelled M1, M2, and M3. Bottom left panel shows ion chromatogram at RT 6.09 minutes, most abundant species at *m/z* 287.2 is indicative of major DM-EM1 ($[M-H]^-$) monohydroxylated metabolite species. Top right panel shows ion chromatogram at RT 5.05 minutes, most abundant species at *m/z* 303.2 is indicative of minor DM-EM2 ($[M-H]^-$) dihydroxylated metabolite species. Bottom right panel shows ion chromatogram at RT 4.91 minutes, three species shown at *m/z* 277.1, 301.1, 303.2; 303.2 is indicative of minor DM-EM3 ($[M-H]^-$) dihydroxylated metabolite species.

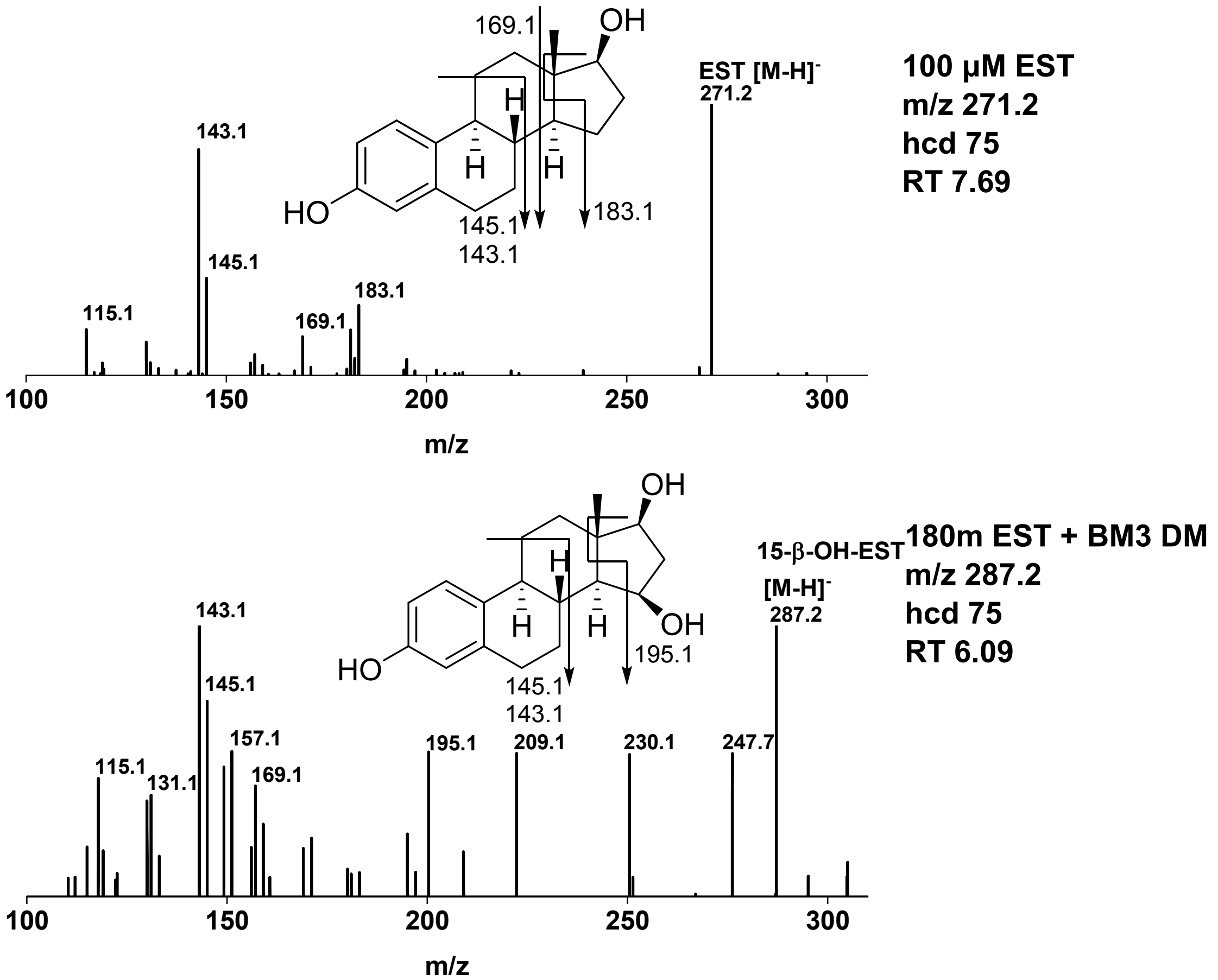

Figure S10: **ESI (-) MS/MS fragmentation patterns of estradiol (EST) standard and EST + BM3 DM turnover at 180 minutes for main metabolite 15-β-OH-EST.** RTs are related to peak chromatograms in Figure S3.

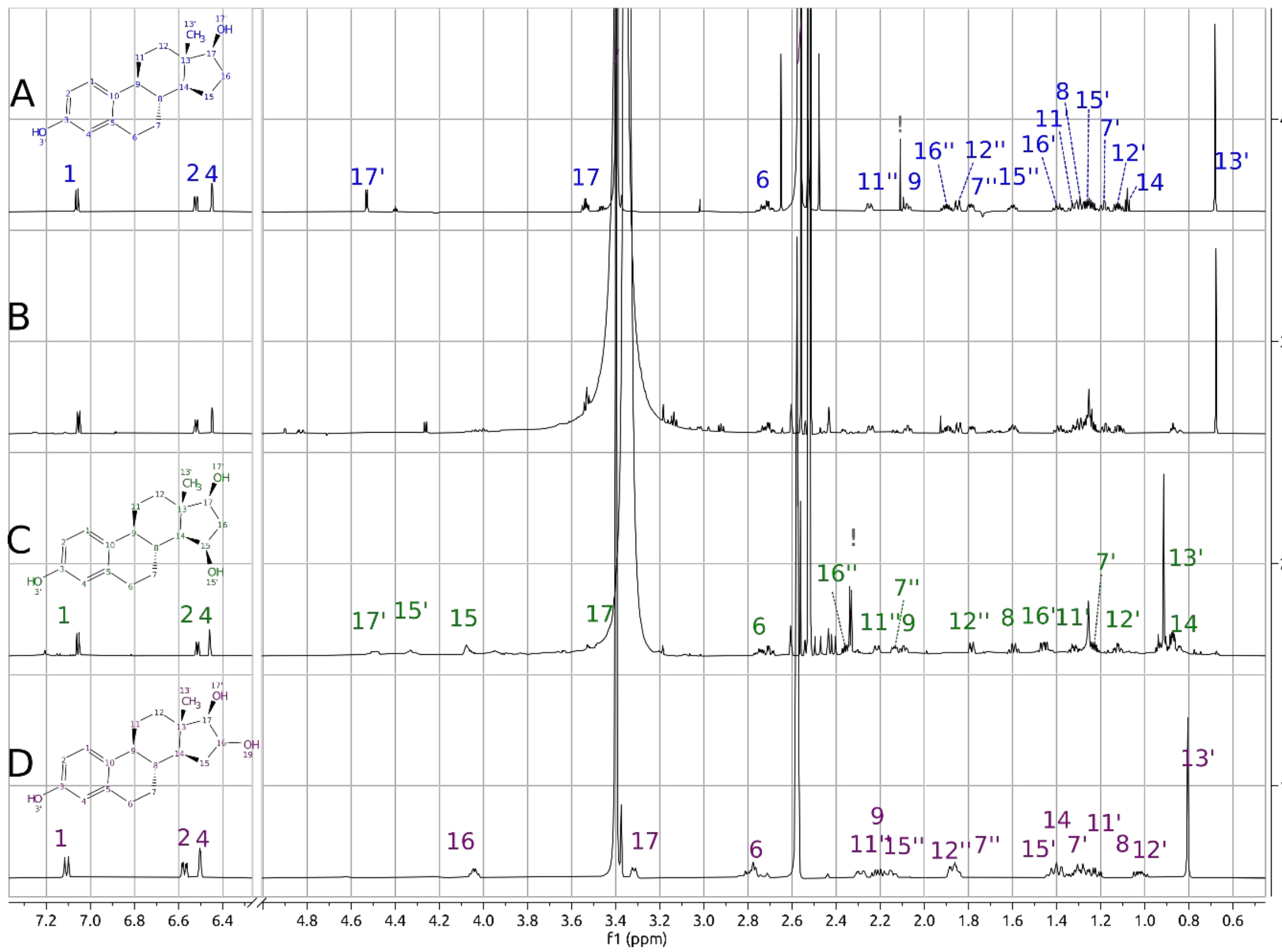

Figure S11: **NMR of estradiol and the metabolites derived from BM3 DM.** ^{1}H NMR spectra of β-estradiol (**A**) and 16 β-hydroxyestradiol (**D**) standards and β-estradiol in assay mix before (**B**) and 180 (**C**) minutes after the addition of DM BM3 P450. Blue shows β-estradiol assignments taken from HMDB0000151 (confirmed by HSQC and COSY), dark green show assignments arising from estra 3, β15, β17-triol, assigned in Figure S7, S8, S9 and purple shows assignment of estriol (HMDB0000153), not present in the product spectrum. Blue shows β-estradiolβ-estradiol assignments taken from HMDB0000151 (confirmed by HSQC and COSY), dark green show assignments arising from estra 3, β15, β17-triol, assigned in Figure S7, S8, S9 and purple shows assignment of estriol (HMDB0000153), not present in the product spectrum. Spectrum in **C** shows accumulation >90% metabolite. Purple **A**, **B** & **C** recorded at 800MHz and **D** at 400 MHz.

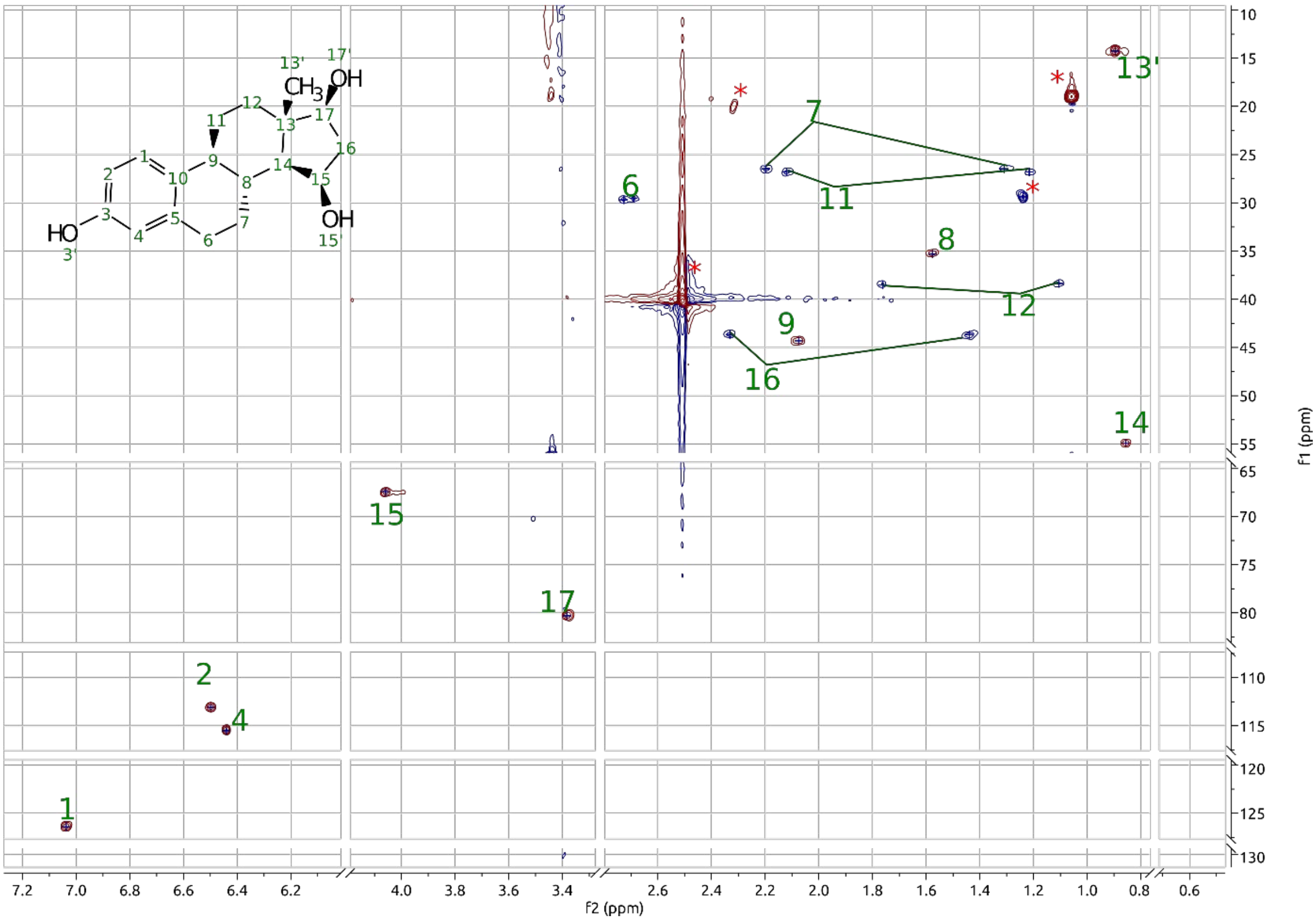

Figure S12: NMR elucidation of 15-β hydroxy-estradiol. ^{13}C-^{1}H multiplicity edited HSQC spectrum of the 180m estradiol reaction sample recorded at 800MHz. Correlations from the 15-β hydroxy-estradiol are labelled in green. Impurities are labelled with red asterisks. δH (800 MHz, DMSO-d6) NMR: 9.94 (s, 1H, C3OH), 7.05 (d, J = 8.5 Hz, 1H, C1H), 6.52 (dd, J = 8.4, 2.7 Hz, 1H, C2H), 6.46 (d, J = 2.6 Hz, 1H, C4H), 4.51 (d, J = 5.0 Hz, 1H, C17Oa), 4.34 (d, J = 4.0 Hz, 1H), 4.07 (td, J = 6.6, 3.2 Hz, 1H), 2.74 (ddd, J = 18.0, 12.1, 6.4 Hz, 1H, C6Hα), 2.69 (dd, J = 16.7, 5.8 Hz, 1H, C6Hβ), 2.37 – 2.34 (m, 1H, C16Hβ), 2.21 (dd, J = 13.5, 3.7 Hz, 1H, C11Hα), 2.13 (ddt, J = 11.8, 5.7, 2.3 Hz, 1H, C7Hβ), 2.09 (td, J = 11.5, 4.1 Hz, 1H, C9H), 1.78 (dt, J = 12.4, 3.3 Hz, 1H, C12Hβ), 1.59 (qd, J = 11.2, 2.6 Hz, 1H, C8H), 1.45 (ddd, J = 13.9, 8.9, 2.6 Hz, 1H, C16Hα), 1.31 (qd, J = 13.1, 3.9 Hz, 1H, C11Hβ), 1.22 (ddt, J = 18.2, 12.1, 5.5 Hz, 1H, C7Hα), 1.12 (td, J = 12.9, 4.0 Hz, 1H, C12Hα), 0.91 (s, 3H, C13'H3), 0.89 – 0.85 (m, 1H, C14H).

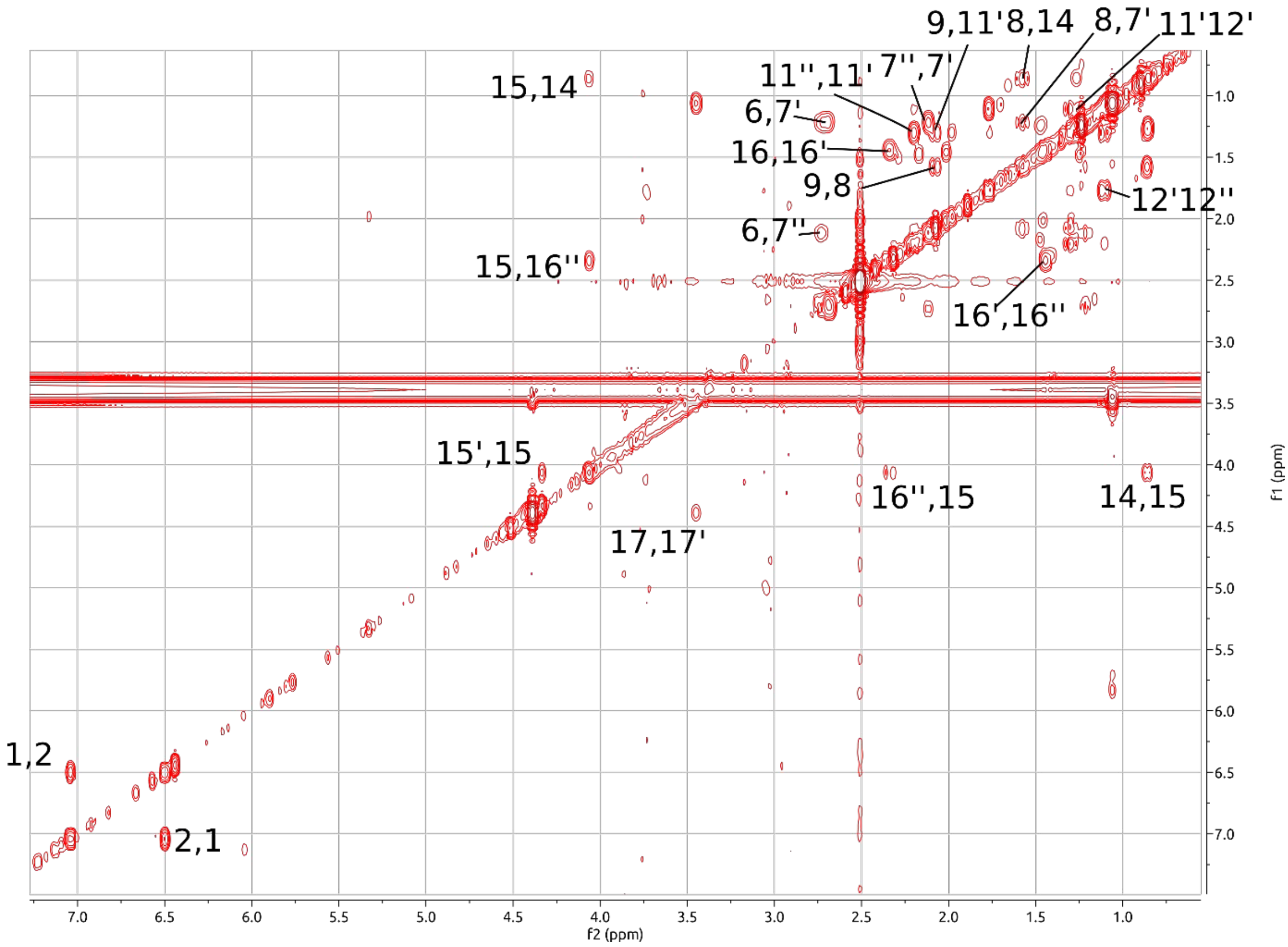

Figure S13: NMR elucidation of 15-β hydroxy-estradiol. ^{1}H-^{1}H-COSY spectrum (800 MHz, DMSO-d6) recorded on the 180m estradiol reaction sample. Correlations from the 15-β hydroxy-estradiol are labelled in black.

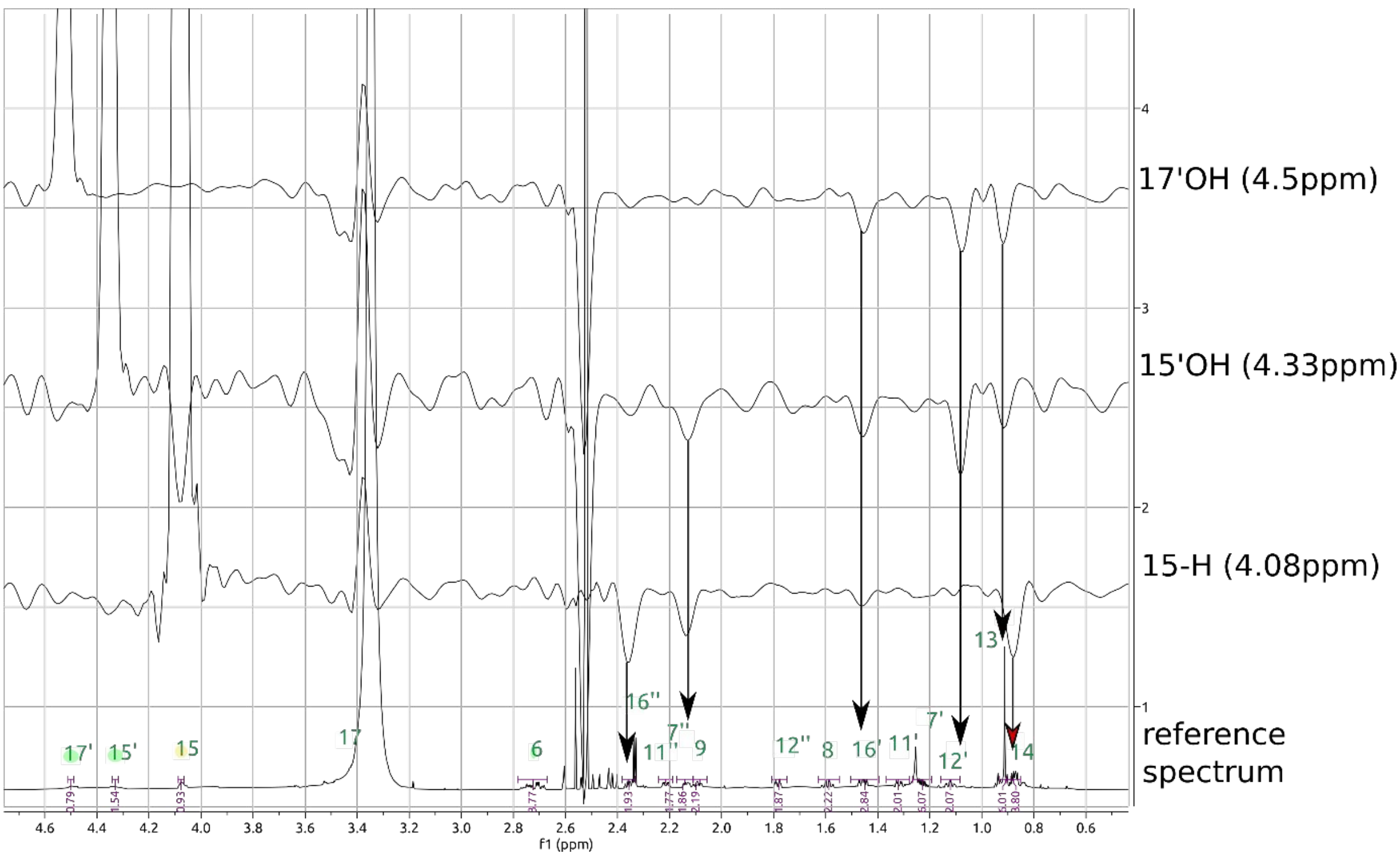

Figure S14: NMR elucidation of 15-β hydroxy-estradiol. Slices from ^{1}H-^{1}H ROESY (800 MHz, DMSO-d6) spectrum recorded on the 180m estradiol reaction sample, corresponding to the 17'OH, 15'OH and 15H resonances. The through-space correlations for the 17' and 15' OH are very similar, indicating they are on the same face of the molecule, with strong correlations to the 13' methyl, and the 16' (beta)- proton. The 15H proton has strong correlations to the 14 and 16''(alpha) protons, indicating that 14H, 15H and 16''H are all on the same face of the molecule, identifying the product as β-15 hydroxy-estradiol.

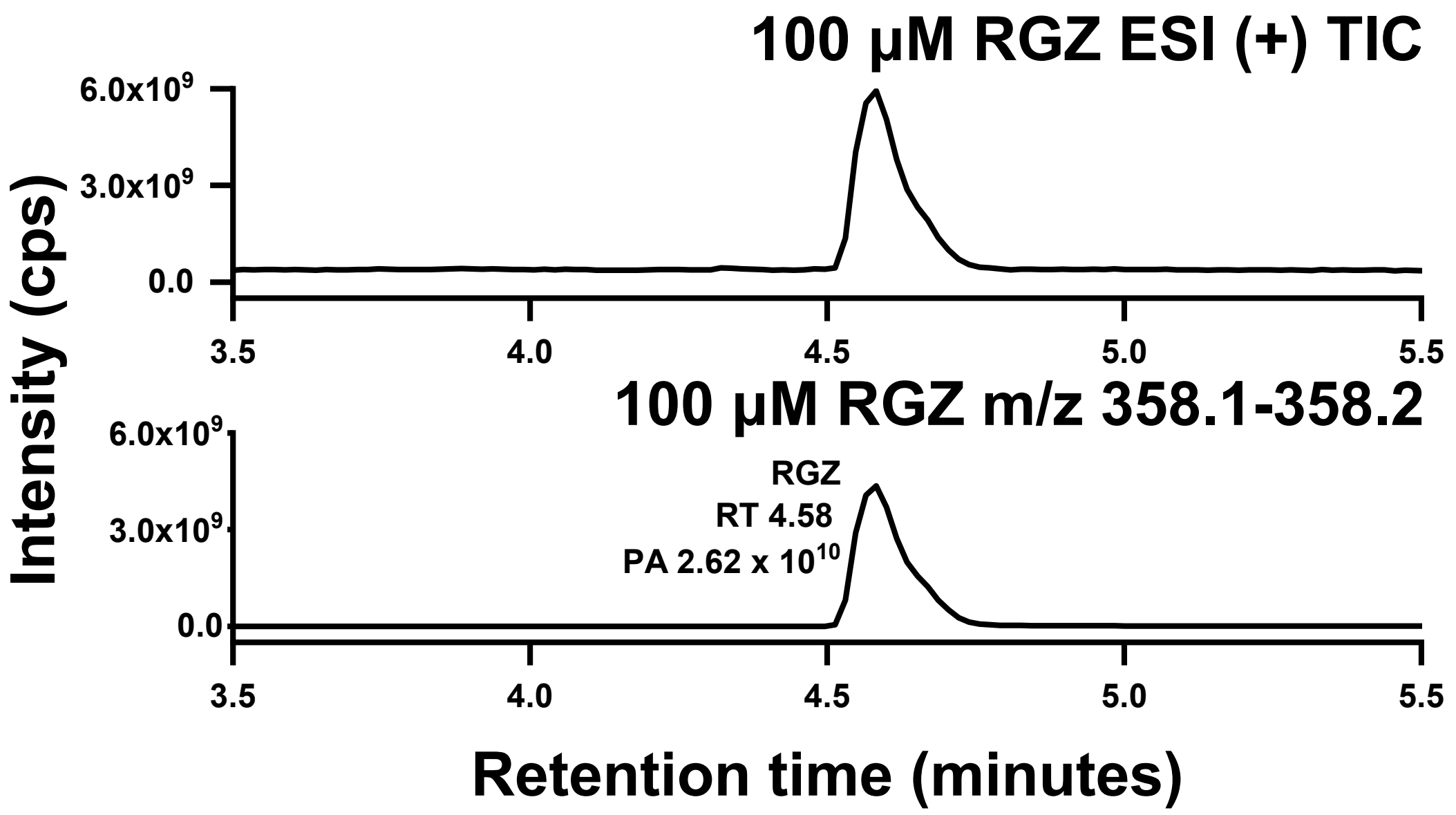

Figure S15: **ESI (+) LCMS/MS peak chromatogram of 100 μM rosiglitazone (RGZ).** TIC displayed and mass windows (±0.1 Da) displayed.

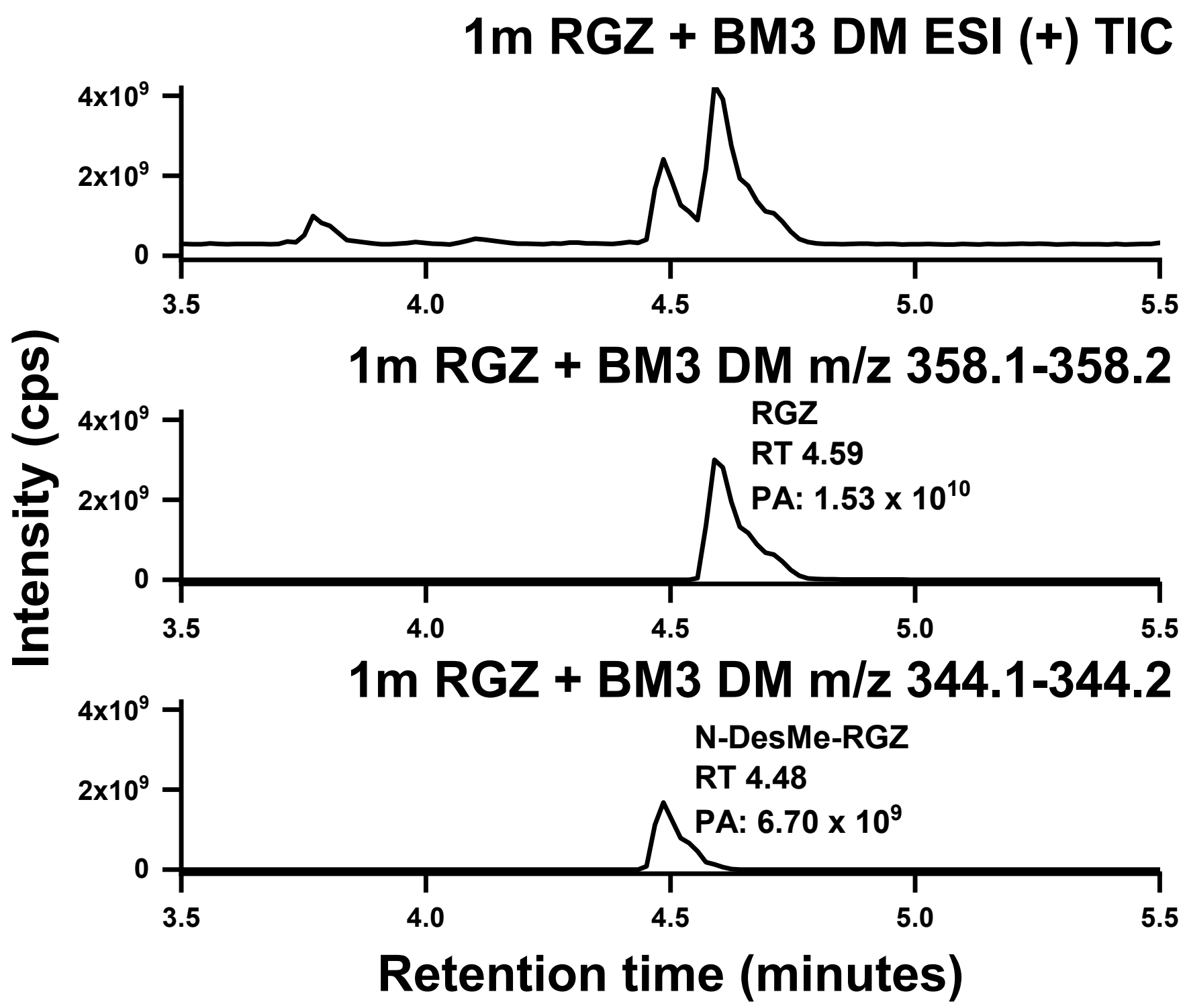

Figure S16: **ESI (+) LCMS/MS peak chromatogram of RGZ + BM3 DM turnover at 1 minute.** TIC displayed and mass windows (±0.1 Da) displayed for all major metabolites.

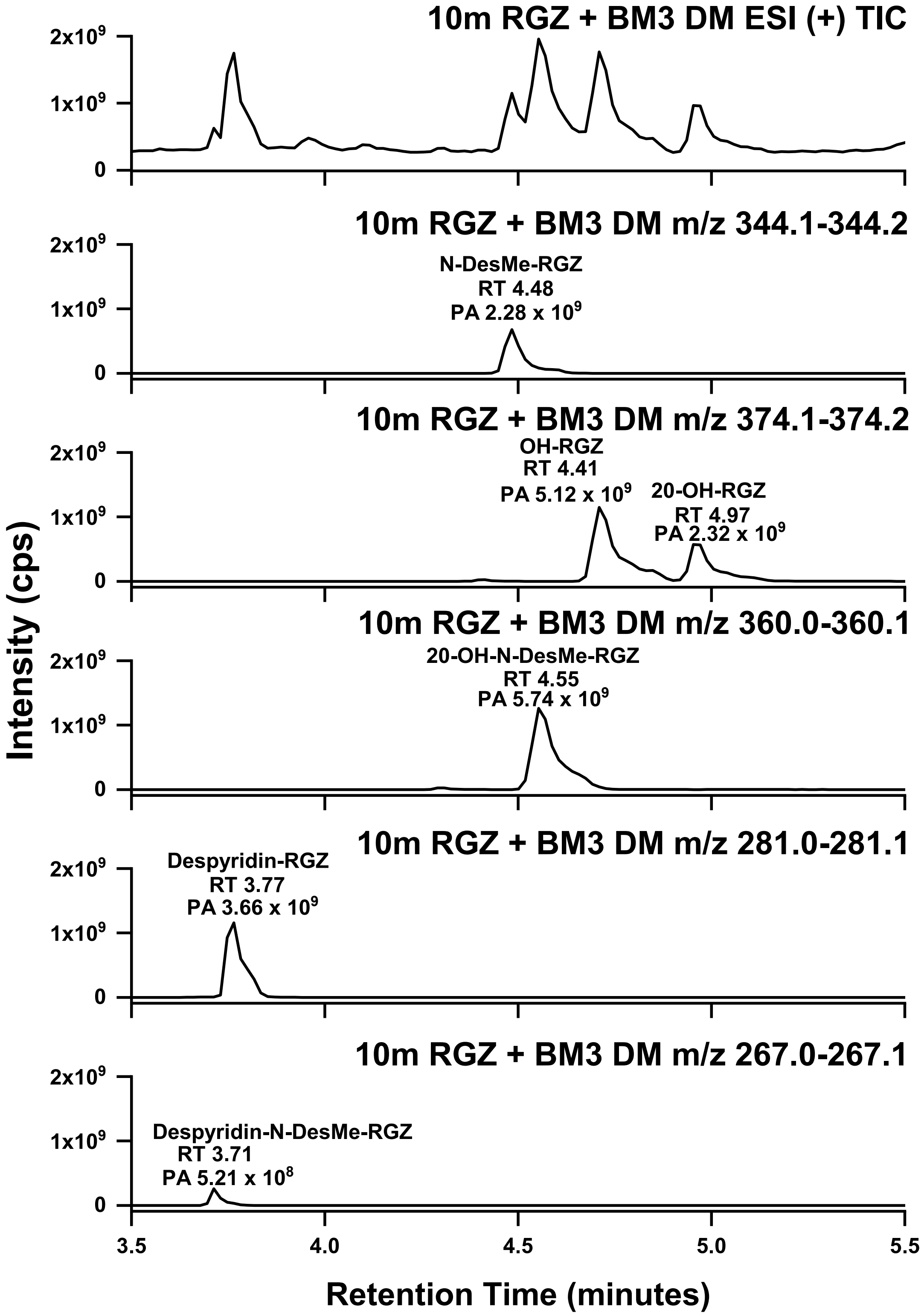

Figure S17: ESI (+) LCMS/MS peak chromatogram of RGZ + BM3 DM turnover at 10 minutes. TIC displayed and mass windows (±0.1 Da) displayed for all major metabolites.

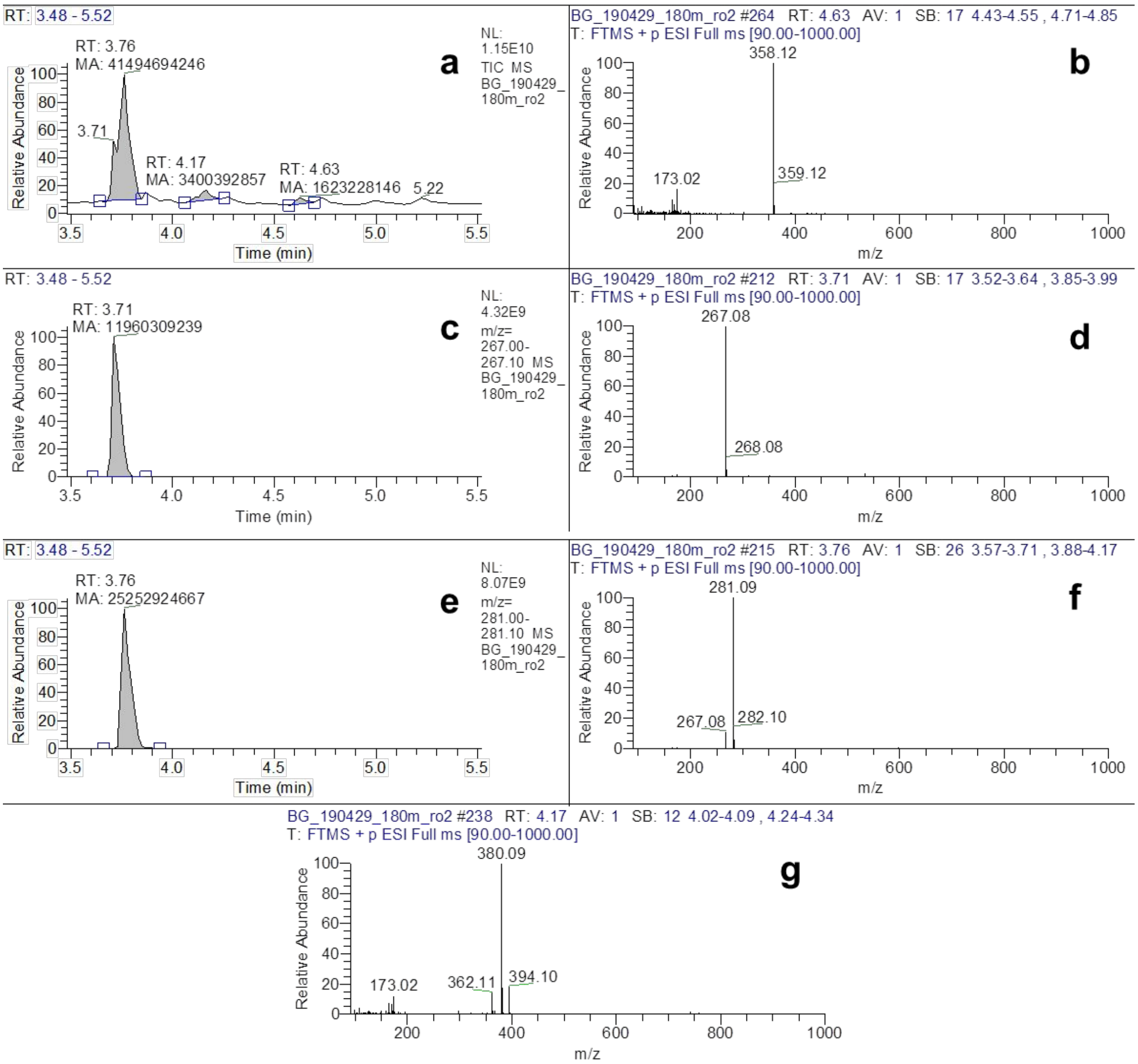

Figure S18: LC-MS/MS chromatogram of the 180 minute timepoint of RGZ + BM3 DM incubation. Panel a shows TIC peak chromatogram with RTs and peak areas (MA) indicated for significant metabolites. Panel b shows ion chromatogram at RT 4.63 minutes, most abundant species at *m/z* 358.1 is indicative of RGZ $[M+H]^+$. Panel c shows peak chromatogram at *m/z* 267.0-267.1, peak at RT 3.71 is indicative of DM-RoM7 unknown metabolite species. Panel d shows ion chromatogram at RT 3.71 minutes, most abundant species at *m/z* 267.1 is indicative of unknown metabolite species DM-RoM7. Panel e shows peak chromatogram at *m/z* 281.0-281.1, peak at RT 3.76 minutes is indicative of unknown metabolite species DM-RoM6. Panel f shows ion chromatogram at RT 3.76 minutes, most abundant species at *m/z* 281.1 is indicative of unknown metabolite species DM-RoM6. Panel g shows ion chromatogram at RT 4.17 minutes, most abundant species at *m/z* 380.9 is indicative of unknown metabolite species DM-RoM9.

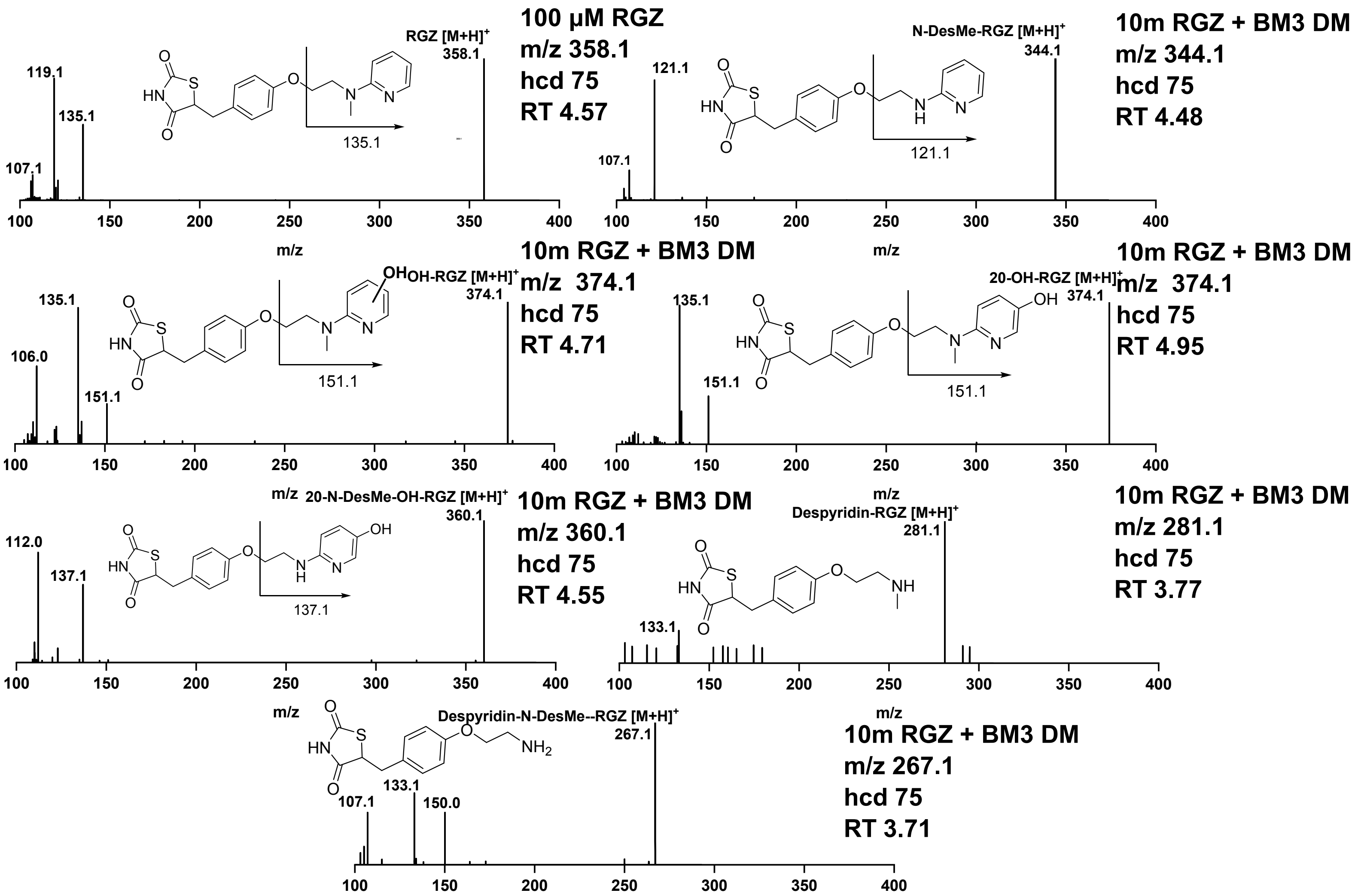

Figure S19: **ESI (+) MS/MS fragmentation patterns of RGZ + BM3 DM turnover at 10 minutes for major metabolites.** RTs are related to peak chromatograms in Figure S12.

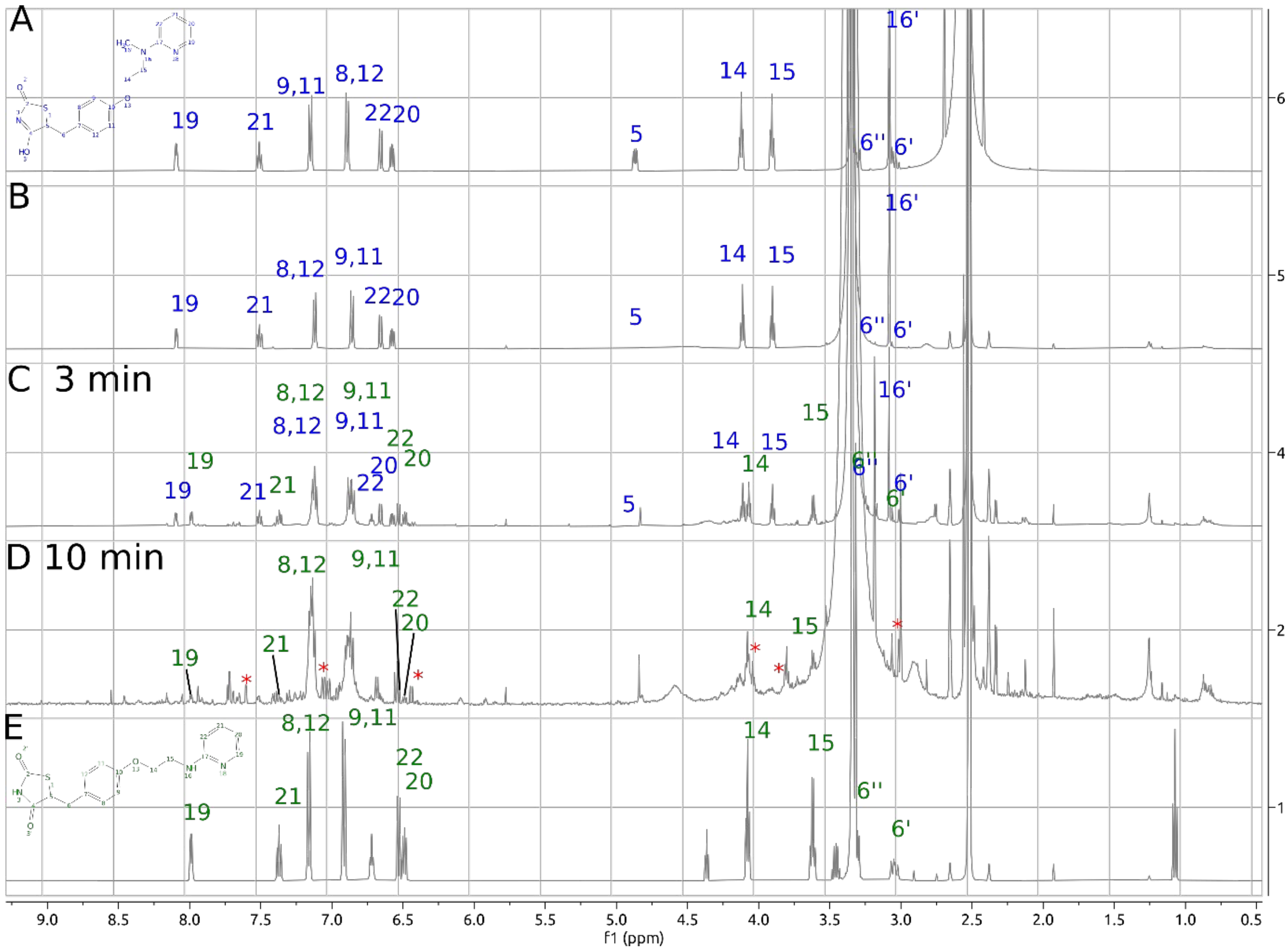

Figure S20: NMR of rosiglitazone and the metabolites derived from BM3 DM. ^{1}H NMR spectra (500MHz, DMSO-d6)) of rosiglitazone (**A**) and desmethylrosiglitazone (**E**) standards and 100 µM rosiglitazone, extracted from assay mix before (**B**) and 1 (**C**) and 10 minutes (**D**) after the addition of DM BM3 P450. Rosiglitazone peaks are indicated by blue assignments and desmethylrosiglitazone peaks are indicated by green assignments. Red asterisks are associated with additional metabolites. Rosiglitazone assignment is based on Figure S13 and S14, and desmethylrosiglitazone by comparison with this assignment. The thiazolium ring undergoes tautomerisation and exchange broadens the 2H resonance.

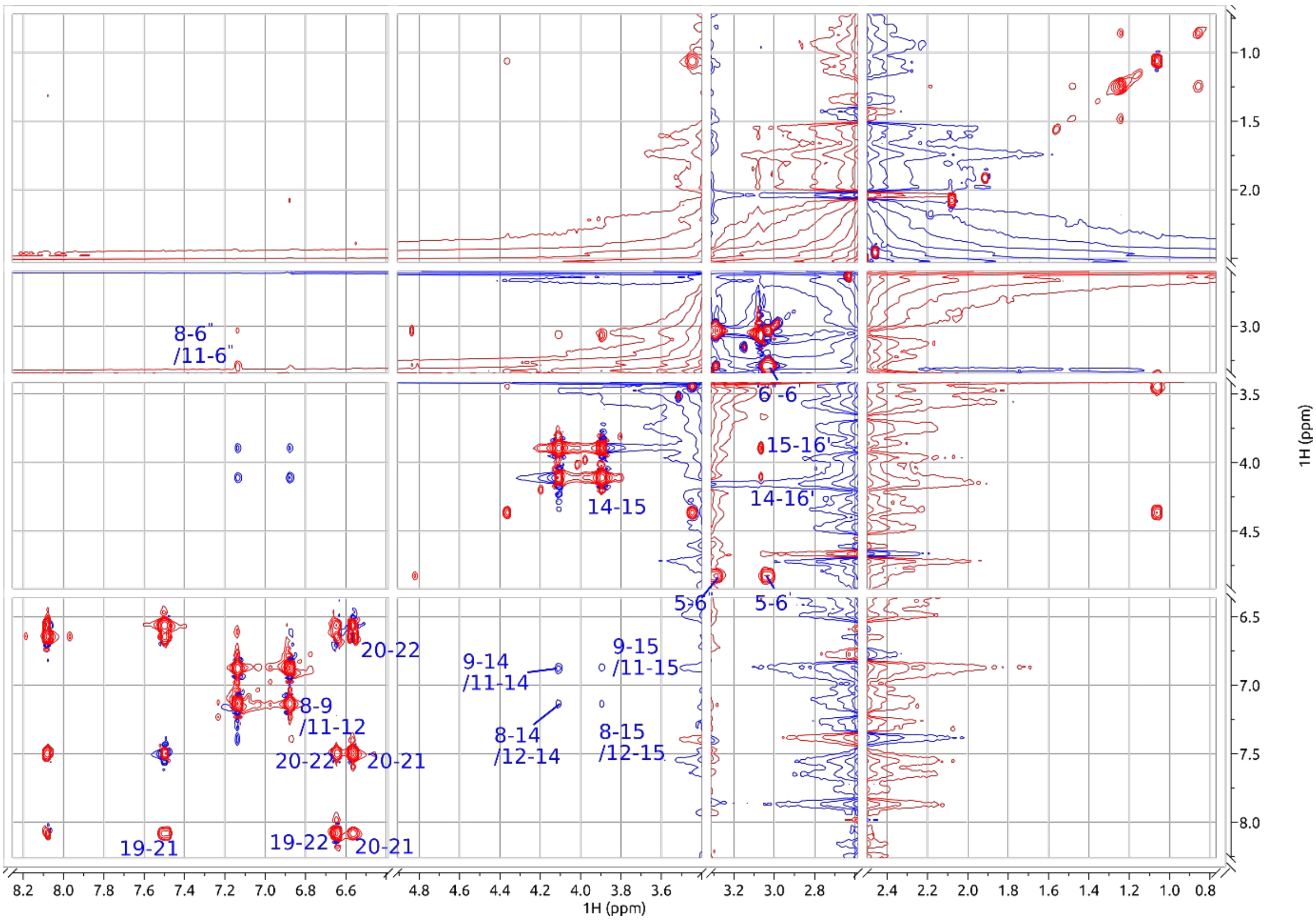

Figure S21: NMR elucidation of N-despyridinyl rosiglitazone. $^{1}H^{1}H$ TOSCY NMR spectrum of rosiglitazone recorded at 500MHz om DMSO-d6 with assignments labelled in blue.

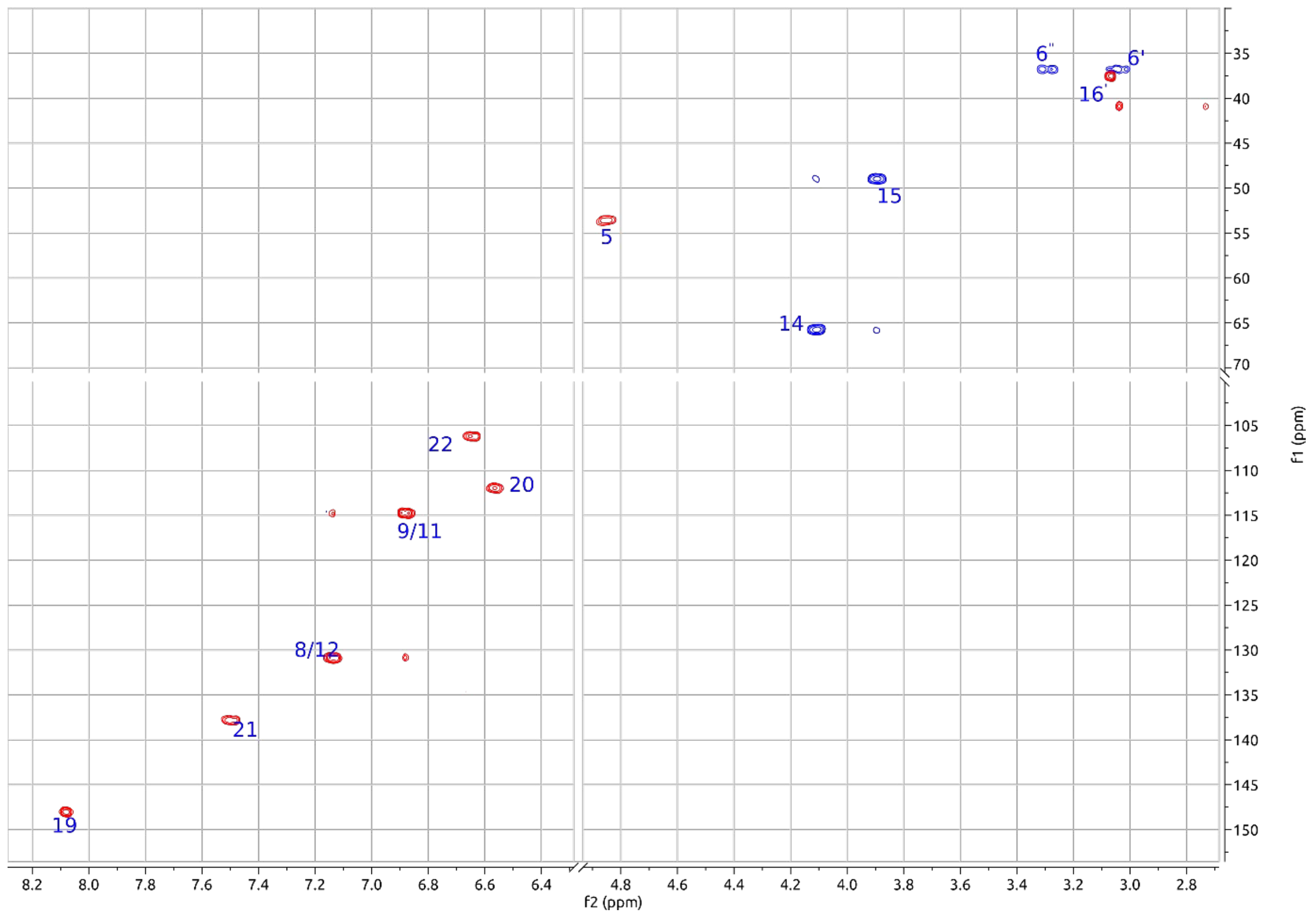

Figure S22: NMR elucidation of N-despyridinyl rosiglitazone. $^{1}H^{13}C$ multiplicity-edited HSQC NMR spectrum of rosiglitazone recorded at 500MHz with assignments labelled in blue. δH (500 MHz, DMSO-d6) 8.09 (ddd, J = 4.9, 2.0, 0.9 Hz, 1H, C19H), 7.51 (ddd, J = 8.8, 7.1, 2.0 Hz, 1H, C21H), 7.16 – 7.06 (m, 2H, C8H, C12H), 6.92 – 6.80 (m, 3H, C9H, C11H), 6.66 (dd, J = 8.6, 1.0 Hz, 1H, C22H), 6.57 (ddd, J = 7.1, 4.9, 0.9 Hz, 1H, C20H), 4.11 (t, J = 6.0 Hz, 2H, C14H2), 3.90 (t, J = 5.9 Hz, 2H, C15H2), 3.19 (d, J = 5.3 Hz, 2H, C6H2), 3.08 (s, 3H, C27H3).

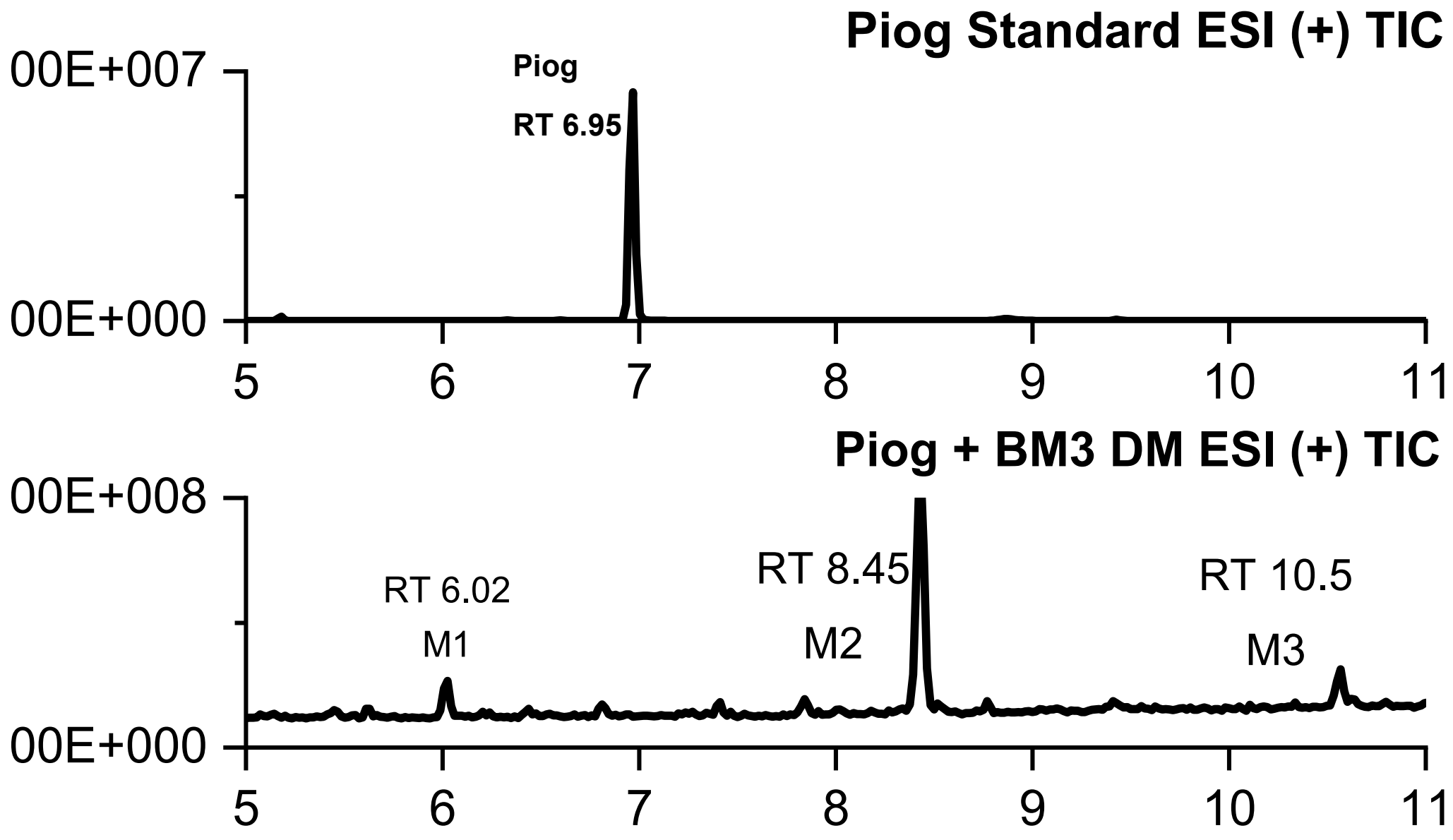

Figure S23: **Pioglitazone (Piog) peak chromatogram of piog standard and 20 minute piog + BM3 DM.** Retention time and literature identification showed for all major metabolites.

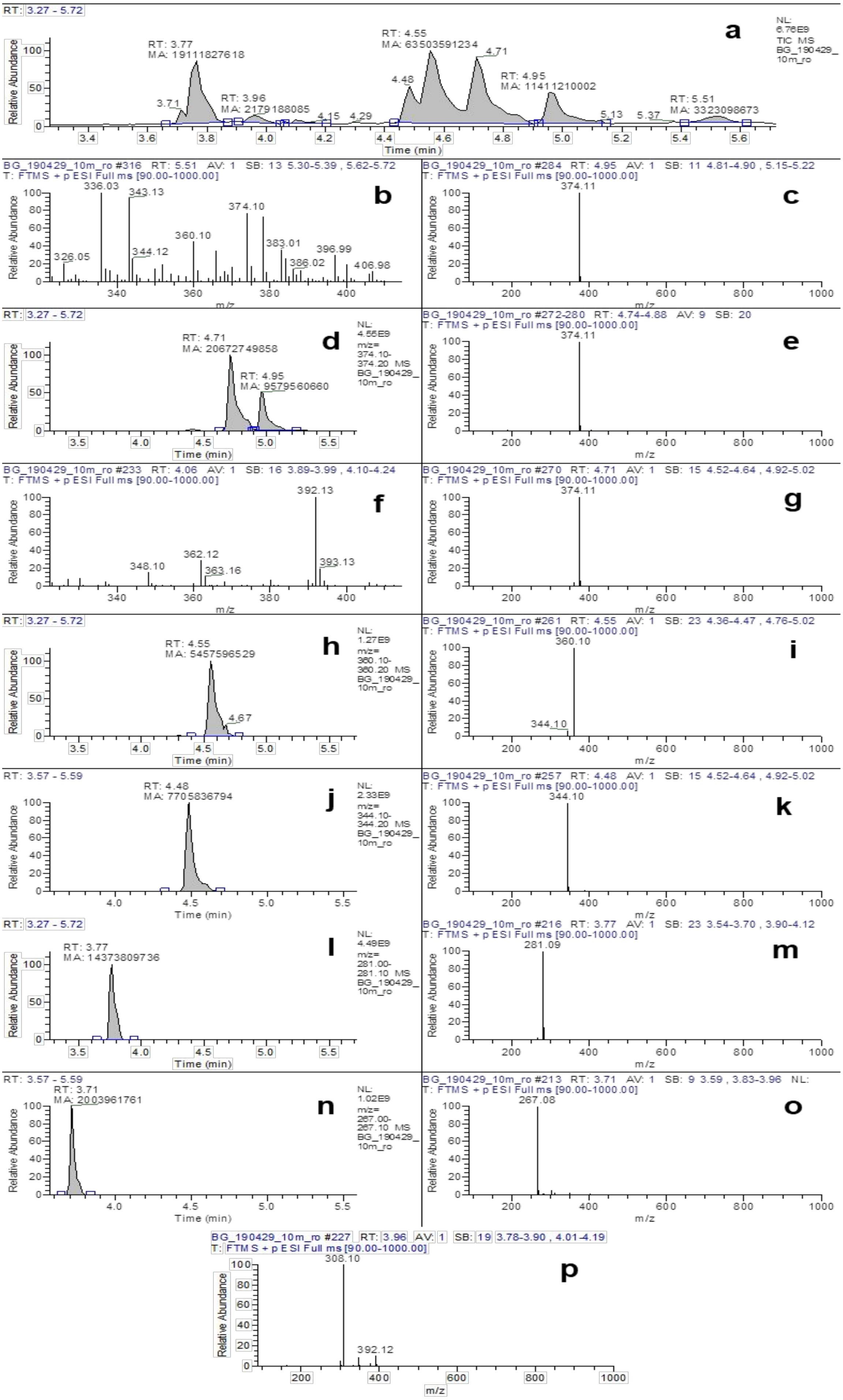

a
RT: 3.27 - 5.72
NL: 6.76E9 TIC MS BG_190429_10m_ro
RT: 3.77 MA: 19111827618
RT: 3.96 MA: 2179188085
RT: 4.55 MA: 63503591234
RT: 4.95 MA: 11411210002
RT: 5.51 MA: 3323098673
Time (min)
Relative Abundance
b
BG_190429_10m_ro #316 RT: 5.51 AV: 1 SB: 13 5.30-5.39 , 5.62-5.72
T: FTMS + p ESI Full ms [90.00-1000.00]
336.03
343.13
374.10
360.10
383.01
396.99
406.98
m/z
c
BG_190429_10m_ro #284 RT: 4.95 AV: 1 SB: 11 4.81-4.90 , 5.15-5.22
T: FTMS + p ESI Full ms [90.00-1000.00]
374.11
d
RT: 3.27 - 5.72
NL: 4.56E9 m/z= 374.10-374.20 MS BG_190429_10m_ro
RT: 4.71 MA: 20672749858
RT: 4.95 MA: 9579560660
e
BG_190429_10m_ro #272-280 RT: 4.74-4.88 AV: 9 SB: 20
T: FTMS + p ESI Full ms [90.00-1000.00]
374.11
f
BG_190429_10m_ro #233 RT: 4.06 AV: 1 SB: 16 3.89-3.99 , 4.10-4.24
T: FTMS + p ESI Full ms [90.00-1000.00]
392.13
362.12
348.10
363.16
393.13
g
BG_190429_10m_ro #270 RT: 4.71 AV: 1 SB: 15 4.52-4.64 , 4.92-5.02
T: FTMS + p ESI Full ms [90.00-1000.00]
374.11
h
RT: 3.27 - 5.72
NL: 1.27E9 m/z= 360.10-360.20 MS BG_190429_10m_ro
RT: 4.55 MA: 5457596529
4.67
i
BG_190429_10m_ro #261 RT: 4.55 AV: 1 SB: 23 4.36-4.47 , 4.76-5.02
T: FTMS + p ESI Full ms [90.00-1000.00]
360.10
344.10
j
RT: 3.57 - 5.59
NL: 2.33E9 m/z= 344.10-344.20 MS BG_190429_10m_ro
RT: 4.48 MA: 7705836794
k
BG_190429_10m_ro #257 RT: 4.48 AV: 1 SB: 15 4.52-4.64 , 4.92-5.02
T: FTMS + p ESI Full ms [90.00-1000.00]
344.10
l
RT: 3.27 - 5.72
NL: 4.49E9 m/z= 281.00-281.10 MS BG_190429_10m_ro
RT: 3.77 MA: 14373809736
m
BG_190429_10m_ro #216 RT: 3.77 AV: 1 SB: 23 3.54-3.70 , 3.90-4.12
T: FTMS + p ESI Full ms [90.00-1000.00]
281.09
n
RT: 3.57 - 5.59
NL: 1.02E9 m/z= 267.00-267.10 MS BG_190429_10m_ro
RT: 3.71 MA: 2003961761
o
BG_190429_10m_ro #213 RT: 3.71 AV: 1 SB: 9 3.59 , 3.83-3.96 NL:
T: FTMS + p ESI Full ms [90.00-1000.00]
267.08
p
BG_190429_10m_ro #227 RT: 3.96 AV: 1 SB: 19 3.78-3.90 , 4.01-4.19
T: FTMS + p ESI Full ms [90.00-1000.00]
308.10
392.12
m/z

Figure S24: LC-MS/MS chromatogram of the 10 minute timepoint of RGZ + BM3 DM incubation. Panel a shows TIC peak chromatogram with RTs and peak areas (MA) indicated for significant metabolites. Panel b shows ion chromatogram at RT 5.51, several species observed indicating a mixture of metabolites. Panel c shows ion chromatogram at RT 4.95, most abundant species at *m/z* 374.1 is indicative of DM-RoM1. Panel d shows peak chromatogram at *m/z* 374.1-374.1 with RTs and peak areas indicated for DM-RoM4 (RT 4.71, $[M+H]^+$) and DM-RoM1 (RT 4.95 minutes, $[M+H]^+$). Panel e shows ion chromatogram at RT 4.95, most abundant species at *m/z* 374.1 is indicative of monohydroxylated metabolite DM-RoM8 $[M+H]^+$. Panel f shows ion chromatogram at RT 4.06, ions indicative of a mixture of unknown metabolite species. Panel g shows ion chromatogram at RT 4.71, most abundant species at *m/z* 374.1 is indicative of monohydroxylated metabolite DM-RoM4 $[M+H]^+$. Panel h shows peak chromatogram at *m/z* 360.1-360.2, peak at RT 4.55 minutes is indicative of N-demethylated monohydroxylated metabolite DM-RoM2 $[M+H]^+$. Panel i shows ion chromatogram at RT 4.55 minutes, most abundant species at *m/z* 360.1 is indicative of N-demethylated monohydroxylated metabolite species DM-RoM2 $[M+H]^+$. Panel j shows peak chromatogram at *m/z* 344.1-344.2, peak at RT 4.48 minutes is indicative of N-demethylated metabolite species DM-RoM3. Panel k shows ion chromatogram at 4.48 minutes, most abundant species at *m/z* 344.1 is indicative of N-demethylated metabolite species DM-RoM3 $[M+H]^+$. Panel l shows peak chromatogram at *m/z* 281.0-281.1, peak at RT 3.77 minutes is indicative of unknown metabolite species DM-RoM6. Panel m shows ion chromatogram at RT 3.77 minutes, most abundant species at *m/z* 281.1 is indicative of unknown metabolite species DM-RoM6 $[M+H]^+$. Panel n shows peak chromatogram at *m/z* 267.0-267.1, peak at RT 3.71 minutes is indicative of unknown metabolite species DM-RoM7. Panel o shows ion chromatogram at RT 3.71 minutes, most abundant species at *m/z* 267.1 is indicative of unknown metabolite species DM-RoM7 $[M+H]^+$. Panel p shows ion chromatogram at RT 3.96 minutes, peak at *m/z* 308.1 is indicative of unknown metabolite species DM-RoM8.

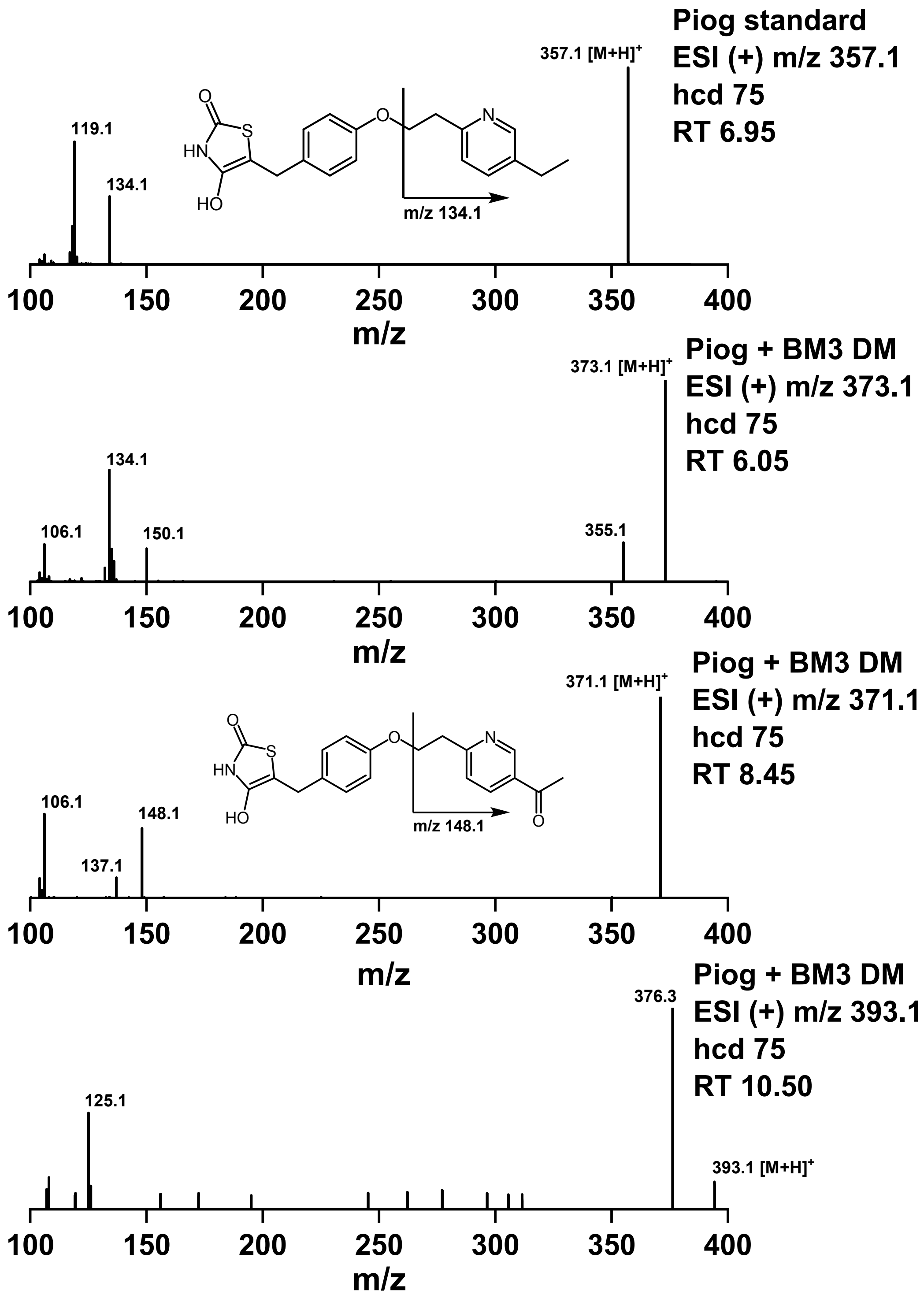

Figure S25: LCMS/MS fragmentations of pioglitazone standard and metabolites from 20 minute piog+ + BM3 DM. RTs are related to peak chromatograms in Figure S21.

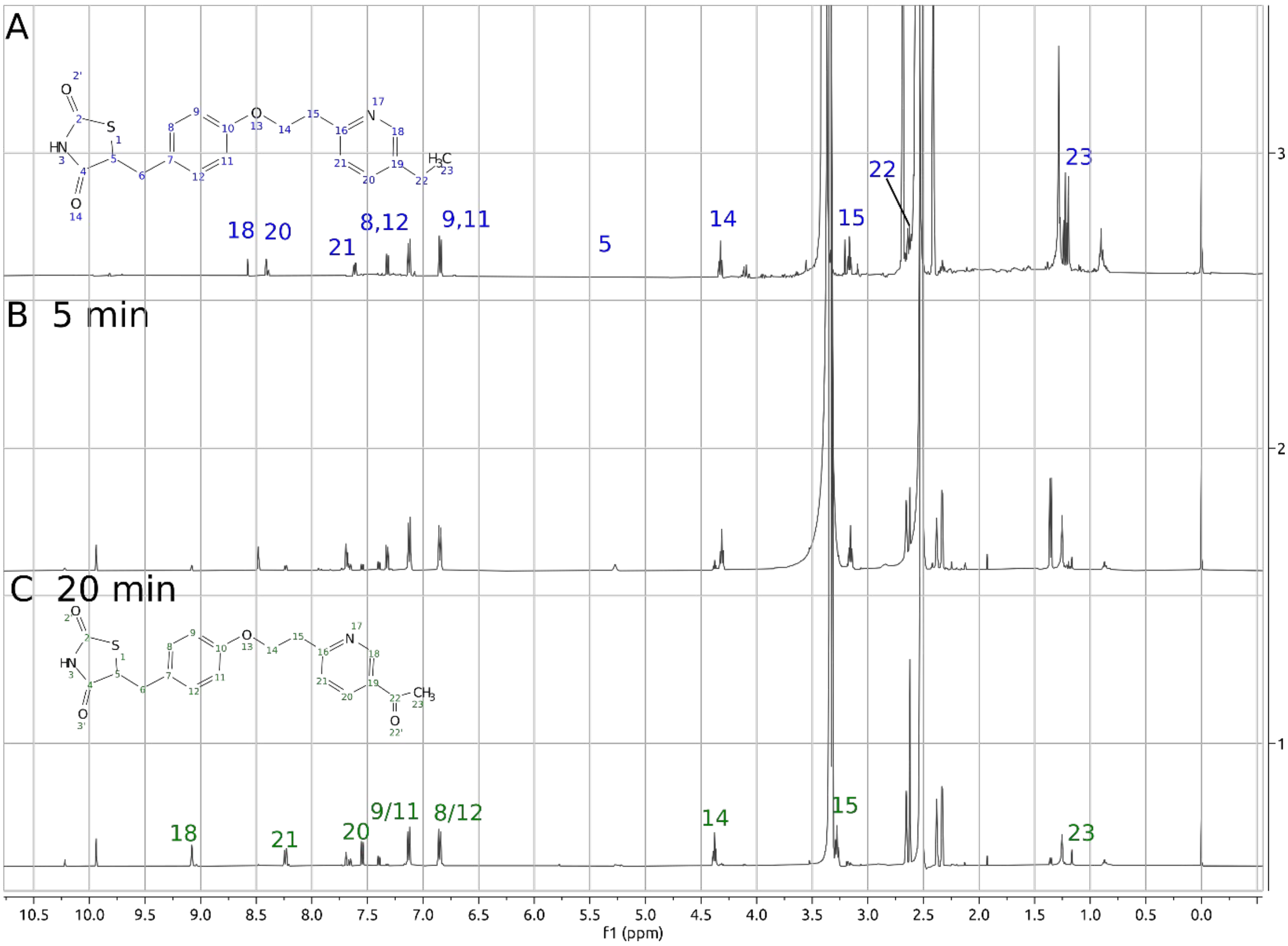

Figure S26: NMR of pioglitazone and the metabolites derived from BM3 DM. ^{1}H NMR spectra (500MHz, DMSO-d6) of 100 µM pioglitazone extracted from assay mix before (**A**) and 5 (**B**) and 20 minutes (C) after the addition of DM BM3 P450. Pioglitazone peaks are indicated by blue assignments and the product by green assignments. Pioglitazone assignment is based on Figures S18 and S19, and the product by comparison with this assignment and Figs S20 and S21. The thiazolium ring undergoes tautomerisation and exchange broadens the 5H resonance.

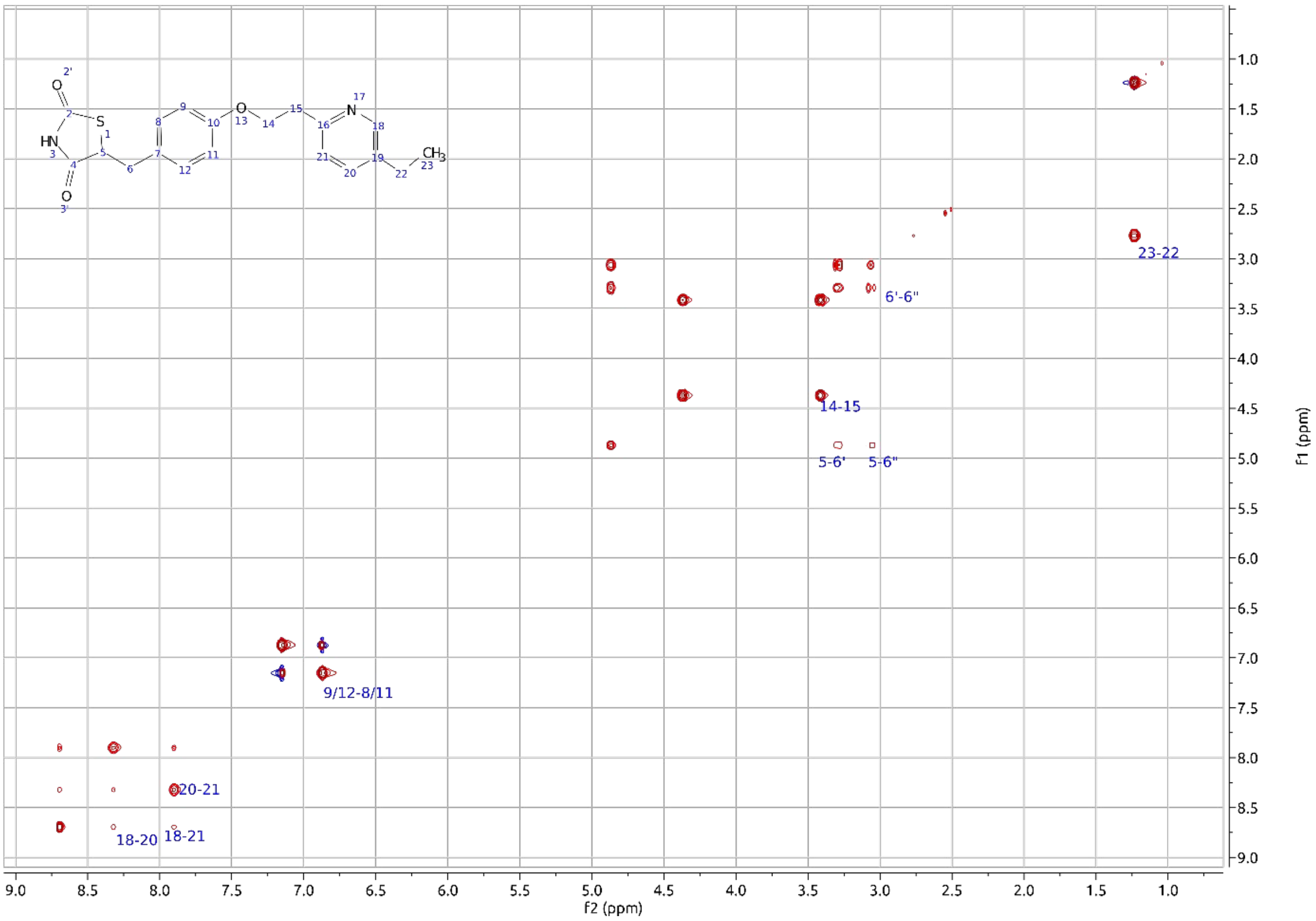

Figure S27: NMR elucidation of keto-pioglitazone. $^{1}H^{1}H$ TOSCY NMR spectrum of pioglitazone recorded at 500MHz with assignments labelled in blue.

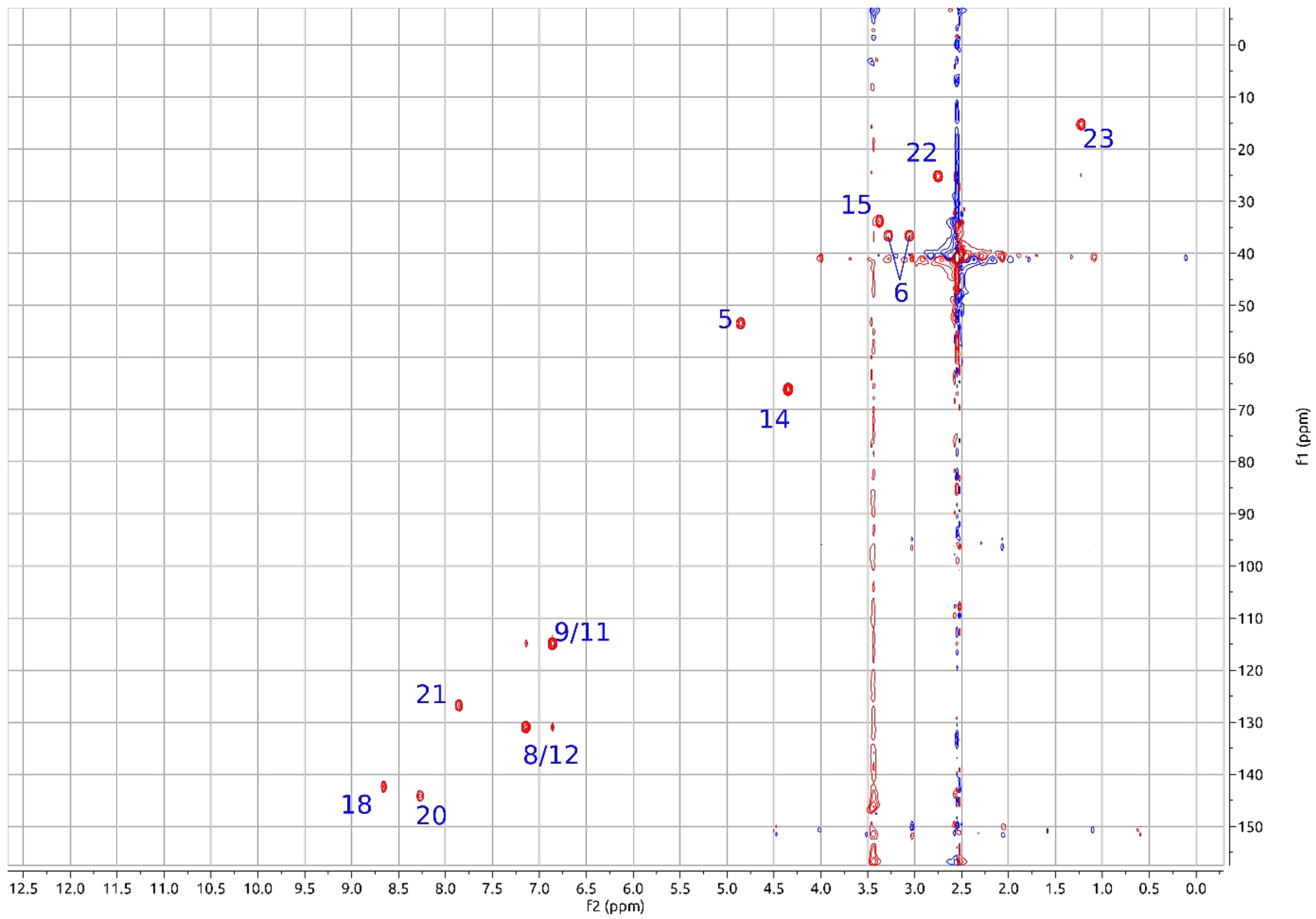

Figure S28: NMR elucidation of keto-pioglitazone. $^{1}H^{13}C$ HSQC NMR spectrum of pioglitazone recorded at 500MHz with assignments labelled in blue. δH (500 MHz, DMSO-d6) 8.65 (s, 1H, C18H), 8.27 (d, J = 2.4Hz, 1H, C20H), 7.85 (d, 2.4Hz, 1H, C21H), 7.13 (d, 8.5 Hz 2H, C8H, C12H), 6.83 (d, J = 8.5, 2H, C9H, C11H), 4.86 (C5H), 4.35 (t, J = 6.6 Hz, 2H, C14H2), 3.89 (t, J = 6.6 Hz, 2H, C15H2), 3.28 (t, J = 2.9 Hz, 1H, C6H), 3.05 (C6H), 2.72 (q? ?, 2H?, C22H2), 1.23(t, J = 8.5 Hz, 3H, C23H3).

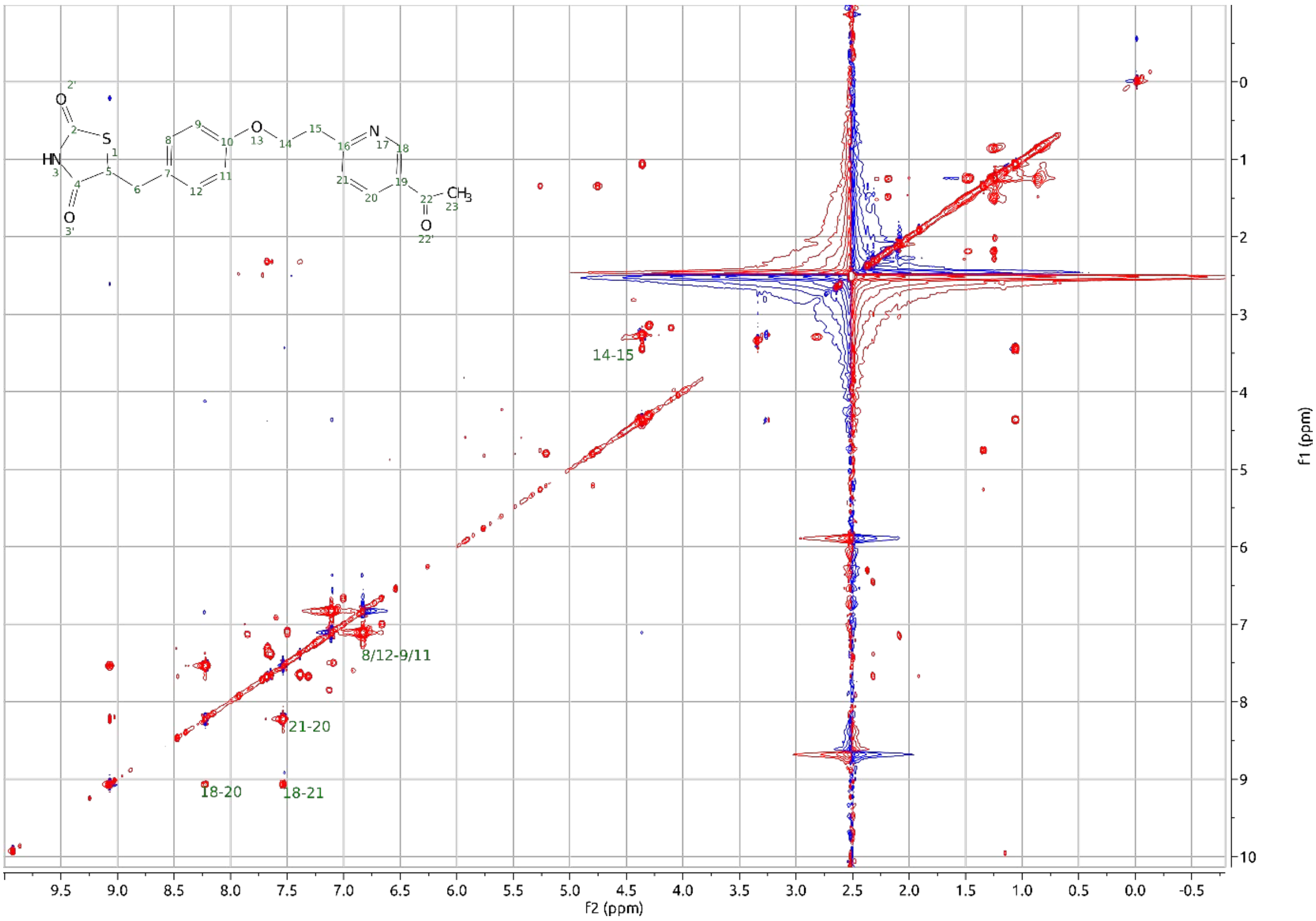

Figure S29: NMR elucidation of keto-pioglitazone. ^{1}H[1]H TOSCY NMR spectrum of 15m product of pioglitazone recorded at 500MHz with assignments labelled in green.

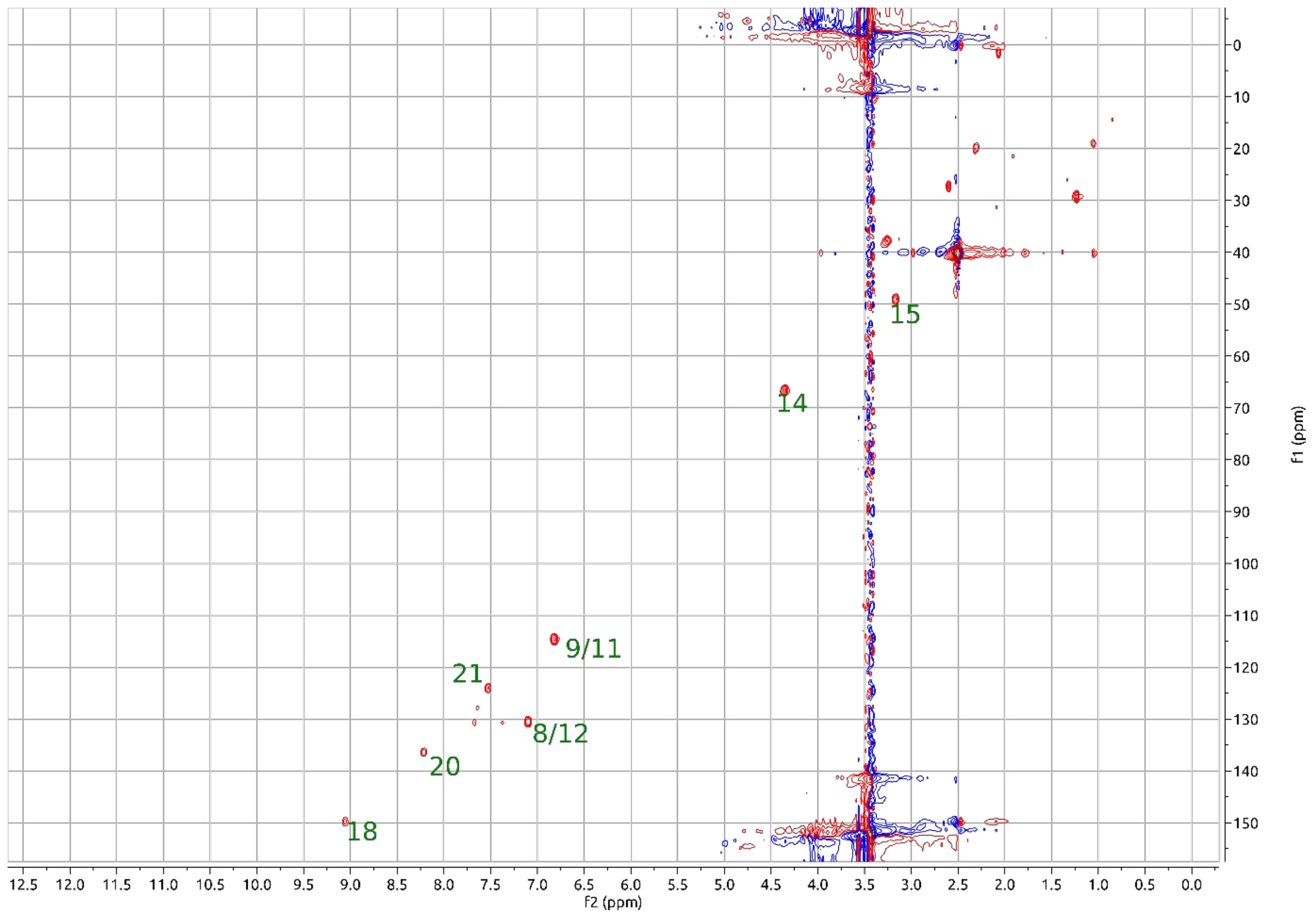

Figure S30: NMR elucidation of keto-pioglitazone. $^{1}H^{13}C$ HSQC NMR spectrum of the 15m pioglitazone product with assignments labelled in green. δH (500 MHz, DMSO-d6) 9.05 (s, 1H, C18H), 8.22 (d, J = 2.4Hz, 1H, C20H), 7.52 (d, 2.4Hz, 1H, C21H), 7.11 (d, 8.5 Hz 2H, C8H, C12H), 6.83 (d, J = 8.5, 2H, C9H, C11H), 4.34 (t, J = 6.6 Hz, 2H, C14H2), 3.27 (t, J = 6.6 Hz, 2H, C15H2).

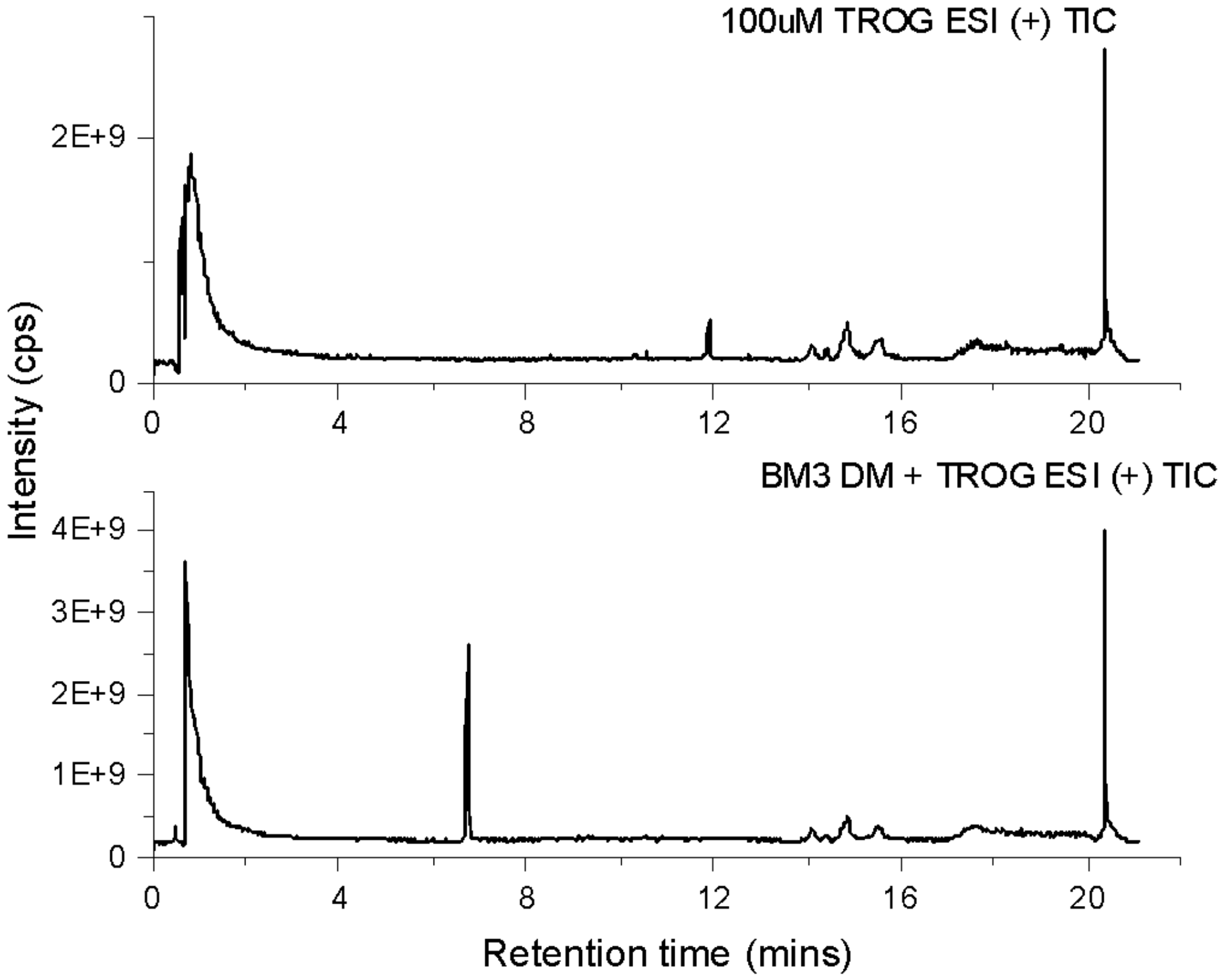

Figure S31: LC-MS peak chromatogram of BM3 DM + TROG turnover. Displaying mass range windows (±0.1 Da) for parent ion TROG. TROG + DM bulk incubation timepoint. A phenacetin internal standard peak is seen at RT 6.55.

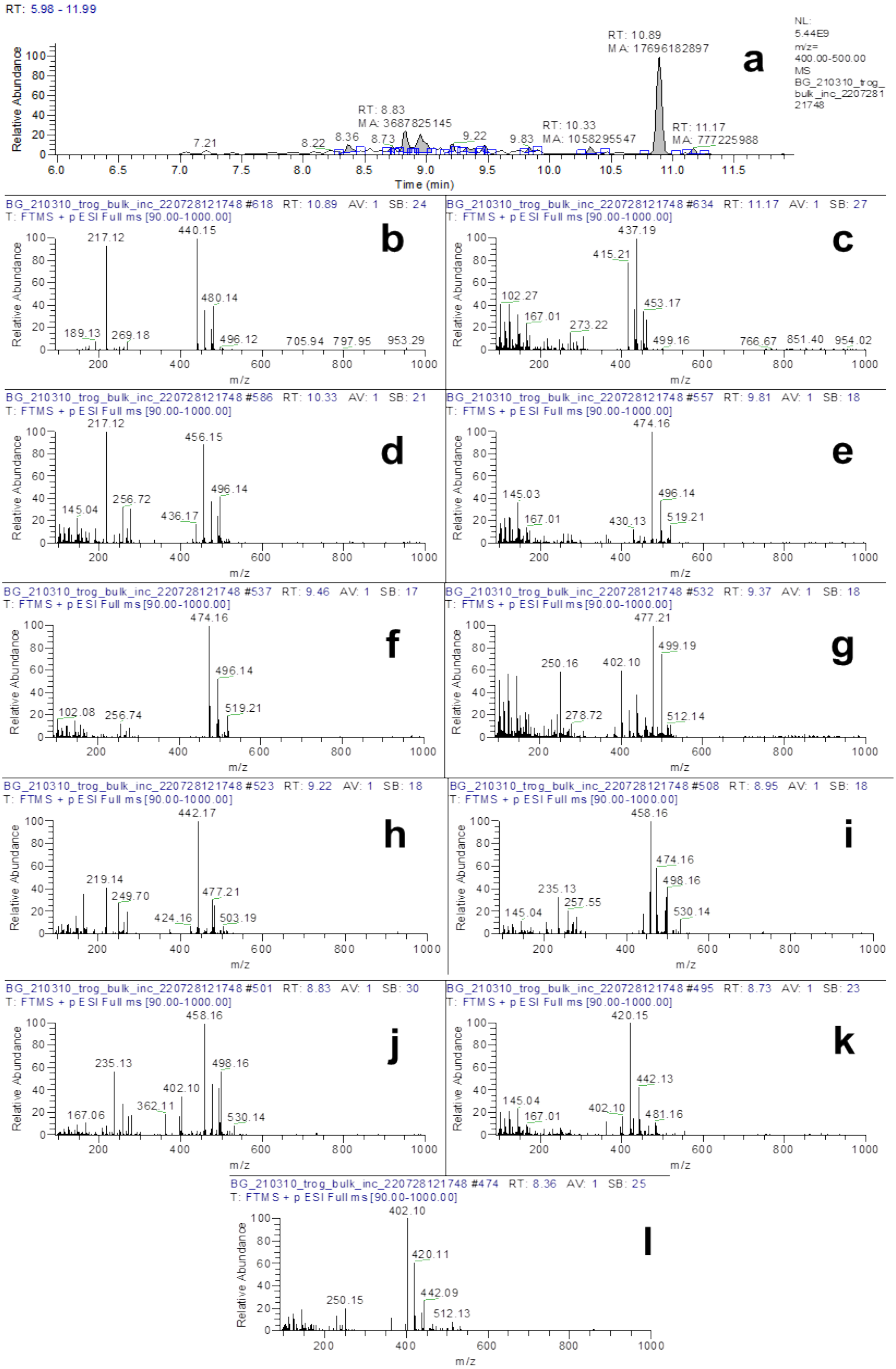

Figure S32: LC-MS/MS chromatograms of TGZ + BM3 DM incubations. Panel a shows TIC chromatogram with RTs and peak areas (MA) indicated for significant metabolites. Panel b shows ion chromatogram at RT 10.89 minutes, most abundant peak at *m/z* 440.2 indicates DM-TM1 $[M+H]^+$

oxidated metabolite species. Panel c shows ion chromatogram at RT 11.17 minutes, most abundant peak at *m/z* 437.2 indicates DM-TM5 unknown metabolite species. Panel d shows ion chromatogram at RT 10.33 minutes, most abundant peak at *m/z* 456.2 indicates DM-TM5 unknown metabolite species. Panel e shows ion chromatogram at RT 9.81 minutes, most abundant peak at *m/z* 474.2 indicates DM-TM6 $[M+H]^+$ dihydroxylated metabolite species. Panel f shows ion chromatogram at RT 9.46 minutes, most abundant peak at *m/z* 474.2 indicates DM-TM7 $[M+H]^+$ dihydroxylated metabolite species. Panel g shows ion chromatogram at RT 9.37 minutes, most abundant peak at *m/z* 477.2 indicates DM-TM8 unknown metabolite species. Panel h shows ion chromatogram at RT 9.22 minutes, most abundant peak at *m/z* 442.2 indicates DM-TM2 unknown metabolite species. Panel i shows ion chromatogram at RT 8.95 minutes, most abundant peak at *m/z* 458.2 indicates DM-TM3 $[M+H]^+$ monohydroxylated metabolite species. Panel j shows ion chromatogram at RT 8.83 minutes, most abundant peak at *m/z* 458.2 indicates DM-TM4 $[M+H]^+$ monohydroxylated metabolite species. Panel k shows ion chromatogram at RT 8.73 minutes, most abundant peak at *m/z* 420.2 indicates DM-TM9 unknown metabolite species. Panel l shows ion chromatogram at RT 8.36 minutes, most abundant peak at *m/z* 402.1 indicates DM-TM10 unknown metabolite species.

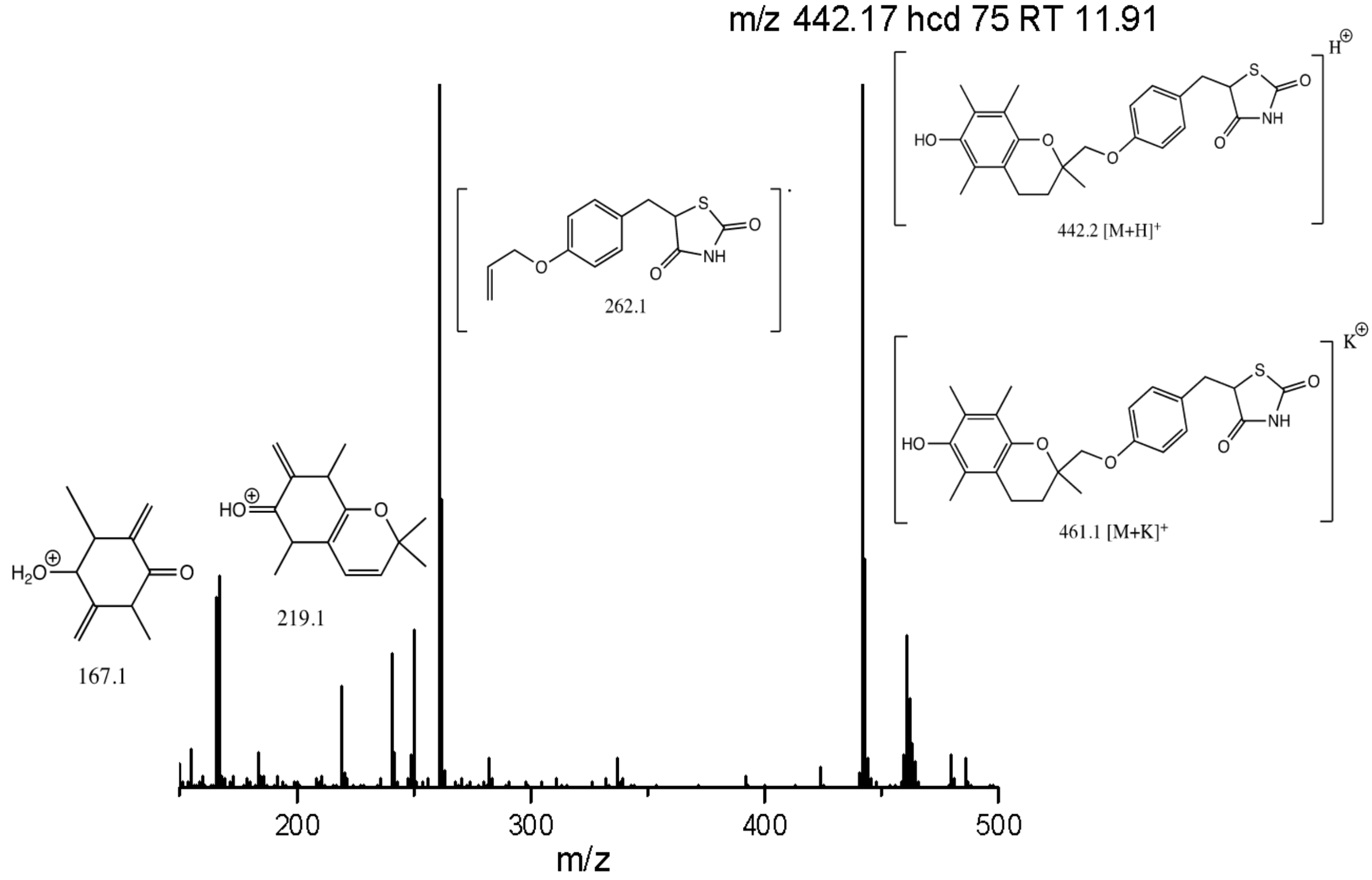

Figure S33: Troglitazone Standard MS. With Parent ion [M+H]+, potassium adduct [M+K]+ and three significant fragments labelled. Range of m/z set to 150- 500.

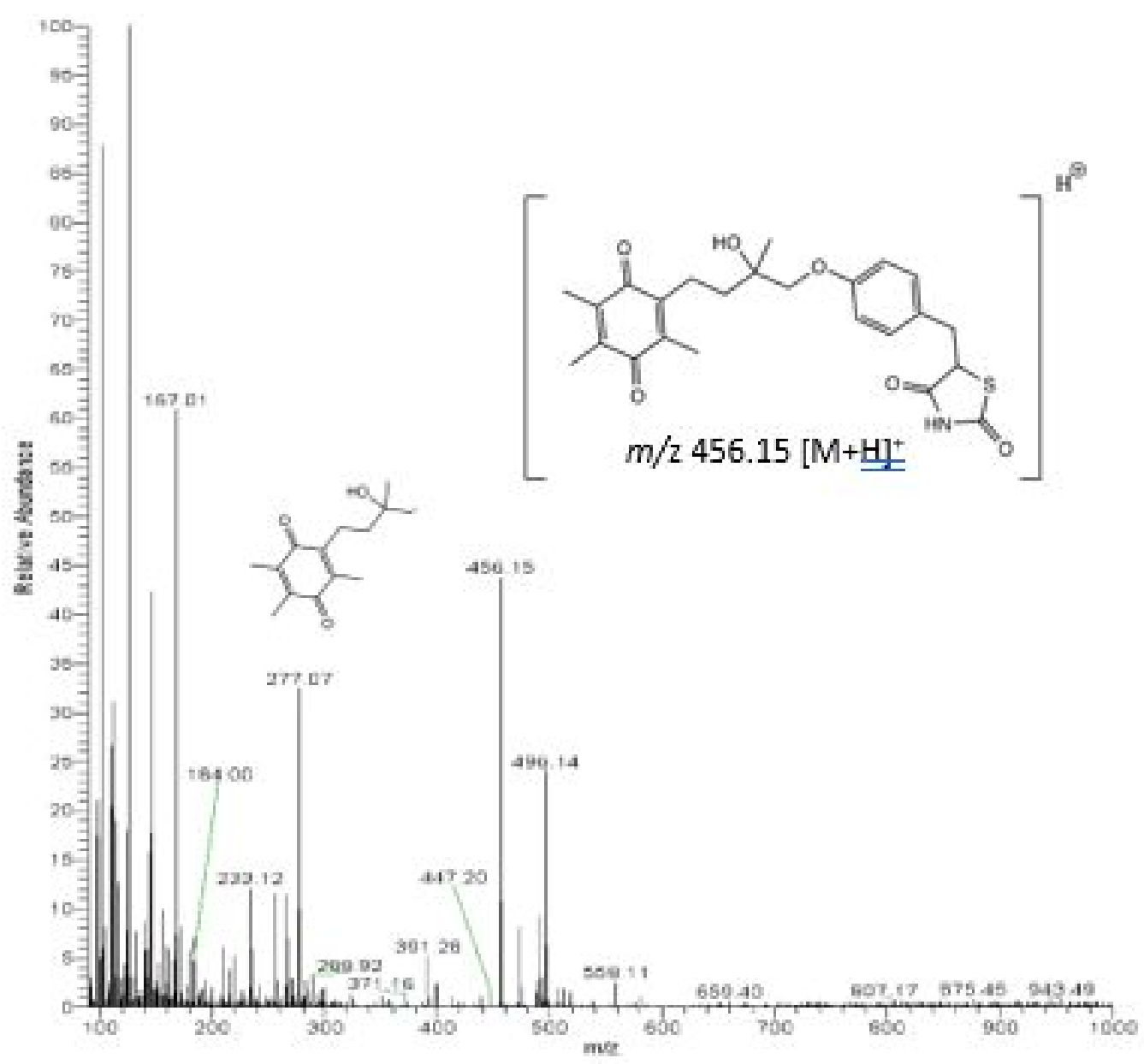

Figure S34: Troglitazone bulk incubation. Product m/z 456.15 hcd 75, RT 9.35. With structure of proposed m/z 456.15 peak at RT 9.36 and possible fragment.

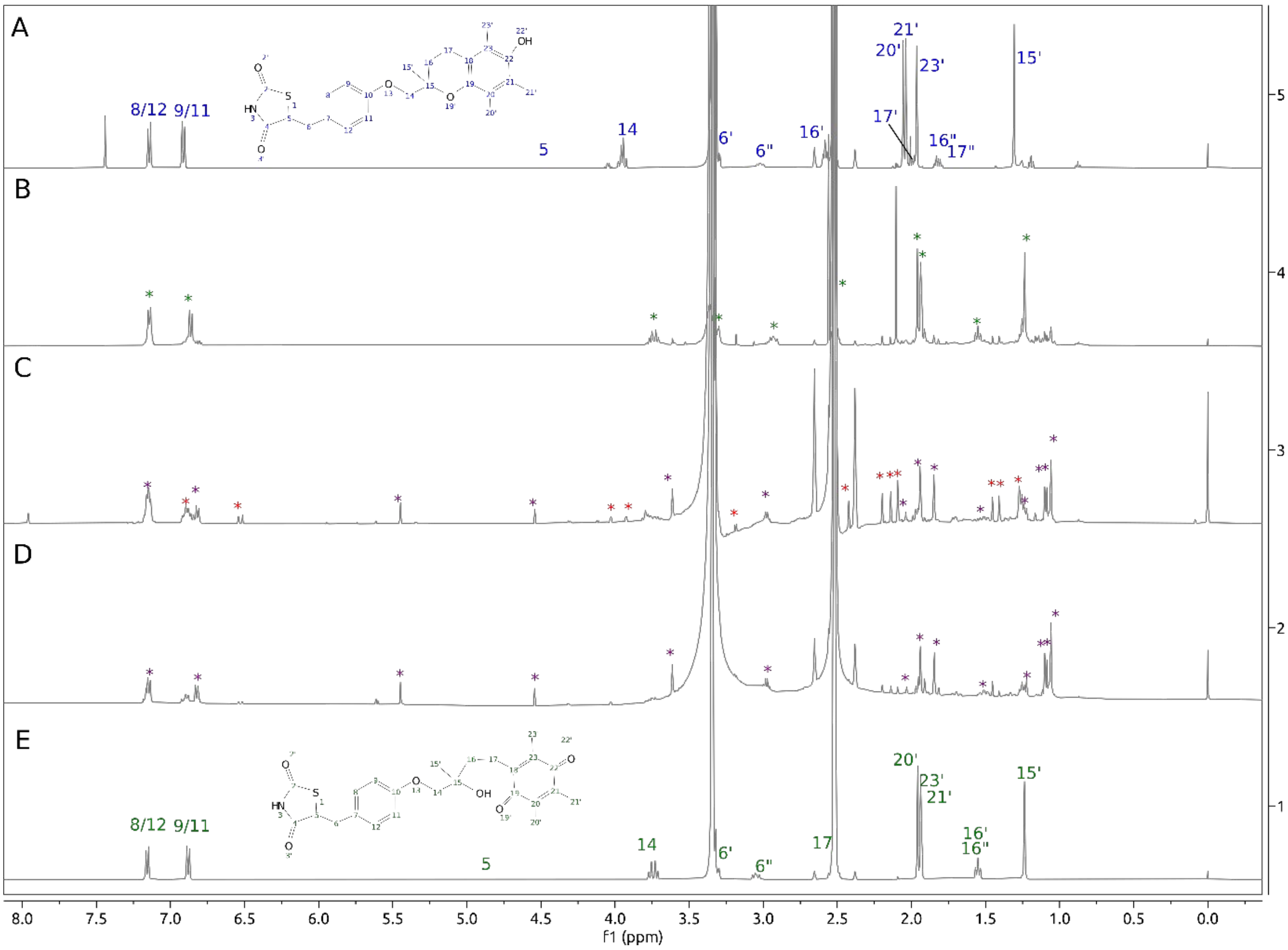

Figure S35: NMR of troglitazone and the metabolites derived from BM3 DM. ^{1}H NMR spectra (500MHz, DMSO-d6) of 100 µM troglitazone in DMSO (A) or after incubation with DM BM3 P450 (B), and then preparative HPLC fractions of the sample in B for M4 (C), M3 (D) and M1 (E). Peaks corresponding to the substrate (blue) or each metabolite are indicated by green (M1), purple (M3) or red (annotations). Troglitazone assignments are taken from Kassahun et al., 2000. The thiazolium ring undergoes tautomerisation and exchange broadens the 5H resonance.

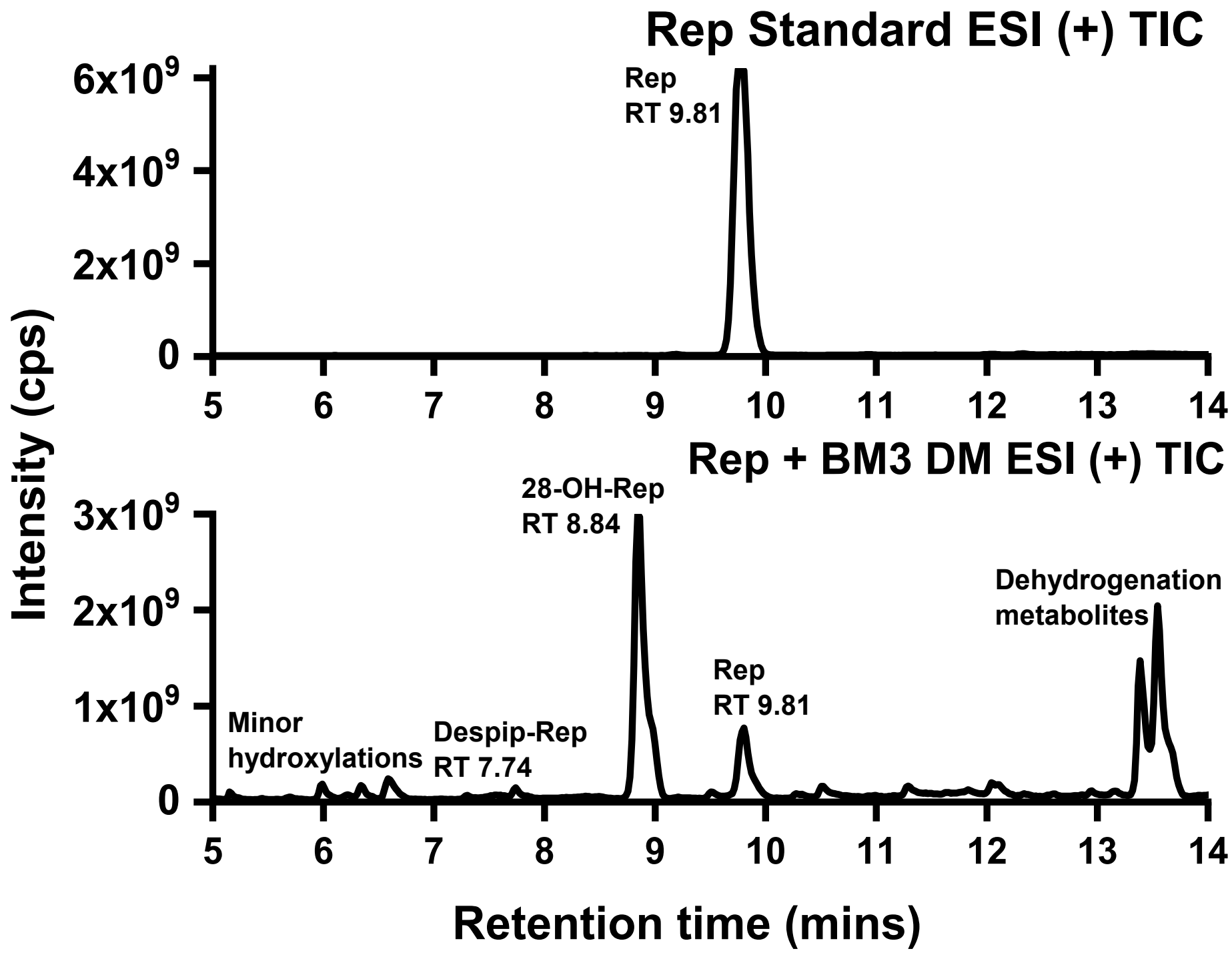

Figure S36: LCMS/MS Peak chromatogram of repaglinide standard and 20m repaglinide (Rep) + BM3 DM. Retention times and major metabolites shown.

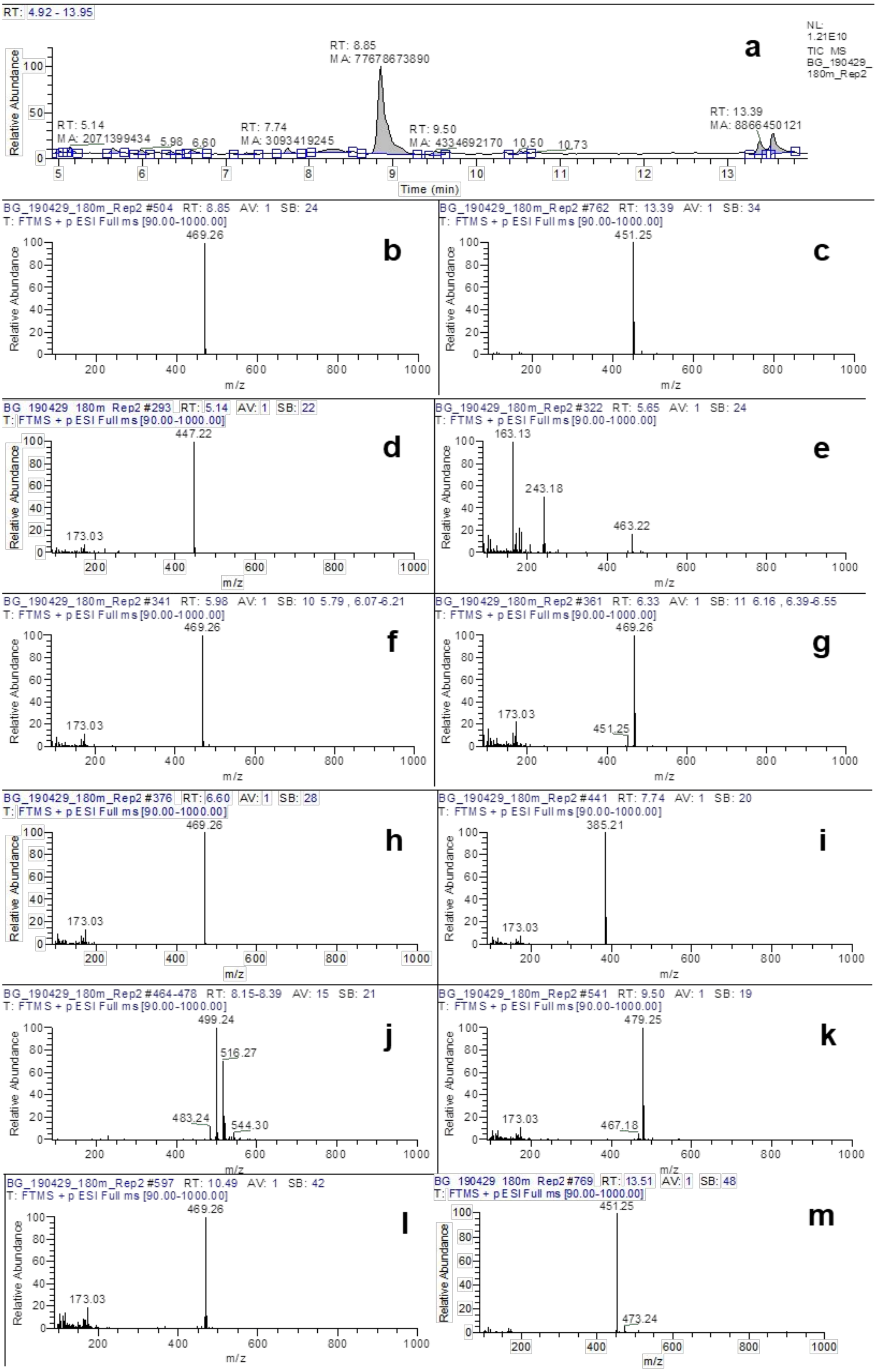

Figure S37: LC-MS/MS chromatogram of the 180 minute timepoint of REP + BM3 DM. Panel a shows TIC peak chromatogram with RTs and peak areas (MA) indicating significant metabolites. Panel b shows ion chromatogram at RT 8.85 minutes, most abundant species at *m/z* 469.3 is indicative of DM-ReM1 $[M+H]^+$ monohydroxylated species. Panel c shows ion chromatogram at RT 13.39 minutes, most

abundant species at *m/z* 451.3 is indicative of DM-ReM2 $[M+H]^+$ desaturated species. Panel d shows ion chromatogram at RT 5.14 minutes, most abundant species at *m/z* 447.2 is indicative of DM-ReM4 unknown metabolite species. Panel e shows ion chromatogram at RT 5.65 minutes, most abundant species at *m/z* 463.2 is indicative of DM-ReM5 unknown metabolite species. Panel f shows ion chromatogram at RT 5.98 minutes, most abundant species at *m/z* 469.3 is indicative of DM-ReM6 $[M+H]^+$ monohydroxylated species. Panel g shows ion chromatogram at RT 6.33 minutes, most abundant species at *m/z* 469.3 is indicative of DM-ReM7 $[M+H]^+$ monohydroxylated species. Panel h shows ion chromatogram at RT 6.60 minutes, most abundant species at *m/z* 469.3 is indicative of DM-ReM8 $[M+H]^+$ monohydroxylated species. Panel i shows ion chromatogram at RT 7.74 minutes, most abundant species at *m/z* 385.2 is indicative of DM-ReM9. Panel j shows ion chromatogram at RT 8.15-8.39 minutes, showing mixed metabolite species. Panel k shows ion chromatogram at RT 9.50 minutes, most abundant species at *m/z* 479.3 is indicative of DM-ReM10 unknown metabolite species. Panel l shows ion chromatogram at RT 10.49 minutes, most abundant species at *m/z* 469.3 is indicative of DM-ReM11 $[M+H]^+$ monohydroxylated species. Panel m shows ion chromatogram at RT 13.54 minutes, most abundant ion at *m/z* 451.3 is indicative of DM-ReM3 $[M+H]^+$ desaturated metabolite species.

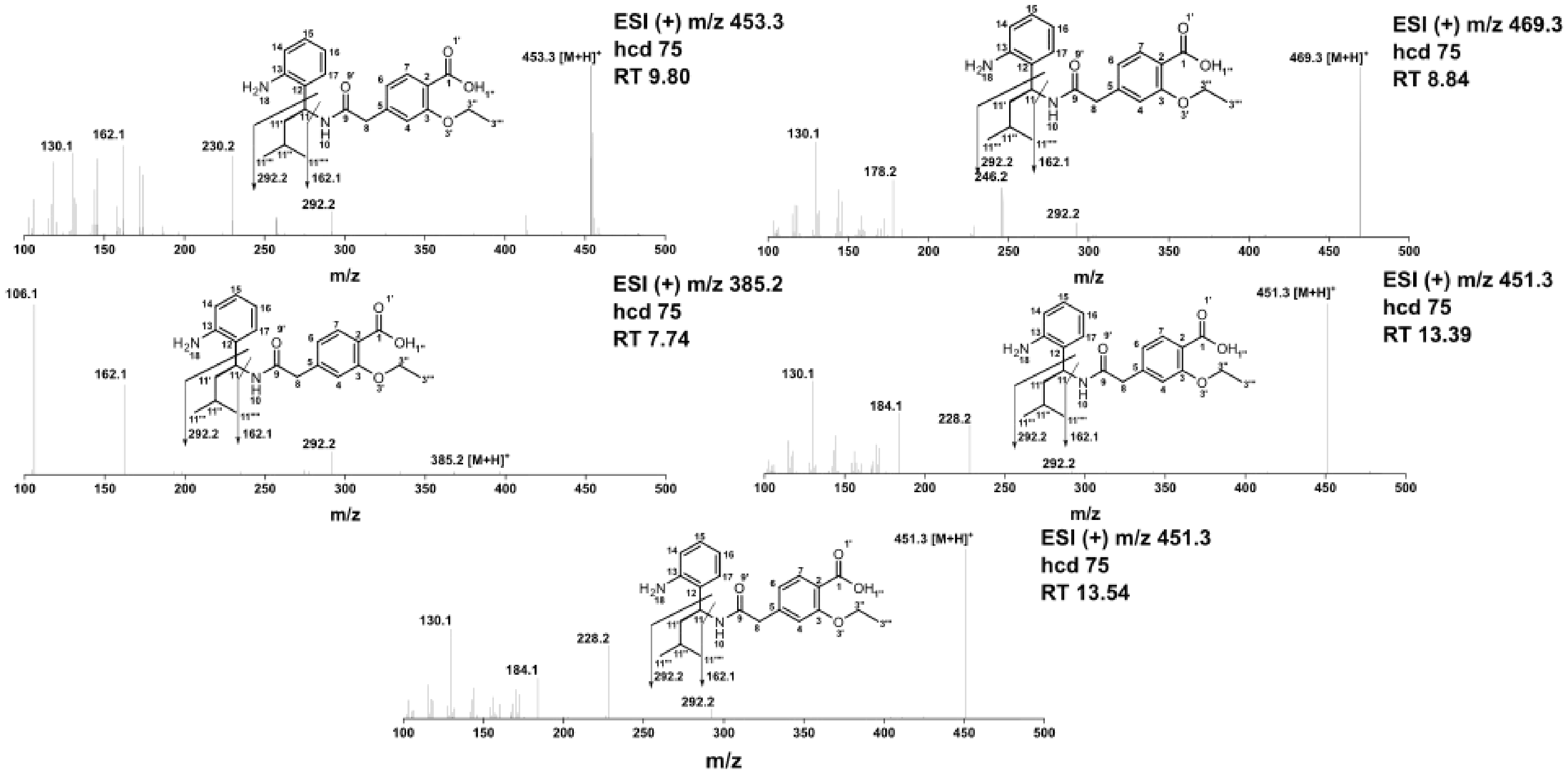

Figure S38: **Fragmentation of repaglinide metabolites.** ms/ms fragmentations of significant metabolites in Rep + BM3 DM 20 min sample.

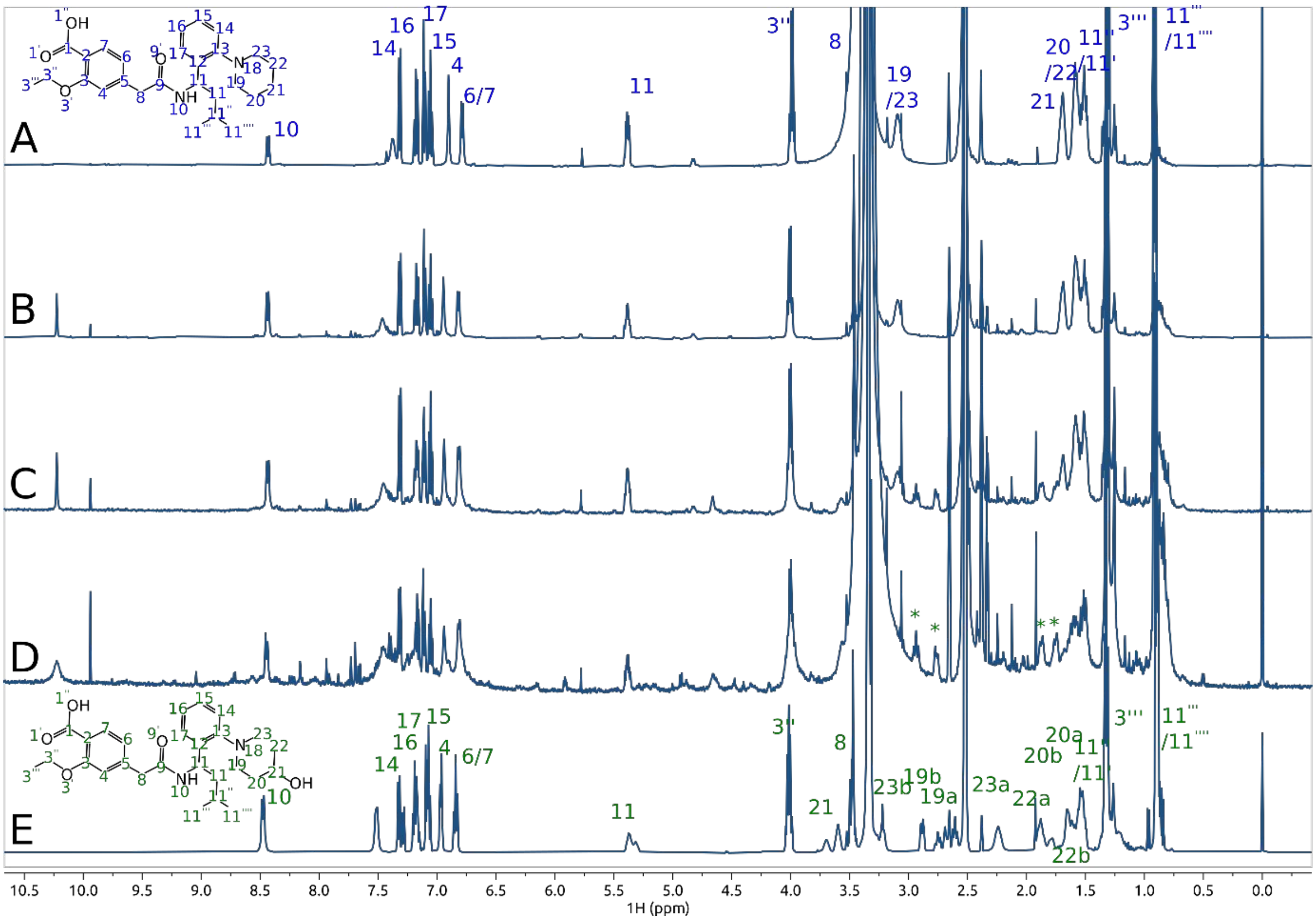

Figure S39: **NMR of repaglinide and the metabolites derived from BM3 DM.** 1H NMR spectrum (500MHz, DMSO-d6) of 100uM repaglinide before (A) addition of DM BM3 P450 and then 0 (B), 10, (C) and 180 (D) minutes after. (E) shows 1H NMR of 3-hydroxy-repaglinide standard. Green asterisks in B, C and D show generation of 21-hydroxy-repaglinide.

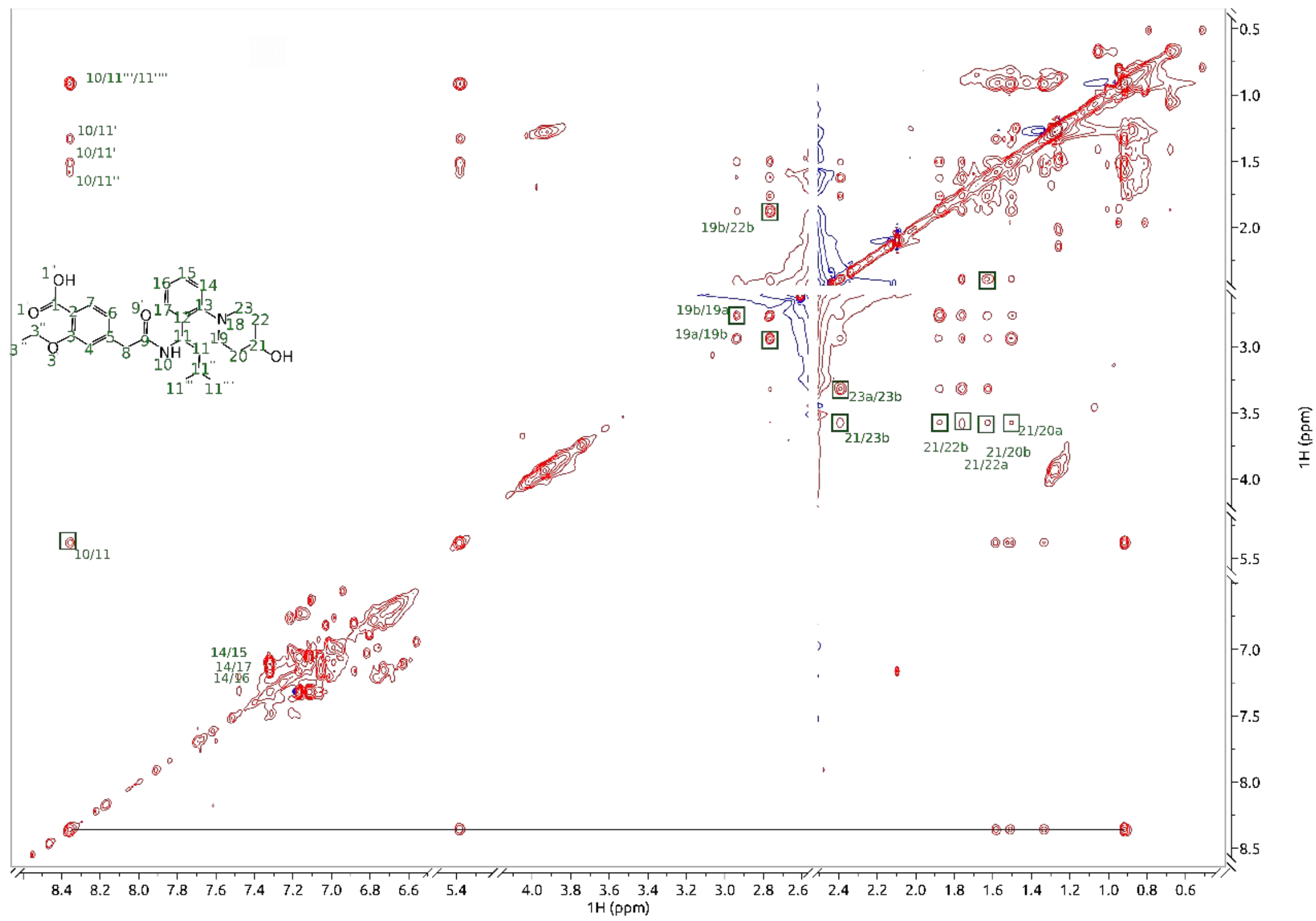

Figure S40: **NMR elucidation of 21-hydroxy-repaglinide.** 1H 1H TOCSY spectrum (800MHz DMSO-d6; mixing time 120ms) of the 10m repaglinide product (21’-hydroxy-repaglinide).

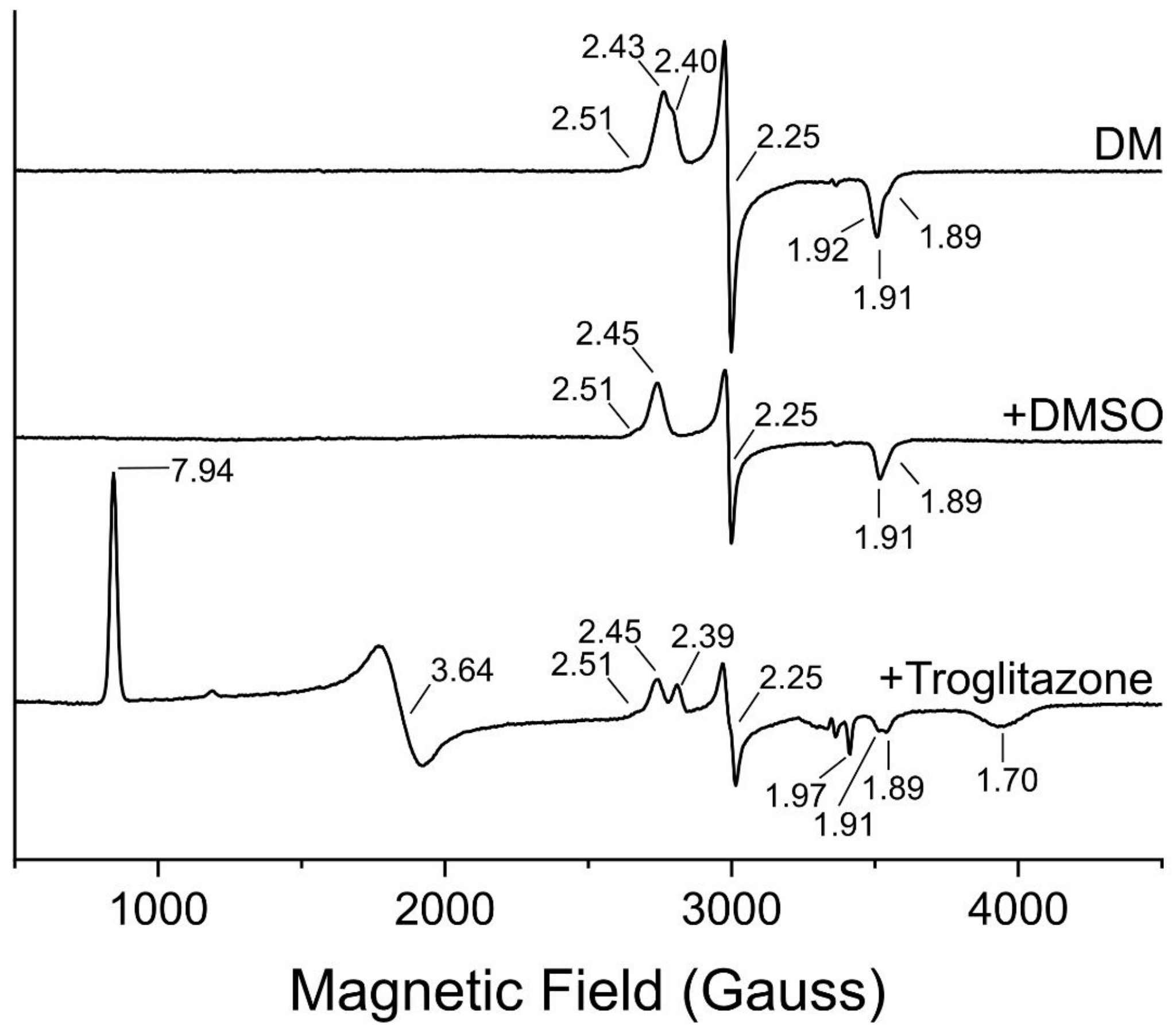

Figure S41: The EPR spectrum of the troglitazone-bound DM BM3 heme domain shows the development of a high-spin heme iron spectrum with g-values of 7.94/3.04/1.70 and other alterations in the low-spin spectrum induced by troglitazone binding to the P450 that are distinct from the low-spin native and DMSO-bound forms. All g-values are shown in Supplementary Table S3.
